# Using Consumer-Wearable Activity Trackers for Risk Prediction of Life-Threatening Heart Arrhythmia in Patients with an Implantable Cardioverter-Defibrillator: An Exploratory Observational Study

**DOI:** 10.3390/jpm12060942

**Published:** 2022-06-08

**Authors:** Diana My Frodi, Vlad Manea, Søren Zöga Diederichsen, Jesper Hastrup Svendsen, Katarzyna Wac, Tariq Osman Andersen

**Affiliations:** 1 Department of Cardiology, Copenhagen University Hospital-Rigshospitalet, 2100 Copenhagen, Denmark; diana.my.frodi.02@regionh.dk (D.M.F.); soeren.zoega.diederichsen@regionh.dk (S.Z.D.); jesper.hastrup.svendsen@regionh.dk (J.H.S.); 2 Department of Computer Science, Faculty of Science, University of Copenhagen, 2100 Copenhagen, Denmark; manea@di.ku.dk (V.M.); katarzyna.wac@unige.ch (K.W.); 3 Vital Beats ApS, 1434 Copenhagen, Denmark; 4 Department of Clinical Medicine, Faculty of Health and Medical Sciences, University of Copenhagen, 2200 Copenhagen, Denmark; 5 Quality of Life Technologies Lab, Center for Informatics, University of Geneva, 1227 Carouge, Switzerland

**Keywords:** implantable cardioverter-defibrillator, ventricular arrhythmia, consumer-wearable activity tracker, wearable, physical activity, sleep, heart rate, co-calibration, risk assessment, early detection

## Abstract

Ventricular arrhythmia (VA) is a leading cause of sudden death and health deterioration. Recent advances in predictive analytics and wearable technology for behavior assessment show promise but require further investigation. Yet, previous studies have only assessed other health outcomes and monitored patients for short durations (7–14 days). This study explores how behaviors reported by a consumer wearable can assist VA risk prediction. An exploratory observational study was conducted with participants who had an implantable cardioverter-defibrillator (ICD) and wore a Fitbit Alta HR consumer wearable. Fitbit reported behavioral markers for physical activity (light, fair, vigorous), sleep, and heart rate. A case-crossover analysis using conditional logistic regression assessed the effects of time-adjusted behaviors over 1–8 weeks on VA incidence. Twenty-seven patients (25 males, median age 59 years) were included. Among the participants, ICDs recorded 262 VA events during 8093 days monitored by Fitbit (median follow-up period 960 days). Longer light to fair activity durations and a higher heart rate increased the odds of a VA event (*p* < 0.001). In contrast, lengthier fair to vigorous activity and sleep durations decreased the odds of a VA event (*p* < 0.001). Future studies using consumer wearables in a larger population should prioritize these outcomes to further assess VA risk.

## 1. Introduction

Heart arrhythmias constitute a growing challenge to healthcare systems worldwide, and ventricular arrhythmias (VA) are an important and increasingly frequent cause of sudden death and health deterioration. Implantable cardioverter-defibrillators (ICDs) and remote monitoring have led to significant advances in reliably avoiding malignant VAs [1,2], leading to reduced hospitalization rates and improved quality of care [3,4]. Still, VAs are life-threatening and pose challenges for risk assessment, including prevention of inappropriate therapy and detection of impending events in time for proactive clinical intervention [5].

During the last decade, there has been a significant increase in the availability and use of health monitoring devices and mobile applications that provide users with personalized health data. The reasons for the use of such tools are diverse and include the desire for behavior change or health monitoring among healthy users as well as users with chronic diseases, such as cardiovascular disease [6,7]. Consumer-wearable activity trackers enable detailed monitoring of behavioral markers, including physical activity (intensity, frequency, volume, and type), sleep behavior, and rest-activity patterns [8]. These devices are currently spreading from the consumer market to the clinic, for example in cardiac rehabilitation [9] and arrhythmia monitoring [10,11]. Most studies using consumer-wearable activity trackers in cardiology have focused on heart failure or mortality [1,12,13,14]. Overall, studies have found that physical activity measured with activity trackers is an effective predictor of cardiovascular deterioration, with an inverse relationship between physical activity and the prognosis regarding morbidity, the stage of heart failure disease, cognitive function, intercurrent events such as hospitalization, and overall cardiovascular-related mortality [15]. However, previous studies using consumer-wearable activity trackers have not focused on VA event outcomes and on average have collected only seven days of activity tracker data [16].

This gap in the literature motivates further research into the usefulness of wearable activity trackers for heart arrhythmia risk prediction, and there is a specific need to conduct studies with prolonged periods of activity data collection. Based on prior studies, we hypothesize that consumer-wearable activity trackers represent an opportunity for improving early risk prediction of VAs and can potentially support early clinical intervention. We specifically aim to examine general behaviors among a patient population with ICDs over an extended period (from one week to several months) and thereby identify relationships between daily behaviors and VA events.

## 2. Materials and Methods

### 2.1. Study Settings and Objectives

An exploratory observational study was conducted to understand how VAs among patients with ICDs may be associated with behavioral data preceding a VA event, as collected from a consumer-wearable activity tracker. This study was part of a more extensive research and development project, SCAUT (Self-, Collaborative- and AUTo-detection of signs and symptoms of deterioration), conducted from 2014 to 2018, which aimed to improve early detection of deterioration in patients with a cardiac device and communication between such patients and health professionals [17,18,19,20]. The SCAUT project was completed at the Department of Cardiology at Copenhagen University Hospital - Rigshospitalet, Denmark.

### 2.2. Recruitment of Participants and Ethical Considerations

This study comprised a sample of 27 patients with a secondary prevention ICD, a device that is offered to individuals who have survived sudden cardiac arrest or who have a history of VAs [5]. Patients were recruited from the SCAUT project through a mix of purposive sampling and self-sign-up to ensure that participants were motivated to wear an activity tracker for a minimum of two months. Wearing the activity tracker was unrelated to patient treatment at the clinic. Patients were provided with and instructed to wear the Fitbit Alta HR (Fitbit Inc., San Francisco, CA, USA), a wrist-worn consumer-wearable activity tracker that reports daily measures of physical activity, sleep, and heart rate [21,22,23,24,25,26,27,28,29,30]. We further justify our choice of the Fitbit in Appendix A.1. Patients were asked to wear the wearable activity tracker as much as possible, day and night, for a minimum of two months.

As part of the SCAUT research and development project, this study was conducted according to the guidelines of the Declaration of Helsinki, approved by the Danish Data Protection Agency and reviewed by the Capital Region of Denmark’s Regional Committee for Health Research Ethics (No. H-19029475). Informed consent was obtained from all subjects involved in this study. Patients were informed that the Fitbit device is a consumer-wearable activity tracker and not a clinical device, and that it reports data without diagnostic validity.

### 2.3. Measured Outcomes

#### 2.3.1. ICD-Reported Outcomes

The first data source was the ICDs, which provided data report files on remote heart rhythm monitoring in XML format through Medtronic CareLink [31]. Three types of VA events were reported by the ICDs (Table 1) and reflected in the Mainspring^TM^ Report Export [31]: ventricular tachycardia (VT), ventricular tachycardia at two thresholds (VT1, VT2), and ventricular fibrillation into ventricular tachycardia (VF-VT). The incidence of a VA event (yes or no) during monitoring was used as the main outcome variable.

#### 2.3.2. Fitbit-Reported Data

The second data source was the Fitbit Alta HR (Fitbit Inc., San Francisco, CA, USA) consumer wearable activity tracker. The Fitbit data were collected through an application programming interface [32] that provided behavioral markers for physical activity, sleep, and heart rate in the CSV format. A data format example is available in Listing S1: Data Format Example. The markers reported by the Fitbit and used in this study were either raw (steps, heart rate) or processed according to Fitbit’s proprietary activity recognition algorithms (sedentary, physical activity, and sleep duration) [32].

The Fitbits counted participants’ steps and classified the physical intensity as sedentary, light, fair, or vigorous for each 15-min interval in a day (up to 96 intervals/day). For time periods of assumed sleep, the Fitbits classified the sleep type as asleep, awake, restless, or unknown for 1-min intervals (up to 1440 intervals/day). Fitbit did not provide precise thresholds for its physical activity recognition algorithms [33]. Thus, in this analysis, variables for cumulative adjacent intensities (e.g., light + fair) and variables for combinations of sleep types (e.g., awake + asleep) were derived. Sleep was not measured for all patients, and sedentary duration also included sleep. Therefore, all durations that included sedentary duration were deemed unreliable and excluded from analysis. The Fitbits also reported heart rate in 1-min intervals (up to 1,440 intervals/day). For the 15-min intervals, minimum, mean, median, maximum, and standard deviation (SD) heart rate values were derived from the 1-min heart rates. This additional step for the heart rate was necessary to derive the aggregate variables feasibly in time, while maintaining a high measurement frequency and aligning the heart rate intervals with those for the other behavioral markers. Therefore, all variables were derived for the 15-min intervals (Table 2).

### 2.4. Data Analysis

The Fitbit variables were aggregated for analysis. The variables were first aggregated over days, then weeks, then intervals of 1–8 consecutive weeks called periods. The reason for deriving different periods was to explore the risk of VA events for the purposes of timely clinical intervention (e.g., behaviors leading to events within one week vs. behaviors leading to events within eight weeks). Inferential and descriptive analyses using the aggregations were then conducted. The data analysis was performed in Python [34] using the Anaconda environment [35] (data aggregation and descriptive analysis) and in R [36] using the RStudio environment [37] and the Survival library [38] (inferential analysis). The data analysis code is available in Listing S2: Data Analysis Code. A similar approach, leveraging the aggregation of data for different periods before the event, has been proposed and evaluated as a co-calibration method [39].

#### 2.4.1. Data Quality Assurance and Data Aggregation

**Figure 1:**
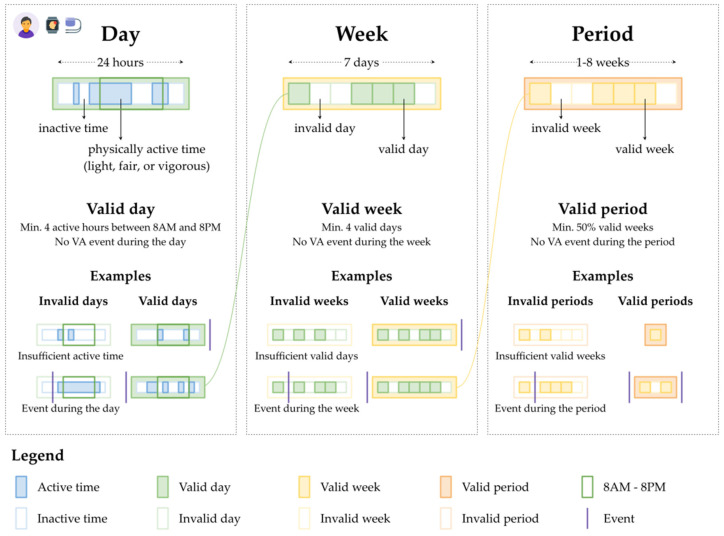
Data validation for days, weeks, and periods for one combination of scenarios: at least four hours of physical activity data between 8 a.m. and 8 p.m. for valid days, at least four valid days for valid weeks, and at least 50% valid weeks for valid periods. Days (depicted in green in the **top left**) contain physically active or inactive time (physically active time is depicted with solid blue, while physically inactive time is depicted with pale blue). If at least 50% of the time between 8 a.m. and 8 p.m. is active, the day is valid. Weeks (depicted in yellow in the **top center**) contain seven days (valid days are depicted with solid green, while invalid days are depicted with pale green). If the week has at least four valid days, that week is valid. Periods (in orange in the **top right**) contain 1–8 weeks (valid weeks are depicted with solid yellow, while invalid weeks are depicted with pale yellow). If at least 50% of the weeks of a period are valid, the period is valid. The figure then shows examples of valid and invalid days (**bottom left**), weeks (**bottom center**), and periods (**bottom right**).

Fitbit measurements reported as “0” were excluded from analysis. Valid days, weeks, and periods were then derived according to several scenarios. First, only days with at least one, two, four, or eight hours of physical activity data available (i.e., classified as any combination of sedentary, light, fair, or vigorous) between 8 a.m. and 8 p.m. were deemed valid days and included in the analysis as four separate scenarios. Second, only weeks with at least four, five, or seven valid days were included as valid weeks as three separate scenarios. Third, only periods with sufficient valid weeks were deemed valid periods according to three increasingly strict scenarios: minimum 50%, 75%, and 100% valid weeks (Figure 1). This system totaled 36 combinations of scenarios based on the four scenarios for valid days, three for valid weeks, and three for valid periods. We further elaborate on data validation in Appendix A.2.

**Figure 2:**
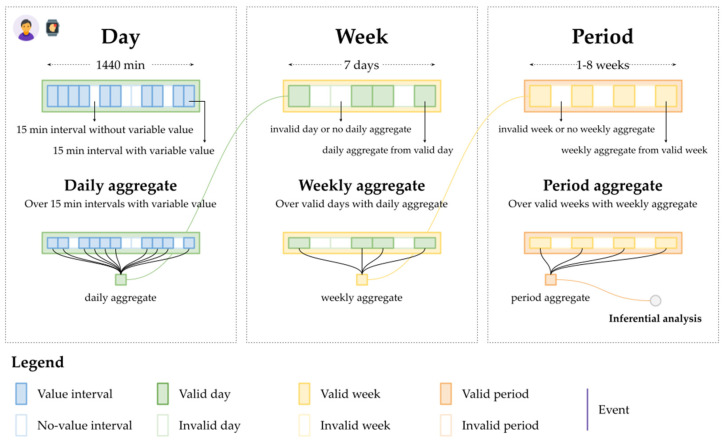
Data aggregations over valid days, valid weeks, and valid periods. A day (depicted in green in the **top left**) contains 96 15-min intervals (depicted in blue; the intervals with a value are solid blue, while those without a value are pale blue). A daily aggregate (in green in the **bottom left**) is constructed from the 15-min intervals with values. A week (depicted in yellow in the **top center**) contains seven days (in green; the valid days are solid green, while those invalid are pale green). A weekly aggregate (depicted in yellow in the **bottom center**) is constructed from the daily aggregates of the valid days. A period (in orange in the **top right**) contains 1–8 weeks (depicted in yellow; the valid weeks are solid yellow, while those invalid are pale yellow). A period aggregate (depicted in orange in the **bottom right**) is constructed from the weekly aggregates of the valid weeks. The period aggregate is then used for the inferential analysis.

Data aggregation was conducted through accumulation of all variables from the 15-min intervals in valid Fitbit days. Steps, physical activity duration, and sleep duration were summarized via daily aggregation to support the subsequent analysis. This approach has also been implemented in other studies using Fitbit data [39,40,41]. Daily heart rates were aggregated, and heart rates were reported by minimum, mean, median, maximum, and SD across the 15-min intervals. For each valid week, the mean daily count of steps, mean physical activity durations, mean sleep durations, and minimum heart rate were accumulated from the daily aggregations. The same aggregations were performed on valid periods using the aggregations on valid weeks (Figure 2).

#### 2.4.2. Analytic Design

The descriptive analysis consisted of two parts. The first part concerned summary statistics (median, mean, and SD) for data quality and behavioral markers. The second part concerned changes in Fitbit wear in the temporal vicinity of VA events observed for individual patients. For brevity, only the first part of the descriptive analysis is included in this paper. The second part is detailed in Appendix B.1.3.

The inferential analysis assessed the extent to which the given behaviors affected the odds of a VA event over time. This assessment was performed by means of a case-crossover design using conditional logistic regression [42,43]. This approach was chosen because it enabled meticulous analysis of cases of patients who had experienced a VA event; the patients served as their own controls.

**Figure 3:**
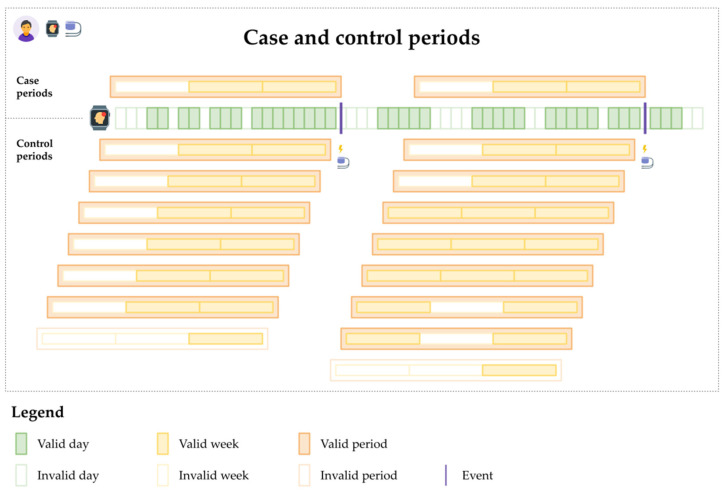
Case-crossover analysis: case and control periods for a combination of scenarios: an arbitrary scenario for valid days, at least four valid days for valid weeks, and at least 50% valid weeks for valid periods. Periods (depicted with orange contour) contain valid weeks (depicted with strong yellow contour and fill) and invalid weeks (depicted with pale yellow contour only). Week validity depends on having at least four valid days (depicted with strong green contour and fill) and at most three invalid days (depicted with pale green contour only). Case periods are followed by an event on the next day (depicted by a magenta vertical line). Events on the following day do not follow control periods. Valid periods (depicted with strong orange contour and fill) are either case periods (above the timeline of wearable monitoring) or control periods (below the timeline). Invalid periods are neither case periods nor control periods. The analysis uses all patients’ case and control periods.

For each patient, all monitoring days were used to capture the outcome of VA or no VA on any given day. Windows of valid Fitbit-measured periods of 1–8 weeks were used to define the exposure immediately succeeding each day of VA (case periods) or no VA (control periods). Figure 3 provides an example. In this way, the data were extended to include several time periods, one for each day of monitoring for each patient [44], and all patients with both VA events and Fitbit-measured behaviors contributed with cases, controls, or both to the analysis.

#### 2.4.3. Conditional Logistic Regression

Conditional logistic regression was used to assess how a one-unit change in behaviors (e.g., one extra minute of physical activity at a certain intensity or an extra beat per minute for the heart rate) affected the change in probability of a VA event. The predictors in the conditional logistic regression models were (a) behavior aggregate variables (*continuous exposure*) and (b) time-specific variables for time-point adjustment: (i) season (spring, summer, fall, winter), (ii) day of week (1–7), and (iii) weekday status (weekday, weekend day) of the date immediately succeeding the period. A *scenario* defines a specific combination of predictors: (a) behavior aggregate variables and (b) time-specific variables. Three conditional logistic regression formulae were derived. A total of 108 scenario-formula combinations resulted from 36 scenario combinations and three formulae (Table 3).

**Figure 4:**
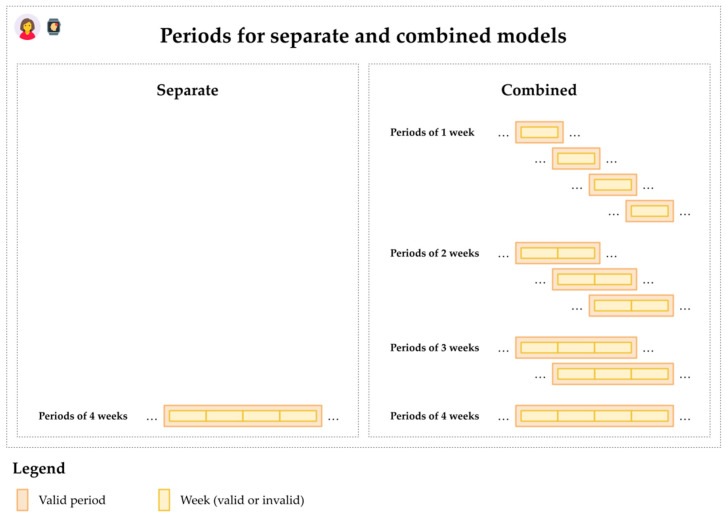
Periods in separate and combined models for a fixed duration of four weeks. Periods (depicted in orange) span across 1–8 weeks (depicted in yellow). For the separate models, only periods of precisely four weeks are included. For the combined models, periods of up to 4 weeks (precisely one week, and precisely two weeks, and precisely three weeks, and exactly four weeks) are included.

For each of the 108 scenario-formula combinations, conditional logistic regression models were created for periods of a fixed duration of 1–8 weeks (denoted as separate models) and for periods of durations in weeks at most the fixed duration 1–8 weeks, falling within the larger duration scope (denoted as combined models), as illustrated in Figure 4. A total of 108 × (8 + 8) = 1728 models resulted.

As the objective was to explore patterns without focusing on individual results, any odds ratio (OR) that exceeded the significance threshold ɑ = 0.05 was reported, without adjustments for multiple comparisons. However, highly significant ORs (e.g., *p* < 0.001) were expected. If, for a given behavior, across all models, (1) there were no significant ORs or (2) some significant ORs were sub-unit and some significant ORs were supra-unit, the OR was reported as inconclusive.

## 3. Results

### 3.1. Participant Information

Of the 65 heart patients with an ICD or an ICD with cardiac resynchronization therapy (CRT-D) who were invited to participate in the study, 27 participants provided written informed consent. Of these, 25 were male (93%), and the median age among participants was 59 years (mean 57.3 ± 11.1), as presented in Table 4.

### 3.2. Descriptive Analysis of Data Quality and Behavioral Markers

VA events were reported in 16 of the 27 participants. A total of 262 different types of VA events were recorded, with a mean of 16.4 ± 31.7/patient over a mean duration of 32.5 ± 28.9 months/person. Of these events, 56 were ventricular tachycardia (VT; mean 3.5 ± 8.8 events/patient), 172 were ventricular tachycardia type 1 (VT1; mean 10.8 ± 23.6 events/patient), and 34 were ventricular fibrillation and ventricular tachycardia (VF-VT) events (mean 2.1 ± 2.3 events/patient). The VA events by type for each patient are presented in Appendix B.1.1 (Table A1).

Fitbit-recorded behavioral data were available for all 27 patients with a total of 11.769 days (mean 435.9 ± 316.3 days/patient, median 357 days). The median ICD follow-up period was 960 days (mean 991.3 ± 880.9 days/patient). The follow-up periods for each patient are presented in Appendix B.1.1 (Table A2). The valid and invalid Fitbit days for each patient are also available in Appendix B.1.1 (Table A3, Table A4 and Table A5).

As previously mentioned, Fitbit-recorded behavioral markers for physical activity, sleep, and heart rate were collected for the 27 patients. Mean daily physical activity consisted of 7667.7 ± 3521.6 steps; 352.8 ± 89.8 min/patient in light activity duration; 43.4 ± 40.1 min/patient in fair activity duration; and 60.6 ± 33.0 min/patient in vigorous activity duration. For heart rate, the Fitbits recorded a mean of 50.3 ± 6.7 beats/min for the daily minimum, 69.3 ± 9.8 beats/min for the daily mean, a median mean of 66.2 ± 10.7 beats/min, a maximum mean of 124.9 ± 12.7 beats/min, and SD mean 4.2 ± 0.9 beats/min. More details are available in Appendix B.1.2 (Table A6, Table A7 and Table A8).

### 3.3. Inferential Analysis

**Figure 5:**
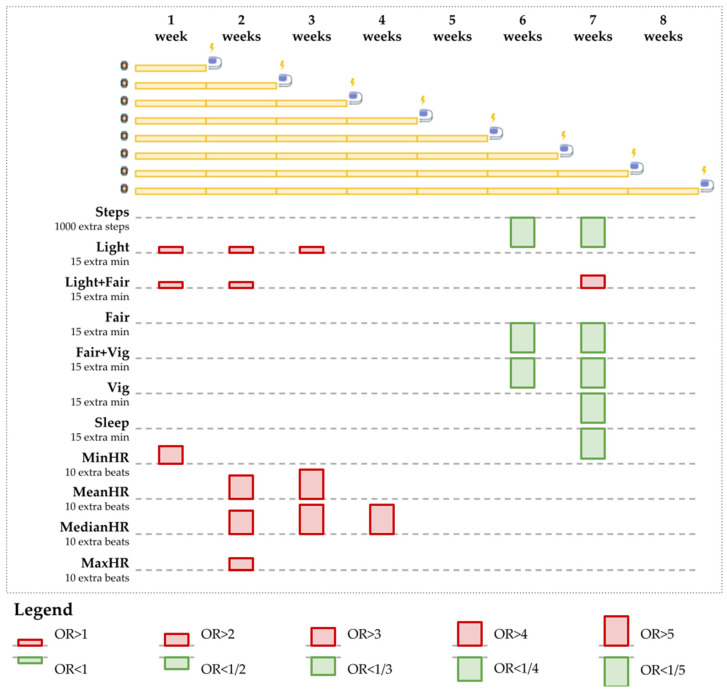
Odd ratios obtained from the separate models for 1000 extra steps, 15 extra minutes of physical activity or sleep, and 10 extra beats per minute for the heart rate. The separate Fitbit measurement periods of 1, 2, … 8 weeks are depicted on top. Below, informative estimations of the odd ratios are represented. For all results, *p* < 0.05. Periods in the separate models (depicted in yellow as sequences of weeks) are followed by potential events (depicted at the end of the yellow sequences). ORs are depicted as bars: OR > 1 (red and upwards) and OR < 1 (green and downward). Bar height corresponds to the distance between the OR and 1.

**Figure 6:**
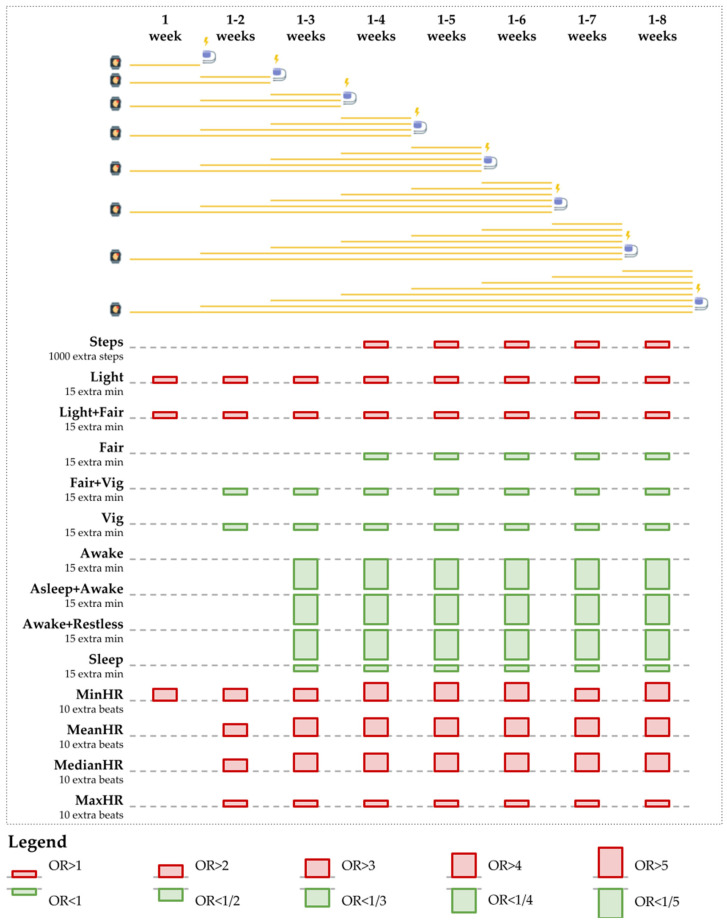
Odd ratios obtained from the combined models for 1000 extra steps, 15 extra minutes of physical activity or sleep, and 10 extra beats per minute for the heart rate. The combined Fitbit measurement periods of 1, 1–2, …, 1–8 weeks are depicted on top. Below, informative estimations of the odd ratios are represented. For all results, *p* < 0.05. All periods in the combined models (depicted in yellow as sequences of weeks and stacked for each maximum duration of 1, 2, …, 8 weeks) are followed by potential events (depicted at the end of the yellow sequences). ORs are depicted as bars: OR > 1 (red and upwards) and OR < 1 (green and downwards). Bar height corresponds to the distance between the OR and 1.

Increases in light to fair physical activity duration and in heart rate resulted in an increased risk probability of a VA event; conversely, increases in fair to vigorous physical activity duration and sleep duration that included the awake sleep type (i.e., asleep + awake) resulted in a decreased risk probability of a VA event (Figure 5 and Figure 6 and Table 5).

Spending more time in light to fair physical activity increases the risk of VA events. On average, the odds increase by 9 to 20 percent when time spent in light-intensity physical activity increases by 15 min per day, as measured over 1–3 weeks. Furthermore, 15 additional minutes of light or fair activity led to an average odd increase of 9 to 12 percent, as measured over 1–2 weeks.

However, fair to vigorous activity reduces the risk of VA events. The odds decreased by 32 to 34 percent on average with every 15 extra minutes of fair activity per day, measured over 4–8 weeks. The odds also decreased by 16 to 21 percent on average with every 15 extra minutes of vigorous activity, measured over 2–8 weeks. Furthermore, 15 more minutes of combined fair and vigorous activity reduced the odds by 12 to 16 percent, as measured over 2–8 weeks.

A higher heart rate increases the risk of VA events. Ten extra beats per minute increase the odds of a VA event as follows: minimum heart rate measured over one week doubled the odds, mean heart rate measured over 2–3 weeks increased the odds four to ten times, median heart rate measured over 2–4 weeks increased the odds four to sixteen times, and maximum heart rate measured over two weeks doubled the odds of a VA event. More findings are available in Appendix B.2 (Table A9, Table A10, Table A11 and Table A12).

## 4. Discussion

### 4.1. Key Findings Compared to Prior Work

This exploratory observational study assessed the relationship between behavioral activity changes and the risk probability of potentially life-threatening VA events by comparing data from ICDs and Fitbit wearable activity trackers. We found that higher heart rates and spending more time in light to fair physical activity increased the risk of imminent VA events, whereas fair to vigorous activity reduced the risk. Few previous studies have assessed the risk probability of VA events using technology-reported data from ICDs and wearable activity trackers, especially with longer follow up times. By assessing the utility of consumer-grade wearable activity trackers for early risk assessment of VA events, the aim is to build towards the validation of interpreting significant behavior changes (activity levels, sleep) as a vital clinical sign for early clinical intervention among patients at risk of life-threatening heart arrhythmias.

Our cohort was representative of an ICD population with regards to age and gender, with a predominance of males. The results indicated that increased duration of light or light + fair physical activity increased the risk probability of a VA. Conversely, a decrease in fair, fair + vigorous, or vigorous activity levels increased the risk probability of a VA. Our results therefore support a cardioprotective effect of exercise [45] and suggest that there is an increased risk of developing arrhythmia with decreased activity levels. Few previous studies have focused on the outcome of VA and its relationship to physical activity measured by an ICD device [46]. For example, in one study among an all-female cohort, ICD device-measured physical activity started to decline 16 days before a VA and defibrillator shock [47]. Moreover, declining physical activity has been previously used as a predictor for outcomes such as heart failure and mortality [46]. An inverse relationship between activity level and cardiovascular events has been found using a wearable activity tracker for activity monitoring in a cohort without a prior or concurrent cardiovascular disease [48].

Based on our findings, future studies could measure light physical activity over shorter time intervals (1–3 weeks). They may consider 9 percent as a baseline odd increase for 15 extra minutes of light to fair activity. Fair and vigorous physical activity could be measured between 2 and 8 weeks, where future studies may consider the average odds decreases of 32% and 16% for every 15 extra minutes of these activity intensities. Heart rate yielded significant effects within 1–3 weeks of monitoring. Ten extra beats per minute increased the odds twofold on average. Heart rate could be monitored closest, as changes associated with the minimum and maximum heart rates were visible in short periods (one and two weeks, respectively).

This study does not report key findings related to sleep. This choice was made because we noted that Fitbit may not always have reported sleep durations for the patients, as the awake, sleeping, and non-wear times were accumulated in the dataset. Nevertheless, prior literature has described an association between sleep behavior and physical activity for patient-reported physical function, quality of life, and cognitive function, though often with the limiting factor of self-reported sleep outcomes in homogeneous populations [49,50]. As there are interactions between physical activity and sleep throughout the day [51], we suggest that sleep measurements should be included in future studies; the validation of technology-reported sleep duration with self-reported sleep duration could ensure realistic measurements.

### 4.2. Implications for Designing Systems for Ventricular Arrhythmia Risk Assessment Using Wearable Activity Data

The results from this study and similar previous studies suggest that several critical aspects may influence the quality of data collected and pose potential scaling issues when leveraging consumer-wearable activity trackers for VA risk assessment among ICD patients. Although the results are indicative, they suggest that consumer-wearable activity trackers can be leveraged for VA event risk assessment, potentially enabling better (self-) management of activities contributing to health (especially physical activity), and may ultimately lead to improved health outcomes.

There are several implications for systems designed for risk prediction of VAs. First, there is, on the one hand, the potential derived from using external research-grade wearable activity trackers, which can capture high-granularity and high-accuracy data [52]. Such devices have produced more accurate measures of daily activity compared to activity tracking using accelerometers embedded in ICDs, which is limited to daily summaries of physical activity [53]. On the other hand, the consumer-wearable data must be accurate enough for the clinical purpose; in our case, the sleep datasets were deemed unreliable. Second, in the larger context of current developments, comparisons between research-grade and consumer-wearable activity trackers have shown strong validity, although the validity ranged widely between devices [25]. Third, there is a need for consensus on many levels regarding the use of wearable accelerometers, such as ways to manage the differences among proprietary algorithms for behavioral markers [15,16]. Finally, to identify implications for technology and system design, exploration of the benefits of long-term use of external activity trackers is eminent.

For patients who accept consumer-wearable activity trackers, the accuracy of the predictive performance and the timeliness of notifications are critical for the success of usage and collected data quality [54]. In an era of remote, decentralized, and increasingly personalized patient care, our results indicate that physical activity measured through consumer-wearable activity trackers can play a role in cardiovascular event risk assessment. However, there is an evident need for more extensive, prospective, and well-designed studies to quantify the utility of physical activity as a vital signal of clinical deterioration and VA.

### 4.3. Strengths and Limitations

A strength of this study is that it is the first to examine the outcome of VA, using raw data from ICDs and compare it to data from a consumer-wearable activity tracker. Second, the activity tracker wear time was on average over one year, which far exceeds the average wear time in previous studies [16]. Third, the wearable trackers measured multiple daily behaviors continuously, allowing behaviors to unfold over extended periods of time. Fourth, an accurate, user-friendly, and data collection-friendly device was used. The selected device may have positively contributed to the data quality to a greater extent possible than other consumer-wearable activity trackers [19,20].

This study has several limitations. First, the small sample size of unique patients prevents a separate analysis based on age or gender and limits our confidence regarding the generalizability of the findings to a larger population. The results are based on a large amount of longitudinal data with several time epochs per individual patient. This approach poses a risk of bias by carry-over, but arguably resulted in a conservative analysis given that any association between exposure and outcome had to be robust to nullify inverse or null associations during parts of the same time epoch. We allowed a permissive significance threshold of ɑ = 0.05 without adjustments for multiple tests, but also found highly significant results (e.g., *p* < 0.001). These results would therefore remain visible with statistically significant corrections up to 50×. Furthermore, patient-effect was adjusted for by means of distinguishing each behavior-time-event data point as unique to the patient, and no additional variables could be added, avoiding collinearity.

Second, the definitions of behaviors reported by Fitbit, specifically the thresholds in the Fitbit activity recognition algorithm, are unknown for different physical activity intensities, as well as for sleep. This limitation was accounted for by including cumulative variables for physical intensities (e.g., light + fair) and sleep (e.g., asleep + awake). In addition, Fitbit did not distinguish sedentary duration as time awake, sleeping, or non-wear. This limitation made it difficult to delineate and thereby analyze these behaviors and may explain the mean recorded sleep duration of under four hours. This limitation was accounted for by excluding the sedentary duration from the analysis.

Third, feedback about behaviors (e.g., visualizations of the number of steps) and observations reported to patients by the Fitbit device and associated application might have influenced the behaviors under study. The patients may have changed their physical activity patterns, or sleep patterns based on the feedback provided by the device.

Finally, the patient data did not contain baseline characteristics—such as concurrent heart disease, presence of heart failure, medications, or comorbidities (e.g., hypertension or diabetes)—that may be confounders, influencing the behavioral, as well as the VA outcomes.

## 5. Conclusions

In the light of the increased availability and reliability of consumer-wearable activity trackers, this study explored the extent to which daily behaviors reported by such trackers can assist in VA risk assessment in ICD patients. The results indicated that increased levels of activity are cardioprotective, decreasing the odds of experiencing a VA event. Future studies using consumer-wearable activity trackers in a larger population can further refine our findings to assess the risk of VA.

## Figures and Tables

**Table 1:** VA event types reported by the implanted ICDs.

VA Event Type	Description
VT	Ventricular tachycardia is a very fast heart rhythm that begins in the ventricles. It is defined as a heart rate of more than 100 beats/min with at least three irregular heartbeats in a row.
VT1	Ventricular Tachycardia Zone 1: Medtronic has an option to divide VTs into heart-rate zones. This division allows physicians to program different treatments for the different zones. For example, Zone 1 may range from 100 to 180 beats/min.
VT2	Ventricular Tachycardia Zone 2: Zone 2 is similar to VT1, but with a different beat-per-minute interval.
VF-VT	Ventricular fibrillation into ventricular tachycardia: VT is potentially lethal, VF even more so. In ventricular fibrillation, the ventricular rates are higher than in VT.

**Table 2:** Fitbit measurements and derived variables with abbreviations and units.

Measurement		Derived Variable		
Name	Unit	Name	Abbreviation	Unit
Steps	count	Steps	Steps	count
Sedentary	yes/no	(Excluded from analysis)		
Sedentary, Light	yes/no	(Excluded from analysis)		
Light	yes/no	Light activity duration	Light	min
Light, Fair	yes/no	Cumulative light and fair activity duration	Light + Fair	min
Fair	yes/no	Fair activity duration	Fair	min
Fair, Vigorous	yes/no	Cumulative fair and vigorous activity duration	Fair + Vig	min
Vigorous	yes/no	Vigorous activity duration	Vig	min
Light, Fair, Vigorous	yes/no	Cumulative active duration	Active	min
Asleep	yes/no	Asleep sleep duration	Asleep	min
Awake	yes/no	Awake sleep duration	Awake	min
Restless	yes/no	Restless sleep duration	Restless	min
Unknown	yes/no	(Excluded from analysis)		
Asleep, Awake	yes/no	Cumulative asleep and awake sleep duration	Asleep + Awake	min
Asleep, Restless	yes/no	Cumulative asleep and restless sleep duration	Asleep + Restless	min
Awake, Restless	yes/no	Cumulative awake and restless sleep duration	Awake + Restless	min
Asleep, Awake, Restless, Unknown	yes/no	Cumulative sleep duration	Sleep	min
Heart rate	bpm ^1^	Minimum heart rate	MinHR	bpm
Heart rate	bpm ^1^	Mean heart rate	MeanHR	bpm
Heart rate	bpm ^1^	Median heart rate	MedianHR	bpm
Heart rate	bpm ^1^	Maximum heart rate	MaxHR	bpm
Heart rate	bpm ^1^	Standard deviation of heart rate	SDHR	bpm

^1^ bpm denotes beats/min.

**Table 3:** Formulae, predictors, and outcome for conditional logistic regression.

Formula	Predictors Defining the Scenarios	VA Outcome
1	(a) behavior aggregate, (b) season, weekday status	event (yes/no)
2	(a) behavior aggregate, (b) season, day of week	event (yes/no)
3	(a) behavior aggregate, (b) season	event (yes/no)

**Table 4:** Participant information.

Participant ID	Gender	Age	VA Events ^1^	Fitbit Days ^2^	Device Type ^3^
1	Male	67	6	193	ICD
2	Male	61	0	966	Not specified
3	Male	41	0	120	Not specified
4	Male	55	1	960	ICD
5	Male	66	6	364	ICD
6	Male	67	0	79	Not specified
7	Male	28	1	65	ICD
8	Male	69	0	567	Not specified
9	Male	47	1	519	ICD
10	Male	61	0	261	Not specified
11	Male	59	5	60	ICD
12	Male	66	23	647	CRT-D
13	Male	58	5	357	CRT-D
14	Male	67	1	317	ICD
15	Male	56	6	332	ICD
16	Female	52	11	980	ICD
17	Female	61	0	99	Not specified
18	Male	47	20	326	ICD
19	Male	45	45	450	ICD
20	Male	67	127	801	ICD
21	Male	66	0	148	Not specified
22	Male	69	1	395	ICD
23	Male	38	0	98	Not specified
24	Male	59	0	136	Not specified
25	Male	51	3	842	ICD
26	Male	49	0	891	Not specified
27	Male	74	0	796	Not specified

^1^ Refers to the number of VA events recorded. ^2^ Refers to the number of days with Fitbit measurements. ^3^ Refers to the use of an implantable cardiac-defibrillator (ICD) or ICD with cardiac resynchronization therapy (CRT-D).

**Table 5:** Summary of results from the conditional logistic regression models: odd ratios for a unit increase in behavior.

Behavior	Separate Models			Combined Models			Result
	OR	*p*	Duration	OR	*p*	Duration	OR
Steps	0.987	<0.001	7	1.000	0.002	7–8	Inconclusive
Light	1.009	0.017	2–3	1.010	<0.001	4–5	OR > 1
Light + Fair	1.008	0.025	2	1.010	<0.001	5	OR > 1
Fair	0.001	<0.001	6	0.973	0.016	4–5, 8	OR < 1
Fair + Vig	0.260	<0.001	7	0.991	0.013	2–8	OR < 1
Vig	0.104	<0.001	7	0.988	0.024	2–6, 8	OR < 1
Asleep	-	-	-	-	-	-	Inconclusive
Awake	-	-	-	0.788	0.003	8	OR < 1
Restless	-	-	-	-	-	-	Inconclusive
Asleep + Awake	-	-	-	0.788	0.003	8	OR < 1
Awake + Restless	-	-	-	0.791	0.003	8	OR < 1
Asleep + Restless	-	-	-	-	-	-	Inconclusive
Sleep	0.001	<0.001	7	0.991	0.001	6–8	OR < 1
MinHR	1.107	0.046	1	1.119	<0.001	4–8	OR > 1
MeanHR	1.270	0.003	3	1.139	<0.001	8	OR > 1
MedianHR	1.244	0.016	3–4	1.140	<0.001	8	OR > 1
MaxHR	1.072	0.048	2	1.048	<0.001	4	OR > 1
SDHR	-	-	-	-	-	-	Inconclusive

Period durations (in weeks) where odd ratios were at least as extreme as those reported are included. Color red indicates OR > 1, green indicates OR < 1, and yellow indicates an inconclusive result. For all results, *p* < 0.05.

**Figure A1:**
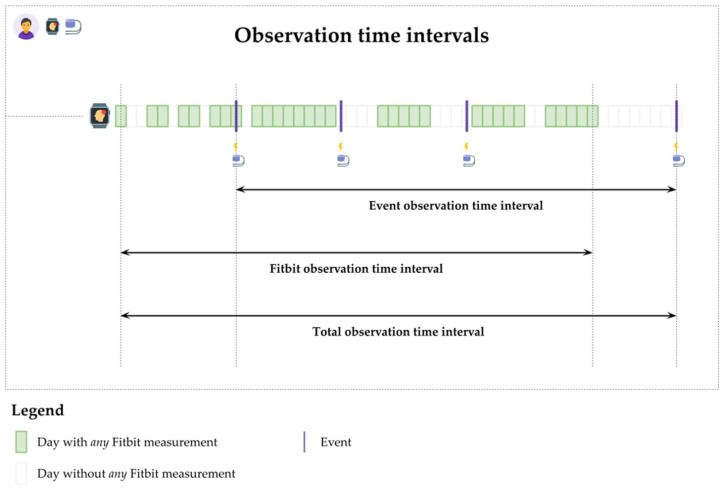
Observation time intervals. Fitbit-monitored days (green) and events (magenta) are depicted on a timeline. The first and last event days delimit the event observation time interval. The first and last Fitbit monitoring days delimit the Fitbit observation time interval. The first event or first Fitbit-monitored day (whichever occurs first) and the last event or last Fitbit-monitored day (whichever occurs last) delimit the total observation time interval.

**Figure A2:**
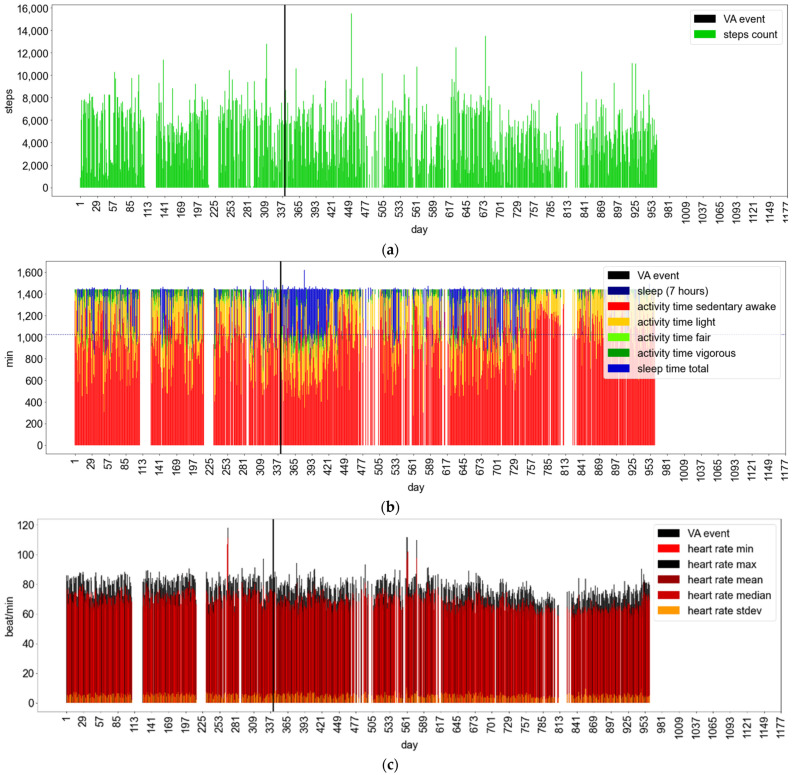
Daily life behaviors (bars) and VA events (strokes) observed in the Fitbit monitoring time interval for Patient 4; (**a**) steps; (**b**) physical activity—sedentary awake includes non-measured and measured-as-sedentary durations excluding measured sleep—and sleep duration; (**c**) heart rate.

**Figure A3:**
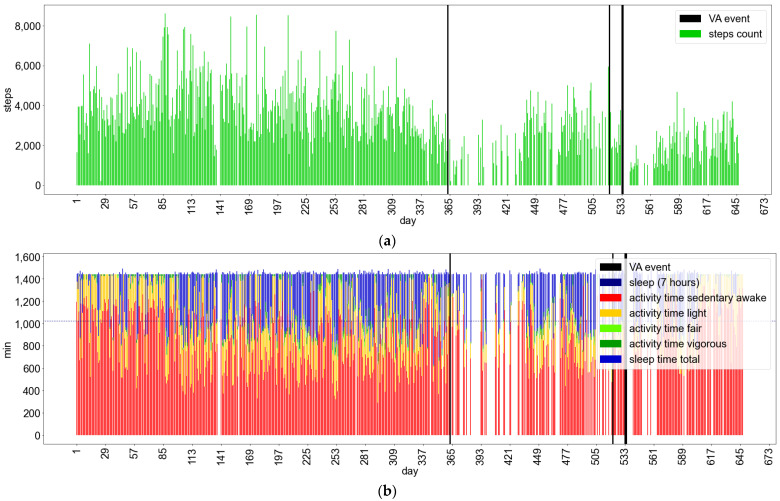

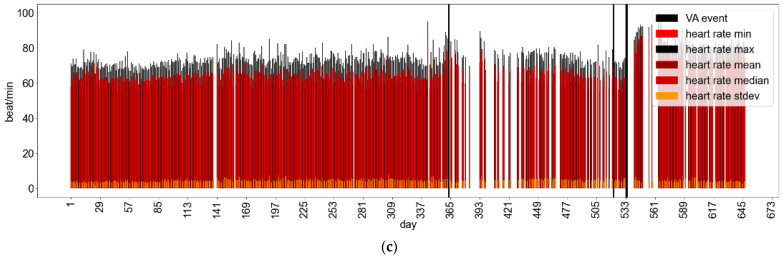
Daily life behaviors (bars) and VA events (strokes) observed in the Fitbit monitoring time interval for Patient 12; (**a**) steps; (**b**) physical activity—sedentary awake includes non-measured and measured-as-sedentary durations excluding measured sleep—and sleep duration; (**c**) heart rate.

**Figure A4:**
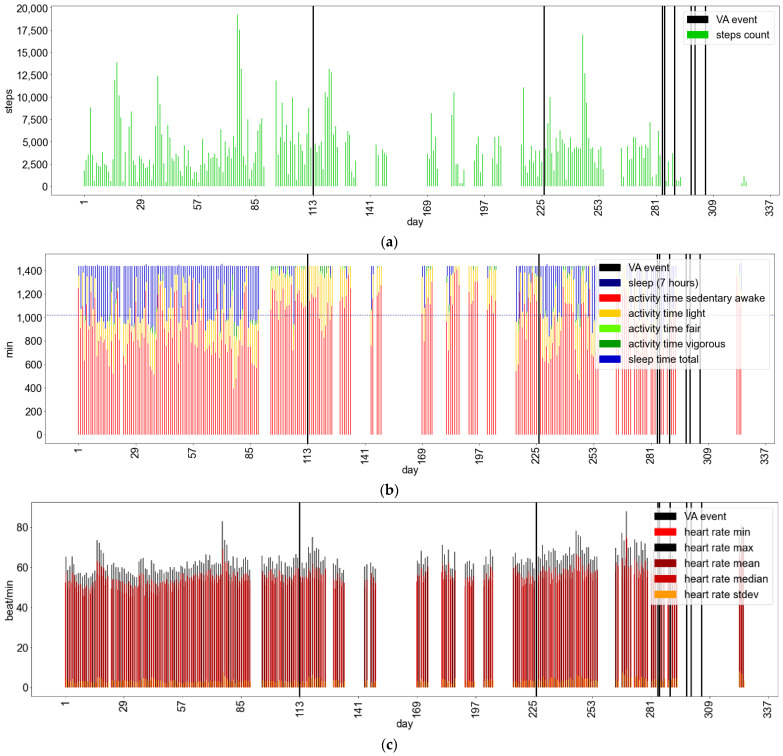
Daily life behaviors (bars) and VA events (strokes) observed in the Fitbit monitoring time interval for Patient 18; (**a**) steps; (**b**) physical activity—sedentary awake includes non-measured and measured-as-sedentary durations excluding measured sleep—and sleep duration; (**c**) heart rate.

## Data Availability

The data are not publicly available due to study Institutional Review Board (IRB) and the General Data Protection Regulation (GDPR).

## Supplementary Materials

The following supporting information can be downloaded at: https://www.mdpi.com/article/10.3390/jpm12060942/s1. The supplementary files contain a Data Format Example (Listing S1) and the Data Analysis Code (Listing S2).

## Abbreviations

The following abbreviations are used in this manuscript:

Abbreviation	Term
Active	Cumulative light, fair, and vigorous-intensity physical activity duration
Asleep	Duration of sleep in the asleep type
Asleep + Awake	Cumulative duration of sleep in the asleep and awake types
Asleep + Restless	Cumulative duration of sleep in the asleep and restless types
Awake	Duration of sleep of the awake type
Awake + Restless	Cumulative duration of sleep in the awake and restless types
CA	California
CRT	Cardiac resynchronization therapy defibrillator
CSV	Comma-separated value format
Fair	Fair-intensity physical activity duration
Fair + Vig	Cumulative fair and vigorous-intensity physical activity duration
FDA	United States Food and Drug Administration
HR	Heart rate
ICD	Implantable cardioverter-defibrillator
JSON	JavaScript object notation format
Light	Light-intensity physical activity duration
Light + Fair	Cumulative light and fair-intensity physical activity duration
MaxHR	Maximum heart rate
MeanHR	Mean heart rate
MedianHR	Median heart rate
MinHR	Minimum heart rate
OR	Odds ratio
Restless	Duration of sleep in the restless type
SCAUT	Self-, Collaborative- and AUTo-detection of signs and symptoms of deterioration
SD	Standard deviation
SDHR	Standard deviation of heart rate
Sed	Sedentary duration
Sed + Light	Cumulative sedentary and light-intensity physical activity duration
Sleep	Duration of sleep
Unknown	Duration of sleep of an unknown type
USA	United States of America
VA	Ventricular arrhythmia
Vig	Vigorous-intensity physical activity duration
VF-VT	Ventricular tachycardia into ventricular fibrillation
VT	Ventricular tachycardia
VT1	Ventricular tachycardia in Zone 1
VT2	Ventricular tachycardia in Zone 2

## Appendix A

### Appendix A.1. Fitbit as a Consumer Wearable Activity Tracker

From a space of consumer wearable activity trackers with over 200 models [22], we selected Fitbit for this study driven by three considerations: Fitbit embraces human factors for extended wear, reports daily life behavioral markers accurately, and allows researchers to collect data reliably. First, in past usability studies, participants found Fitbit “easy to use, useful, and acceptable” and the most usable compared to other wearables [23,24]. Second, Fitbit aims to motivate consumers to “reach health and fitness goals by tracking activity, exercise, sleep, weight, and more” [25]. Previous studies measured the accuracy of Fitbit consumer-friendly devices in reporting daily life behaviors of physical activity [26,27,28] and sleep [29,30]. Furthermore, Fitbit was selected for Digital Health software pre-certification by the US FDA [55]. Third, Fitbit reliably exposes behavioral markers for physical activity, sleep, and heart rate multiple times per day (sufficient for longitudinal studies) in the JSON and CSV formats, easy to read by both humans and machines. We selected the Fitbit Alta HR wearable for our study, a lightweight activity tracker that can monitor physical activity, sleep, and heart rate throughout the day.

### Appendix A.2. Data Quality Validation

We evaluated the quality of our data under three types of scenarios; for each the data is called ‘valid’ if its quality is above the threshold, and ‘invalid’ otherwise. The main results of the paper are based on the third scenario type for days and all scenarios for weeks and periods. The remainder of scenario types for days are elaborated on below.

A day was deemed valid if it did not include a VA event and:

- Scenario type 1: had a minimum of 18, 21, or 23 h of physical activity or sedentarism (three separate scenarios).
- Scenario type 2: had a minimum of 15, 30, or 45 min of physical activity (three separate scenarios).
- Scenario type 3: had a minimum of one, two, four, or eight hours of physical activity between 8 a.m. and 8 p.m. (four separate scenarios).

The scenario types permitted missing data for device battery charging and handling (1), the presence of at least some physical activity (2), and extended sedentary behaviors expected from older patients during the day (3). Contrary, the scenario types aimed at reducing the impact of missing measurements by modeling the day as a period of 24 h [45].

A week was deemed valid if it did not include a VA event and had at least four, six, or seven valid days (three separate scenarios). These thresholds were meant to ensure enough days during the week while allowing for a few days without monitoring. These thresholds are concordant with previous studies using Fitbit consumer wearables [31].

A 1–8-week period was deemed valid if it did not include a VA event and had at least 50%, 75%, or 100% valid weeks (three separate scenarios). We chose at least one week for the analysis to monitor data representative of daily life since Fitbit’s accuracy of the active minutes improves from one day to seven days [26].

## Appendix B

### Appendix B.1. Descriptive Analysis

#### Appendix B.1.1. Data Quality

This Appendix reports several time intervals measured in days, derived as follows:

- Event observation time interval: the difference between the patient’s earliest and latest days with VA events.
- Fitbit observation time interval: the difference between the patient’s earliest and latest days with any Fitbit monitoring.
- Total observation interval: the difference between the patient’s earliest and latest days with either VA event, Fitbit monitoring, or both.

An example is visible in Figure A1.

Table A1 illustrates the VA events by type. Table A2 shows the monitoring days for the patients.

**Table A1:** VA events by type and monitoring days (16 patients). The remaining 11 patients (up to 27) did not have VA events of the types included in this analysis.

Patient ID	Device Type	Events of Type					Event Observation Time Interval (Days)
		VT	VT1	VT2	VF-VT	Total	
1	ICD	0	6	0	0	6	4
4	ICD	1	0	0	0	1	1
5	ICD	1	0	0	5	6	419
7	ICD	0	0	0	1	1	1
9	ICD	0	0	0	1	1	1
11	ICD	0	2	0	3	5	1019
12	CRT-D	5	12	0	6	23	1034
13	CRT-D	2	0	0	3	5	642
14	ICD	0	0	0	1	1	1
15	ICD	0	0	0	6	6	2714
16	ICD	0	11	0	0	11	809
18	ICD	9	6	0	5	20	972
19	ICD	3	42	0	0	45	1718
20	ICD	35	90	0	2	127	869
22	ICD	0	0	0	1	1	1
25	ICD	0	3	0	0	3	829
	Sum	56	172	0	34	262	11,034
	Minimum	0	0	0	0	1	1
	Quartile 1	0	0	0	0	1	1
	Quartile 2	0	1	0	1	5.5	725.5
	Quartile 3	2.3	7.3	0	3.5	13.3	983.8
	Maximum	35	90	0	6	127	2714
	Mean	3.5	10.8	0	2.13	16.4	689.6
	SD	8.8	23.6	0	2.25	31.7	750.3

**Table A2:** Monitoring days for events, Fitbit, and overall (27 patients).

Patient ID	Event Observation Time Interval (Days)	Fitbit Observation Time Interval (Days)	Total Observation Time Interval (Days)
1	4	193	330
2	0	966	966
3	0	120	120
4	1	960	960
5	419	364	1331
6	0	79	79
7	1	65	3428
8	0	567	567
9	1	519	519
10	0	261	261
11	1019	60	1019
12	1034	647	1147
13	642	357	1725
14	1	317	449
15	2714	332	3352
16	809	980	1092
17	0	99	99
18	972	326	993
19	1718	450	1718
20	869	801	1079
21	0	148	148
22	1	395	1739
23	0	98	98
24	0	136	136
25	829	842	1723
26	0	891	891
27	0	796	796
Sum	11,034	11,769	26,765
Minimum	0	60	79
Quartile 1	0	142	295.5
Quartile 2	1	357	960.0
Quartile 3	819	721.5	1239
Maximum	2714	980	3428
Mean	408.7	435.9	991.3
SD	666.4	316.3	880.9

Table A3, Table A4 and Table A5 depict the Fitbit valid days for the three scenario types for days. For scenario type one (Table A3), the thresholds did not change the numbers of valid days due to the reporting of sedentary duration for any non-physically active duration. For scenario types two and three, increasingly strict thresholds diminished the number of valid days for some patients (Table A4 and Table A5).

**Table A3:** Valid Fitbit days for day validation scenario type 1 (27 patients).

Patient ID	Min. 18 h	Min. 21 h	Min. 23 h
	Valid Days	Valid Days	Valid Days
1	122	122	122
2	0	0	0
3	0	0	0
4	819	819	819
5	57	57	57
6	0	0	0
7	37	37	37
8	0	0	0
9	185	185	185
10	0	0	0
11	45	45	45
12	543	543	543
13	105	105	105
14	306	306	306
15	5	5	5
16	890	890	890
17	0	0	0
18	218	218	218
19	263	263	263
20	167	167	167
21	0	0	0
22	329	329	329
23	0	0	0
24	0	0	0
25	51	51	51
26	0	0	0
27	0	0	0
Sum	4142	4142	4142
Minimum	0	0	0
Quartile 1	0	0	0
Quartile 2	45	45	45
Quartile 3	201.5	201.5	201.5
Maximum	890	890	890
Mean	153.4	153.4	153.4
SD	243.2	243.2	243.2

**Table A4:** Valid Fitbit days for day validation scenario type 2 (27 patients).

Patient ID	Min. 15 min	Min. 30 min	Min. 45 min
	Valid Days	Valid Days	Valid Days
1	122	119	116
2	866	866	865
3	50	50	50
4	819	805	796
5	57	53	51
6	53	52	52
7	37	35	35
8	448	448	446
9	185	182	176
10	211	211	210
11	45	44	42
12	543	543	540
13	105	104	102
14	306	303	303
15	5	5	5
16	890	890	890
17	96	96	96
18	218	217	212
19	263	254	243
20	167	165	161
21	145	145	145
22	329	328	328
23	88	88	86
24	132	132	131
25	51	50	47
26	794	793	788
27	652	650	642
Sum	7677	7628	7558
Minimum	5	5	5
Quartile 1	72.5	70.5	69
Quartile 2	167	165	161
Quartile 3	388.5	388	387
Maximum	890	890	890
Mean	284.3	282.5	279.9
SD	284.3	283.6	282.7

**Table A5:** Valid Fitbit days for day validation scenario type 3 (27 patients).

Patient ID	Min. 1 h	Min. 2 h	Min. 4 h	Min. 8 h
	Valid Days	Valid Days	Valid Days	Valid Days
1	115	112	96	13
2	864	843	468	0
3	48	47	31	0
4	787	740	531	72
5	50	44	33	4
6	47	44	22	0
7	32	31	23	10
8	442	425	286	0
9	166	157	148	60
10	204	170	28	0
11	42	36	28	1
12	537	505	278	2
13	101	92	33	0
14	303	302	250	5
15	4	4	3	0
16	890	890	883	606
17	96	89	15	0
18	207	176	52	2
19	238	222	180	29
20	159	152	135	27
21	143	139	54	0
22	327	324	306	128
23	80	59	3	0
24	128	74	5	0
25	46	44	39	1
26	769	645	148	0
27	611	407	8	0
Sum	7436	6773	4086	960
Minimum	4	4	3	0
Quartile 1	65	53	25.5	0
Quartile 2	159	152	52	1
Quartile 3	384.5	365.5	215	11.5
Maximum	890	890	883	606
Mean	275.4	250.9	151.3	35.6
SD	280.0	262.3	205.2	117.7

#### Appendix B.1.2. Behavioral Markers

Table A6, Table A7 and Table A8 illustrate the mean Fitbit behavioral markers for physical activity, sleep, and heart rate, respectively.

**Table A6:** Mean Fitbit steps and durations of physical activity intensities over the entire Fitbit observation time (27 patients).

Patient ID	Steps	Sed	Sed + Light	Light	Light + Fair	Fair	Fair + Vig	Vig	Active
1	8178.3	1060.4	1356.7	296.3	306.1	32.4	88.3	81.6	365.0
2	6677.4	828.1	1364.0	537.1	538.8	21.0	59.8	58.3	577.6
3	14,322.4	746.6	1136.4	390.0	457.2	88.5	223.8	151.2	597.2
4	4743.4	952.8	1243.3	292.7	307.4	32.3	73.9	56.4	351.8
5	6641.7	1094.7	1370.3	275.8	281.1	23.1	60.7	57.6	319.5
6	7019.0	968.8	1239.3	270.8	379.7	122.5	193.0	81.5	456.7
7	10,293.7	909.1	1373.3	464.6	465.4	15.0	70.6	72.4	510.4
8	16,683.7	563.8	947.1	444.3	628.3	198.6	339.0	148.0	769.7
9	6269.9	737.3	1171.1	437.0	446.5	34.4	88.1	76.7	510.3
10	4256.0	775.0	1104.1	330.6	339.2	31.0	58.9	47.9	371.7
11	4402.0	870.7	1212.4	348.3	349.0	15.0	30.0	33.0	356.3
12	3377.2	851.3	1118.7	275.1	282.3	23.6	45.8	38.1	310.4
13	2879.0	1085.9	1313.4	228.0	228.1	15.0	21.0	20.0	231.0
14	9990.4	942.5	1222.0	280.6	294.7	31.5	81.6	67.2	356.4
15	5297.6	1018.4	1351.4	333.0	333.0	0.0	15.0	15.0	336.0
16	14,794.4	762.7	1317.4	554.8	582.4	40.1	121.2	93.7	672.2
17	8528.1	938.3	1372.3	434.1	440.0	21.1	67.8	62.5	494.1
18	4347.6	969.4	1195.7	227.2	232.5	27.2	42.2	33.0	246.5
19	5126.9	794.0	1111.8	321.2	325.6	28.2	41.6	34.7	342.0
20	5763.7	943.0	1368.3	425.5	426.5	20.0	39.3	38.5	443.2
21	7978.4	810.3	1266.1	459.2	470.4	24.2	52.3	41.6	507.9
22	11,148.7	945.2	1296.2	351.1	392.0	53.1	136.8	96.5	483.7
23	6012.7	1058.1	1351.7	294.4	320.1	52.5	79.4	48.9	354.4
24	9588.3	1037.7	1325.3	287.6	347.3	69.7	110.0	51.1	391.8
25	8115.4	1064.1	1391.4	327.4	330.3	25.0	41.5	37.1	355.0
26	9570.8	962.3	1344.3	382.2	407.9	52.3	83.7	55.4	455.4
27	5021.6	1070.5	1327.9	257.8	273.3	31.3	55.9	39.5	302.6
Minimum	2879.0	563.8	947.1	227.2	228.1	0.0	15.0	15.0	231.0
Quartile 1	5074.3	819.2	1204.1	284.1	306.8	22.1	44.0	38.3	346.9
Quartile 2	6677.4	943.0	1313.4	330.6	347.3	31.0	67.8	55.4	371.7
Quartile 3	9579.6	1028.1	1354.2	429.8	443.3	46.2	88.2	74.6	501.0
Maximum	166,83.7	1094.7	1391.4	554.8	628.3	198.6	339.0	151.2	769.7
Mean	7667.7	917.1	1266.4	352.8	377.2	43.4	86.0	60.6	424.8
SD	3521.6	132.0	111.2	89.8	101.6	40.1	69.6	33.0	127.2

**Table A7:** Mean Fitbit sleep duration over the entire Fitbit observation time (27 patients).

Patient ID	Asleep	Awake	Restless	Asleep + Awake	Awake + Restless	Asleep + Restless	Sleep
1	276.3	9.0	19.2	280.8	24.6	292.3	296.8
2	186.4	6.6	10.8	192.3	16.5	196.7	199.8
3	294.7	3.0	10.5	295.8	11.5	305.2	306.2
4	290.6	4.7	13.2	292.4	17.1	302.0	303.1
5	152.6	4.0	9.1	155.7	12.2	161.7	164.8
6	124.0	4.0	10.0	125.3	11.6	132.3	133.7
7	180.0	4.7	10.0	183.5	13.5	190.0	193.5
8	245.8	5.6	11.0	250.2	15.4	256.7	261.0
9	264.4	4.5	20.9	268.2	24.6	285.1	289.4
10	420.9	8.0	21.2	426.6	27.0	441.9	447.6
11	231.1	9.0	21.7	238.9	29.7	252.2	260.1
12	370.3	11.1	35.5	380.8	46.2	405.2	414.9
13	171.0	4.0	11.4	172.4	12.9	181.4	182.8
14	206.4	4.9	9.5	208.6	11.8	214.4	216.6
15	102.3	1.0	4.5	102.5	4.8	106.8	107.0
16	109.5	2.6	9.9	110.9	11.4	119.2	119.1
17	78.5	1.7	4.8	80.0	6.3	83.3	82.0
18	288.6	2.5	19.7	290.0	21.1	308.3	309.7
19	353.9	5.8	15.0	357.7	18.9	368.4	372.2
20	112.4	2.3	9.1	113.2	9.9	121.1	121.1
21	113.8	4.0	15.3	115.9	17.3	128.6	130.6
22	159.3	3.6	10.2	161.8	12.6	169.5	166.9
23	130.7	2.3	8.1	131.8	9.2	138.8	139.9
24	127.0	2.3	5.4	127.7	6.1	132.4	126.5
25	101.3	2.5	7.4	101.8	8.1	106.5	107.0
26	138.1	2.3	10.7	138.9	11.7	148.1	146.8
27	214.1	5.7	12.7	216.6	15.4	225.3	226.1
Minimum	78.5	1.0	4.5	80.0	4.8	83.3	82.0
Quartile 1	125.5	2.5	9.3	126.5	11.5	132.4	132.2
Quartile 2	180.0	4.0	10.7	183.5	12.9	190.0	193.5
Quartile 3	270.4	5.7	15.2	274.5	18.1	288.7	293.1
Maximum	420.9	11.1	35.5	426.6	46.2	441.9	447.6
Mean	201.6	4.5	12.8	204.5	15.8	213.8	215.8
SD	92.4	2.5	6.7	94.0	8.8	97.5	99.6

**Table A8:** Mean Fitbit heart rate over the entire Fitbit observation time (27 patients).

Patient ID	MinHR	MeanHR	MedianHR	MaxHR	SDHR
1	45.1	57.2	50.9	115.3	4.2
2	62.3	94.0	94.5	134.8	3.7
3	49.9	73.7	69.1	129.2	5.8
4	51.6	69.1	65.8	125.0	4.8
5	46.7	65.0	61.1	116.7	5.4
6	43.5	65.4	58.6	123.5	5.8
7	56.4	85.5	84.7	135.1	4.1
8	54.8	84.0	81.1	148.1	4.7
9	58.2	81.1	78.2	127.3	4.2
10	43.3	61.8	58.7	122.5	3.8
11	48.0	63.6	62.5	102.9	2.8
12	50.9	67.1	63.8	120.0	4.2
13	53.0	63.1	60.4	111.4	3.6
14	39.6	54.7	51.7	115.8	3.9
15	45.2	63.6	63.0	102.0	2.9
16	53.2	75.6	72.1	152.5	5.1
17	48.4	69.4	65.7	126.9	4.5
18	43.5	57.0	55.4	111.1	3.8
19	52.7	65.8	61.3	114.0	3.5
20	68.0	82.9	81.2	118.7	2.6
21	60.3	77.6	75.7	131.0	3.2
22	49.7	69.2	63.4	132.0	4.8
23	47.7	67.5	63.8	136.8	4.0
24	39.5	55.6	50.4	144.8	6.1
25	47.5	63.7	61.3	116.6	4.1
26	49.9	71.2	69.1	137.0	3.9
27	49.5	65.6	63.7	120.8	3.8
Minimum	39.5	54.7	50.4	102.0	2.6
Quartile 1	46.0	63.6	60.8	116.2	3.8
Quartile 2	49.7	67.1	63.7	123.5	4.1
Quartile 3	53.1	74.7	70.6	133.4	4.8
Maximum	68.0	94.0	94.5	152.5	6.1
Mean	50.3	69.3	66.2	124.9	4.2
SD	6.7	9.8	10.7	12.7	0.9

#### Appendix B.1.3. Fitbit Wear and VA Events: Examples of Patient Behavioral Patterns

Patient 4 (male, 55-year-old, device type ICD) were monitored over a period of 960 days (Table A2). He experienced one VA event of type VT (Table A1). He is a compliant user with 960 Fitbit days (Table A2) from which up to 819 are valid Fitbit days (Table A3, Table A4 and Table A5). Patient 4 belonged to the lower half of physical activity levels of our sample (Table A6), had sleep monitored with higher compliance than average (Table A7), and did not experience extreme Fitbit-reported heart rates (Table A8). He self-reported that he used the Fitbit device to learn that *“heart disease increases one’s average resting heart rate” and that “activity improves one’s average heart rate”* [20]. He set exercise goals for walking and was keen on monitoring his heart rate while resting; reporting that *“it makes a difference whether [his] heart rate is 70 or 60 beats per minute when resting”* [20].

The Fitbit activity tracker for Patient 4 recorded only a few days of sleep before a VA. The daily heart rate kept increasing for two weeks before the event. Then, right after the event and for three months, Fitbit recorded daily sleep durations almost every day. Then, Fitbit recorded sleep more sporadically, as before the event. Figure A2 shows the measurement period in extenso for Patient 4.

Upon experiencing the VA event, Patient 4 may have decided to extend the wear of his activity tracker during the awake sedentary time (as self-reported) and during the sleeping time to monitor his heart rate in expectation of a future VA event. The absence of a second VA event within three months may have contributed to his effort’s decay.

Patient 12 (male, 66-year-old, device type CRT-D) was monitored over a period of 1147 days (Table A2). He experienced 23 VA events, five of type VT, 12 of type VT1, and six of type VF-VT (Table A1) over a period of 1034 days (Table A2). He is also a compliant Fitbit user with 647 days (Table A2) from which up to 543 valid days (Table A3, Table A4 and Table A5). His physical activity was below average (Table A6), his sleep was above average (Table A7), and his Fitbit-reported heart rate was not extreme (Table A8). However, he suffered from disease-related anxiety, was very alert to bodily signs, and had difficulty sleeping: *“as soon as there is even a little thing in these zones* [in his chest region], *and I would even say just one, like a sprain, I get nervous”* [20]. He self-reported that Fitbit helped him feel safe: *“now I get certainty. Is something wrong or not”* [20], but he also evoked doubts about its usefulness after he and his wife saw it overestimate sleep: *“we could not make the numbers fit because I had been awake a lot”* [20].

This patient wore the device compliantly for one year, measuring physical activity and sleep. The Fitbit tracker measured sleep for about nine months before the initial VA event. Right before the event, the heart rate increased for about one week. For three months after the initial VA event, the patient wore the tracker with interruptions. A series of other VA events led to a similar interruption of wear. His steps decreased in the time intervals following VA events. Figure A3 shows the measurement period in extenso for Patient 12.

After experiencing the first VA event, the compliance of Patient 12 decreased with interrupted wear time lasting several weeks and he performed fewer steps, potentially recovering from the event. However, before the second event, reduced compliance was observed again, with partial Fitbit wear during the day. The change in wear indicates that his behaviors or physical state may have changed (e.g., he started a new type of activity that may have contributed to the VA event).

Patient 18 (male, 47-year-old, device type ICD) was monitored over a period of 993 days (Table A2). He experienced 20 VA events, nine of type VT, six of type VT1, and five of type VF-VT (Table A1) over a period of 972 days (Table A2). He wore the Fitbit for 326 days (Table A2) of which up to 218 were valid (Table A3, Table A4 and Table A5). He was less active than average (Table A6), measured more sleep than average (Table A7), and had a lower heart rate than average (Table A8). He only evoked doubts about using the Fitbit device, indicating that *“sensing is more useful than activity data”* [20], but was otherwise compliant in wearing it.

We first observed a decrease in wear after a series of VA events for Patient 18. He was compliant in the first three months when both physical activity and sleep were measured. Then, wear continued for one month, but almost without sleep. During this period, an event occurred. After the event, Fitbit recorded one month of similar compliance and three months of reduced compliance. A second event was followed by increased compliance for less than two months. Finally, the third series of events along 15–20 days coincided with the cessation of compliant wear. Figure A4 shows the measurement period in extenso for Patient 18.

Before the event, the wearing pattern changed, indicating that Patient 18 may have begun a new activity or entered a physical state that reduced wear at night or natural sleep and culminated in a VA. One month after the event, the wearing pattern continued, indicating that the patient may have continued daily life without changing his activity or state that contributed to the first event. Towards the end of the monitoring period, the patient may have experienced too many events or lost faith in using the device for self-monitoring.

### Appendix B.2. Inferential Analysis

Table A9, Table A10 and Table A11 illustrate the results across all conditional logistic regression models for the three validation scenario types for days. Table A12 summarizes the results across all validation scenario types for days, in agreement with the results reported in the main paper. Additionally, Table A13 presents the results by period duration in the third validation scenario.

**Table A9:** Results across all conditional logistic regression models in validation scenario type 1.

Behavior	Separate		Combined		Result
	OR	*p*	OR	*p*	OR
Steps	-	-	1.000	<0.001	Inconclusive
Sed ^1^	-	-	1.004	0.003	OR > 1
Sed + Light ^1^	-	-	1.004	0.003	OR > 1
Light	1.013	0.008	1.004	0.003	OR > 1
Light + Fair	1.012	0.012	1.008	<0.001	OR > 1
Fair	-	-	-	-	Inconclusive
Fair + Vig	-	-	-	-	Inconclusive
Vig	-	-	-	-	Inconclusive
Asleep	-	-	-	-	Inconclusive
Awake	-	-	0.800	0.001	OR < 1
Restless	-	-	-	-	Inconclusive
Asleep + Awake	-	-	0.800	0.001	OR < 1
Awake + Restless	-	-	0.802	0.001	OR < 1
Asleep + Restless	-	-	-	-	Inconclusive
Sleep	0.014	0.023	0.991	<0.001	OR < 1
MinHR	2.307	0.003	1.119	<0.001	OR > 1
MeanHR	1.591	0.002	1.126	<0.001	OR > 1
MedianHR	1.605	0.010	1.129	<0.001	OR > 1
MaxHR	33.909	<0.001	1.045	<0.001	OR > 1
SDHR	-	-	-	-	Inconclusive

^1^ Durations including sedentary may include non-wear time—discarded from the main results. Color red depicts OR > 1, green depicts OR < 1, and yellow depicts an inconclusive result. All results *p* < 0.05.

**Table A10:** Results across all conditional logistic regression models in validation scenario type 2.

Behavior	Separate		Combined		Result
	OR	*p*	OR	*p*	OR
Steps	-	-	1.000	<0.001	Inconclusive
Sed ^1^	-	-	1.004	0.007	OR > 1
Sed + Light ^1^	-	-	1.004	0.006	OR > 1
Light	1.013	0.014	1.009	<0.001	OR > 1
Light + Fair	1.012	0.019	1.008	<0.001	OR > 1
Fair	-	-	-	-	Inconclusive
Fair + Vig	-	-	-	-	Inconclusive
Vig	-	-	-	-	Inconclusive
Asleep	-	-	-	-	Inconclusive
Awake	-	-	0.815	0.003	OR < 1
Restless	-	-	-	-	Inconclusive
Asleep + Awake	-	-	0.802	0.001	OR < 1
Awake + Restless	-	-	0.817	0.001	OR < 1
Asleep + Restless	-	-	-	-	Inconclusive
Sleep	0.014	0.023	0.991	<0.001	OR < 1
MinHR	2.307	0.003	1.119	<0.001	OR > 1
MeanHR	1.591	0.002	1.126	<0.001	OR > 1
MedianHR	1.605	0.010	1.129	<0.001	OR > 1
MaxHR	33.909	<0.001	1.046	<0.001	OR > 1
SDHR	-	-	-	-	Inconclusive

^1^ Durations including sedentary may include non-wear time—discarded from the main results. Color red depicts OR > 1, green depicts OR < 1, and yellow depicts an inconclusive result. All results *p* < 0.05.

**Table A11:** Results across all conditional logistic regression models in validation scenario type 3.

Behavior	Separate		Combined		Result
	OR	*p*	OR	*p*	OR
Steps	0.987	<0.001	1.000	0.002	Inconclusive
Sed ^1^	0.065	<0.001	1.004	0.007	Inconclusive
Sed + Light ^1^	0.063	0.012	1.004	0.006	Inconclusive
Light	1.009	0.017	1.010	<0.001	OR > 1
Light + Fair	1.008	0.025	1.010	<0.001	OR > 1
Fair	0.001	<0.001	0.973	0.016	OR < 1
Fair + Vig	0.260	<0.001	0.991	0.013	OR < 1
Vig	0.104	<0.001	0.988	0.024	OR < 1
Asleep	-	-	-	-	Inconclusive
Awake	-	-	0.788	0.003	OR < 1
Restless	-	-	-	-	Inconclusive
Asleep + Awake	-	-	0.788	0.003	OR < 1
Awake + Restless	-	-	0.791	0.003	OR < 1
Asleep + Restless	-	-	-	-	Inconclusive
Sleep	0.001	<0.001	0.991	0.001	OR < 1
MinHR	1.107	0.046	1.119	<0.001	OR > 1
MeanHR	1.270	0.003	1.139	<0.001	OR > 1
MedianHR	1.244	0.016	1.140	<0.001	OR > 1
MaxHR	1.072	0.048	1.048	<0.001	OR > 1
SDHR	-	-	-	-	Inconclusive

^1^ Durations including sedentary may include non-wear time—discarded from the main results. Color red depicts OR > 1, green depicts OR < 1, and yellow depicts an inconclusive result. All results *p* < 0.05.

**Table A12:** Results across all conditional logistic regression models in all validation scenario types.

Behavior	Scenario Type 1	Scenario Type 2	Scenario Type 3	Result
	OR	OR	OR	OR
Steps	Inconclusive	Inconclusive	Inconclusive	Inconclusive
Sed ^1^	OR > 1	OR > 1	Inconclusive	OR > 1
Sed + Light ^1^	OR > 1	OR > 1	Inconclusive	OR > 1
Light	OR > 1	OR > 1	OR > 1	OR > 1
Light + Fair	OR > 1	OR > 1	OR > 1	OR > 1
Fair	Inconclusive	Inconclusive	OR < 1	OR < 1
Fair + Vig	Inconclusive	Inconclusive	OR < 1	OR < 1
Vig	Inconclusive	Inconclusive	OR < 1	OR < 1
Asleep	Inconclusive	Inconclusive	Inconclusive	Inconclusive
Awake	OR < 1	OR < 1	OR < 1	OR < 1
Restless	Inconclusive	Inconclusive	Inconclusive	Inconclusive
Asleep + Awake	OR < 1	OR < 1	OR < 1	OR < 1
Awake + Restless	OR < 1	OR < 1	OR < 1	OR < 1
Asleep + Restless	Inconclusive	Inconclusive	Inconclusive	Inconclusive
Sleep	OR < 1	OR < 1	OR < 1	OR < 1
MeanHR	OR > 1	OR > 1	OR > 1	OR > 1
MedianHR	OR > 1	OR > 1	OR > 1	OR > 1
MinHR	OR > 1	OR > 1	OR > 1	OR > 1
MaxHR	OR > 1	OR > 1	OR > 1	OR > 1
SDHR	Inconclusive	Inconclusive	Inconclusive	Inconclusive

^1^ Durations including sedentary may include non-wear time—discarded from the main results. Color red depicts OR > 1, green depicts OR < 1, and yellow depicts an inconclusive result. All results *p* < 0.05.

**Table A13:** Results by period duration across all conditional logistic regression models in validation scenario type 3.

Behavior	Duration	Separate			Combined		
		OR	*p*	Model	OR	*p*	Model
Steps	4 weeks	-	-	-	1.000	0.036	2
	5 weeks	-	-	-	1.000	0.019	2
	6 weeks	0.997	<0.001	2	1.000	0.008	2
	7 weeks	0.987	<0.001	2	1.000	0.002	2
	8 weeks	-	-	-	1.000	0.002	2
Light	1 weeks	1.006	0.033	2	1.006	0.033	2
	2 weeks	1.009	0.017	3	1.008	0.001	2
	3 weeks	1.013	0.038	3	1.009	<0.001	3
	4 weeks	-	-	-	1.010	<0.001	1
	5 weeks	-	-	-	1.010	<0.001	1
	6 weeks	-	-	-	1.008	<0.001	3
	7 weeks	-	-	-	1.009	<0.001	3
	8 weeks	-	-	-	1.009	<0.001	3
Light + Fair	1 weeks	1.006	0.048	2	1.006	0.048	2
	2 weeks	1.008	0.025	2	1.008	0.002	2
	3 weeks	-	-	-	1.008	<0.001	2
	4 weeks	-	-	-	1.009	<0.001	1
	5 weeks	-	-	-	1.010	<0.001	1
	6 weeks	-	-	-	1.008	<0.001	3
	7 weeks	1.076	0.015	1	1.008	<0.001	3
	8 weeks	-	-	-	1.008	<0.001	3
Fair	4 weeks	-	-	-	0.972	0.033	1
	5 weeks	-	-	-	0.972	0.022	1
	6 weeks	0.001	<0.001	2	0.974	0.021	1
	7 weeks	0.028	<0.001	2	0.975	0.021	1
	8 weeks	-	-	-	0.973	0.016	1
Fair + Vig	2 weeks	-	-	-	0.988	0.042	1
	3 weeks	-	-	-	0.989	0.029	1
	4 weeks	-	-	-	0.990	0.021	1
	5 weeks	-	-	-	0.990	0.015	1
	6 weeks	0.819	<0.001	2	0.991	0.017	1
	7 weeks	0.260	<0.001	2	0.991	0.019	1
	8 weeks	-	-	-	0.991	0.013	1
Vig	2 weeks	-	-	-	0.984	0.044	3
	3 weeks	-	-	-	0.986	0.036	3
	4 weeks	-	-	-	0.987	0.029	1
	5 weeks	-	-	-	0.987	0.025	1
	6 weeks	-	-	-	0.988	0.028	1
	7 weeks	0.104	<0.001	2	0.989	0.034	1
	8 weeks	-	-	-	0.988	0.024	1
Awake	3 weeks	-	-	-	0.831	0.031	2
	4 weeks	-	-	-	0.823	0.019	3
	5 weeks	-	-	-	0.813	0.011	3
	6 weeks	-	-	-	0.803	0.007	3
	7 weeks	-	-	-	0.793	0.004	3
	8 weeks	-	-	-	0.788	0.003	3
Asleep + Awake	3 weeks	-	-	-	0.831	0.031	2
	4 weeks	-	-	-	0.823	0.019	3
	5 weeks	-	-	-	0.813	0.011	3
	6 weeks	-	-	-	0.803	0.007	3
	7 weeks	-	-	-	0.793	0.004	3
	8 weeks	-	-	-	0.788	0.003	3
Awake + Restless	3 weeks	-	-	-	0.834	0.031	2
	4 weeks	-	-	-	0.826	0.019	3
	5 weeks	-	-	-	0.816	0.011	3
	6 weeks	-	-	-	0.806	0.007	3
	7 weeks	-	-	-	0.796	0.004	3
	8 weeks	-	-	-	0.791	0.003	3
Sleep	3 weeks	-	-	-	0.993	0.025	2
	4 weeks	-	-	-	0.993	0.013	1
	5 weeks	-	-	-	0.992	0.007	3
	6 weeks	-	-	-	0.991	0.004	3
	7 weeks	0.001	<0.001	2	0.991	0.002	3
	8 weeks	-	-	-	0.991	0.001	3
MinHR	1 weeks	1.107	0.046	2	1.107	0.046	2
	2 weeks	-	-	-	1.108	0.011	1
	3 weeks	-	-	-	1.114	0.003	1
	4 weeks	-	-	-	1.119	0.001	3
	5 weeks	-	-	-	1.119	<0.001	3
	6 weeks	-	-	-	1.118	<0.001	3
	7 weeks	-	-	-	1.116	<0.001	3
	8 weeks	-	-	-	1.116	<0.001	3
MeanHR	2 weeks	1.165	0.036	3	1.104	0.015	2
	3 weeks	1.270	0.031	2	1.122	0.002	2
	4 weeks	-	-	-	1.128	<0.001	3
	5 weeks	-	-	-	1.133	<0.001	3
	6 weeks	-	-	-	1.136	<0.001	3
	7 weeks	-	-	-	1.137	<0.001	3
	8 weeks	-	-	-	1.139	<0.001	3
MedianHR	2 weeks	1.171	0.016	3	1.111	0.003	1
	3 weeks	1.244	0.016	3	1.125	<0.001	1
	4 weeks	1.322	0.040	3	1.131	<0.001	3
	5 weeks	-	-	-	1.134	<0.001	3
	6 weeks	-	-	-	1.136	<0.001	3
	7 weeks	-	-	-	1.137	<0.001	3
	8 weeks	-	-	-	1.140	<0.001	3
MaxHR	2 weeks	1.072	0.048	2	1.040	0.006	2
	3 weeks	-	-	-	1.047	0.001	2
	4 weeks	-	-	-	1.048	<0.001	2
	5 weeks	-	-	-	1.038	<0.001	2
	6 weeks	-	-	-	1.041	<0.001	2
	7 weeks	-	-	-	1.044	<0.001	2
	8 weeks	-	-	-	1.045	<0.001	2

Color blue depicts the lowest P-value found, green depicts the corresponding OR < 1, red depicts the corresponding OR > 1, and yellow depicts the corresponding inconclusive OR. All results *p* < 0.05. Durations without statistically significant results were excluded.

## References

[1] Bardy G.H., Lee K.L., Mark D., Poole J.E., Packer D.L., Boineau R., Domanski M., Troutman C., Anderson J., Johnson G. (2005). Amiodarone or an Implantable Cardioverter–Defibrillator for Congestive Heart Failure. N. Engl. J. Med..

[2] Koller M.T., Schaer B., Wolbers M., Sticherling C., Bucher H.C., Osswald S. (2008). Death Without Prior Appropriate Implantable Car-dioverter-Defibrillator Therapy: A Competing Risk Study. Circulation.

[3] Crossley G.H., Boyle A., Vitense H., Chang Y., Mead R.H. (2011). The CONNECT (Clinical Evaluation of Remote Notification to Reduce Time to Clinical Decision) Trial: The Value of Wireless Remote Monitoring with Automatic Clinician Alerts. J. Am. Coll. Cardiol..

[4] Landolina M., Perego G.B., Lunati M., Curnis A., Guenzati G., Vicentini A., Parati G., Borghi G., Zanaboni P., Valsecchi S. (2012). Remote Monitoring Reduces Healthcare Use and Improves Quality of Care in Heart Failure Patients with Im-plantable Defibrillators. Circ. Am. Heart Assoc..

[5] Epstein A.E., DiMarco J.P., Ellenbogen K.A., Estes N.A.M., Freedman R.A., Gettes L.S., Gillinov A.M., Gregoratos G., Hammill S.C., Hayes D.L. (2013). 2012 ACCF/AHA/HRS focused update incor-porated into the ACCF/AHA/HRS 2008 guidelines for device-based therapy of cardiac rhythm abnormalities: A report of the American College of Cardiology Foundation/American Heart Association Task Force on Practice Guidelines and the Heart Rhythm Society. J. Am. Coll. Cardiol..

[6] Piwek L., Ellis D.A., Andrews S., Joinson A. (2016). The Rise of Consumer Health Wearables: Promises and Barriers. PLoS Med. Public Libr. Sci..

[7] Bayoumy K., Gaber M., Elshafeey A., Mhaimeed O., Dineen E.H., Marvel F.A., Martin S.S., Muse E.D., Turakhia M.P., Tarakji K.G. (2021). Smart wearable devices in cardiovascular care: Where we are and how to move forward. Nat. Rev. Cardiol..

[8] Yang C.-C., Hsu Y.-L. (2010). A Review of Accelerometry-Based Wearable Motion Detectors for Physical Activity Monitoring. Sensors.

[9] Hammond-Haley M., Allen C., Han J., Patterson T., Marber M., Redwood S. (2021). Utility of wearable physical activity monitors in cardiovascular disease: A systematic review of 11 464 patients and recommendations for optimal use. Eur. Heart J. Digit. Health.

[10] Perez M.V., Mahaffey K.W., Hedlin H., Rumsfeld J.S., Garcia A., Ferris T., Balasubramanian V., Russo A.M., Rajmane A., Cheung L. (2019). Large-Scale Assessment of a Smartwatch to Identify Atrial Fibrillation. N. Engl. J. Med..

[11] Wang L., Nielsen K., Goldberg J., Brown J.R., Rumsfeld J.S., Steinberg B.A., Zhang Y., Matheny M.E., Shah R.U. (2021). Association of Wearable Device Use with Pulse Rate and Health Care Use in Adults with Atrial Fibrillation. JAMA Netw. Open.

[12] Melin M., Hagerman I., Gonon A., Gustafsson T., Rullman E. (2016). Variability in Physical Activity Assessed with Accelerometer Is an Independent Predictor of Mortality in CHF Patients. PLoS ONE.

[13] Baril J.-F., Bromberg S., Moayedi Y., Taati B., Manlhiot C., Ross H.J., Cafazzo J. (2019). Use of Free-Living Step Count Monitoring for Heart Failure Functional Classification: Validation Study. JMIR Cardio.

[14] Evangelista L.S., Hamilton M.A., Fonarow G.C., Dracup K. (2010). Is Exercise Adherence Associated with Clinical Outcomes in Patients with Advanced Heart Failure?. Phys. Sportsmed..

[15] Tan K.H.M., Wong J., Bakrania K., Abdullahi Y., Harling L., Casula R., Rowlands A., Athanasiou T., Jarral O.A. (2018). Can activity monitors predict outcomes in patients with heart failure? A systematic review. Eur. Heart J. Qual. Care Clin. Outcomes.

[16] Vetrovsky T., Clark C., Bisi M.C., Siranec M., Linhart A., Tufano J.J., Duncan M., Belohlavek J. (2020). Advances in accelerometry for cardiovascular patients: A systematic review with practical recommendations. ESC Heart Fail..

[17] Andersen T.O., Nielsen K.D., Moll J., Svendsen J.H. (2019). Unpacking telemonitoring work: Workload and telephone calls to patients in implanted cardiac device care. Int. J. Med. Inform..

[18] Andersen T.O., Bansler J.P., Kensing F., Moll J., Mønsted T., Nielsen K.D., Nielsen O.W., Petersen H.H., Svendsen J.H. (2018). Aligning Concerns in Telecare: Three Concepts to Guide the Design of Patient-Centred E-Health. Comput. Support. Coop. Work.

[19] Andersen T.O., Langstrup H., Lomborg S. (2020). Experiences with Wearable Activity Data During Self-Care by Chronic Heart Pa-tients: Qualitative Study. J. Med. Internet Res..

[20] Lomborg S., Langstrup H., Andersen T.O. (2020). Interpretation as luxury: Heart patients living with data doubt, hope, and anxiety. Big Data Soc. SAGE Publ. Ltd..

[21] PLATFORM ISF Wearables: The Comprehensive List of Wearables on The Market|inKin. https://www.inkin.com/wearables/.

[22] McMahon S.K., Lewis B., Oakes M., Guan W., Wyman J.F., Rothman A.J. (2016). Older Adults’ Experiences Using a Commercially Available Monitor to Self-Track Their Physical Activity. JMIR mHealth uHealth.

[23] Steinert A., Haesner M., Steinhagen-Thiessen E. (2017). Activity-tracking devices for older adults: Comparison and preferences. Univers. Access Inf. Soc..

[24] Fitbit Official Site for Activity Trackers and More. https://www.fitbit.com.

[25] Ferguson T., Rowlands A.V., Olds T., Maher C. (2015). The validity of consumer-level, activity monitors in healthy adults worn in free-living conditions: A cross-sectional study. Int. J. Behav. Nutr. Phys. Act..

[26] Brewer W., Swanson B.T., Ortiz A. (2017). Validity of Fitbit’s active minutes as compared with a research-grade accelerometer and self-reported measures. BMJ Open Sport Exerc. Med..

[27] Kooiman T.J.M., Dontje M.L., Sprenger S.R., Krijnen W.P., van der Schans C.P., de Groot M. (2015). Reliability and validity of ten consumer activity trackers. BMC Sports Sci. Med. Rehabil..

[28] Keill A.K., An H.-S., Dinkel D.M., Lee J.-M. (2016). Validity of Wearable Fitness Trackers on Sleep Measure: 106 Board #4 June 1, 9. Med. Sci. Sports Exerc..

[29] Lee J.-M., Byun W., Keill A., Dinkel D., Seo Y. (2018). Comparison of Wearable Trackers’ Ability to Estimate Sleep. Int. J. Environ. Res. Public Health.

[30] U.S. Food and Drug Administration Digital Health Software Precertification (Pre-Cert) Program. FDA. https://www.fda.gov/medical-devices/digital-health-center-excellence/digital-health-software-precertification-pre-cert-program.

[31] Medtronic Medtronic CareLink® Network—Part of Cardiac Device Data Connectivity. https://www.medtronic.com/ca-en/healthcare-professionals/products/cardiac-rhythm/patient-management-carelink/mainspring-data-express.html.

[32] Fitbit Development: Web API. https://dev.fitbit.com/build/reference/web-api/.

[33] What are Active Zone Minutes or active minutes on my Fitbit device?. https://help.fitbit.com/articles/en_US/Help_article/1379.htm.

[34] Welcome to Python.org. Python.org. https://www.python.org/.

[35] Anaconda: Where Packages, Notebooks, Projects, and Environments Are Shared. https://anaconda.org/.

[36] R Core Team (2020). European Environment Agency. https://www.eea.europa.eu/data-and-maps/indicators/oxygen-consuming-substances-in-rivers/r-development-core-team-2006.

[37] RStudio (2022)—Open Source & Professional Software for Data Science Teams. https://www.rstudio.com/.

[38] Survival—A Package for Survival Analysis in R. 2020. https://cran.r-project.org/web/packages/survival/citation.html.

[39] Manea V., Wac K. (2020). Co-Calibrating Physical and Psychological Outcomes and Consumer Wearable Activity Outcomes in Older Adults: An Evaluation of the coQoL Method. J. Pers. Med..

[40] Low C.A., Dey A.K., Ferreira D., Kamarck T., Sun W., Bae S., Doryab A. (2017). Estimation of Symptom Severity during Chemotherapy from Passively Sensed Data: Exploratory Study. J. Med. Internet Res..

[41] Katzan I., Schuster A., Kinzy T. (2021). Physical Activity Monitoring Using a Fitbit Device in Ischemic Stroke Patients: Prospective Cohort Feasibility Study. JMIR mHealth uHealth.

[42] Zhang Z. (2016). Case-crossover design and its implementation in R. Ann. Transl. Med..

[43] Wang X., Wang S., Kindzierski W. (2019). Time-stratified case-crossover design applied with conditional logistic regression is not free from overlap bias. Stat. Methods Med. Res..

[44] Bonnesen M.P., Frodi D.M., Haugan K.J., Kronborg C., Graff C., Højberg S., Køber L., Krieger D., Brandes A., Svendsen J.H. (2021). Day-to-day measurement of physical activity and risk of atrial fibrillation. Eur. Heart J..

[45] Blond K., Brinkløv C.F., Ried-Larsen M., Crippa A., Grøntved A. (2019). Association of high amounts of physical activity with mortality risk: A systematic review and meta-analysis. Br. J. Sports Med..

[46] Rosero S.Z., Younis A., Jones P., McNitt S., Goldenberg I., Zareba W., Stein K., Kutyifa V. (2021). Utility of cardiovascular implantable electronic device–derived patient activity to predict clinical outcomes. Heart Rhythm.

[47] Jędrzejczyk-Patej E., Kowalski O., Sredniawa B., Pruszkowska P., Sokal A., Szulik M., Mazurek M., Kowalczyk J., Kalarus Z., Lenarczyk R. (2014). Trying to predict the unpredictable: Variations in device-based daily monitored diagnostic parameters can predict malignant arrhythmic events in patients undergoing cardiac resynchronization therapy. Cardiol. J..

[48] Ramakrishnan R., Doherty A., Smith-Byrne K., Rahimi K., Bennett D., Woodward M., Walmsley R., Dwyer T. (2021). Accelerometer measured physical activity and the incidence of cardiovascular disease: Evidence from the UK Biobank cohort study. PLoS Med. Public Libr. Sci..

[49] Werhahn S.M., Dathe H., Rottmann T., Franke T., Vahdat D., Hasenfuß G., Seidler T. (2019). Designing meaningful outcome parameters using mobile technology: A new mobile application for telemonitoring of patients with heart failure. ESC Heart Fail..

[50] Curtis A.F., Roth A.J., Sears S.F., Conti J.B., Berry R.B., Dzierzewski J.M., McCrae C.S. (2020). Associations between pain, objective sleep efficiency and cognition in patients with implantable cardioverter defibrillators. Sleep Med..

[51] Rosenberger M.E., Fulton J.E., Buman M., Troiano R.P., Grandner M.A., Buchner D.M., Haskell W.L. (2019). The 24-Hour Activity Cycle: A New Paradigm for Physical Activity. Med. Sci. Sports Exerc..

[52] Klompstra L., Kyriakou M., Lambrinou E., Piepoli M.F., Coats A.J.S., Cohen-Solal A., Cornelis J., Gellen B., Marques-Sule E., Niederseer D. (2021). Measuring physical activity with activity monitors in patients with heart failure: From literature to practice. A position paper from the Committee on Exercise Physiology and Training of the Heart Failure Association of the European Society of Car-diology. Eur. J. Heart Fail..

[53] Henriksen A., Mikalsen M.H., Woldaregay A.Z., Muzny M., Hartvigsen G., Hopstock L.A., Grimsgaard S. (2018). Using Fitness Trackers and Smartwatches to Measure Physical Activity in Research: Analysis of Consumer Wrist-Worn Wearables. J. Med. Internet Res..

[54] De Bleser L., De Geest S., Vandenbroeck S., Vanhaecke J., Dobbels F. (2010). How accurate are electronic monitoring devices? A la-boratory study testing two devices to measure medication adherence. Sensors.

[55] Mayo N.E., Figueiredo S., Ahmed S., Bartlett S.J. (2017). Montreal Accord on Patient-Reported Outcomes (PROs) use series—Paper 2: Terminology proposed to measure what matters in health. J. Clin. Epidemiol..

