# Supplementary material for: Using Consumer-Wearable Activity Trackers for Risk Prediction of Life-Threatening Heart Arrhythmia in Patients with an Implantable Cardioverter-Defibrillator: An Exploratory Observational Study
Source: J Pers Med. 2022 Jun 8;12(6):942. doi: 10.3390/jpm12060942 (PMC9225164; doi:10.3390/jpm12060942)
Supplement: Supplementary file 1 [file jpm-12-00942-s001.zip › Listing S1_ Data Format Example.pdf]

Listing S1: Data Format Example

**Article**	Using Consumer-Wearable Activity Trackers for Risk Prediction of Life-Threatening Heart Arrhythmia in Patients with an Implantable Cardioverter-Defibrillator: An Exploratory Observational Study
**Authors**	Diana My Frodi*, Vlad Manea*, Søren Zöga Diederichsen, Jesper Hastrup Svendsen, Katarzyna Wac, Tariq Osman Andersen<br>* equal contribution
**Special Issue**	Cardiovascular Diseases—From Risk Factors to Diagnosis and Personalized Management
**Journal**	MDPI Journal of Personalized Medicine

Table of Contents

Table of Contents **1**

Cardiac Device Data **1**

all_episodes.csv 1

Fitbit Data **2**

activity_level_observations.csv 2

activity_levels.csv 2

heart_rate.csv 2

sleep_observations.csv 2

sleep_stages.csv 2

steps.csv 3

Cardiac Device Data

all_episodes.csv

```
,PseudoId,TransmissionId,TransmissionTime,StartTime,DeviceModel,DeviceType,TransmissionCode,EpisodeType,Duration,AverageVentricularRate,MaximumVentricularRate,ATPSequencesDelivered,Shock,ActivityAtOnset
0,1,46,2020-01-01 00:00:00.0000000 +00:00,2020-01-01 00:00:00.0000000 +00:00,Evera S VR,ICD,PATIENT_INITIATED,PATIENT_ACTIVATED,0.0,,,0,0,Unknown
```

Fitbit Data

activity_level_observations.csv

```
,PseudoId,ActivityTimestamp,ActivityLevel_Id
0,1,2020-01-01 00:00:00.0000000 +00:00,1
```

activity_levels.csv

```
,ActivityLevel
0,0
1,1
2,3
3,2
```

heart_rate.csv

```
,PseudoId,HRTimestamp,HR
0,1,2020-01-01 00:00:00.0000000 +00:00,60
```

sleep_observations.csv

```
,PseudoId,StepTimestamp,Duration,SleepStage_Id
0,1,2020-01-01 00:00:00.0000000 +00:00,4000,1
```

sleep_stages.csv

```
,SleepStage
0,asleep
1,awake
2,restless
3,wake
4,light
5,deep
6,rem
```

```
7,unknown
```

steps.csv

```
,PseudoId,StepTimestamp,Steps
0,1,2020-01-01 00:00:00.0000000 +00:00,10
```
