# Supplementary material for: Using Consumer-Wearable Activity Trackers for Risk Prediction of Life-Threatening Heart Arrhythmia in Patients with an Implantable Cardioverter-Defibrillator: An Exploratory Observational Study
Source: J Pers Med. 2022 Jun 8;12(6):942. doi: 10.3390/jpm12060942 (PMC9225164; doi:10.3390/jpm12060942)
Supplement: Supplementary file 1 [file jpm-12-00942-s001.zip › Listing S2_ Data Analysis Code.pdf]

Listing S2: Data Analysis Code

Article Using Consumer-Wearable Activity Trackers for Risk Prediction of Life-Threatening Heart Arrhythmia in Patients with an Implantable Cardioverter-Defibrillator: An Exploratory Observational Study

Authors Diana My Frodi*, Vlad Manea*, Søren Zöga Diederichsen, Jesper Hastrup Svendsen, Katarzyna Wac, Tariq Osman Andersen
* equal contribution

Special Issue Cardiovascular Diseases—From Risk Factors to Diagnosis and Personalized Management

Journal MDPI Journal of Personalized Medicine

Table of Contents

Table of Contents **1**
Versions **2**
Python 2
R 2
Code **2**
Python 2
constraints.py 2
data_preprocessor.py 3
days_converter.py 7
duration_processor.py 8
duration_validator.py 11
heart_rate_parser.py 22
main.ipynb 24
sleep_parser.py 45
variable_definitions.py 46
variable_derivator.py 59
variable_properties.py 61

R 64
main.r 64

Versions

Python

Interpreter	Environment	Libraries
• Python 3.9.8 64-bit	• Anaconda 2.1.1	• numpy 1.20.3<br>• pandas 1.3.4

R

Interpreter	Environment	Libraries
• R 4.1.2	• RStudio 2021.09.1 Build 372	• survival 3.3-1

Code

Python

constraints.py

```
class constraints:
   eps = 0.001

   # First grid search:
   # minimum_available_data_ratios_daily_active = [23.0 / 24.0, 21.0 / 24.0, 18.0 / 24.0]

   # Second grid search:
   # minimum_available_data_ratios_daily_active = [15.0 / 1440.0, 30.0 / 1440.0, 60.0 / 1440.0]

   # Third grid search:
   minimum_available_data_ratios_daily_active = [1.0 / 12.0, 2.0 / 12.0, 4.0 / 12.0, 8.0 / 12.0]
```

```
minimum_available_data_ratios_weekly = [0.51]

minimum_available_data_ratios_period = [0.51]

allows_zeros = [False]

allows_no_event_participants = [True]

event_types = ['VT', 'VF_VT', 'VT1', 'VT2']

maximum_weeks_before_after = 8

maximum_regression_allowed_significance = 0.05

regression_eps = 0.000001
```

data_preprocessor.py

```
from heart_rate_parser import heart_rate_parser
from sleep_parser import sleep_parser
from duration_processor import duration_processor
from days_converter import days_converter
from variable_derivator import variable_derivator
from variable_properties import variable_properties
from os import path

import numpy as np
import pandas as pd
import statistics

class data_preprocessor:
   def __init__(self, id_column, group_duration_column, date_numeric_column):
       self.id_column = id_column
       self.group_duration_column = group_duration_column
       self.date_numeric_column = date_numeric_column
```

```
    minutes_in_hour = 60
    hours_in_day = 24
    days_in_week = 7

    def preprocess_heart_rate(self, heart_rate_folder):
        heart_rate_file_source = heart_rate_folder + '/heart_rate.csv'
        heart_rate_file_destination = heart_rate_folder + '/heart_rate_aggregate.csv'

        if not path.exists(heart_rate_file_destination):
            print('Computing heart rates aggregate by minute.')

            aggregation_functions = {'HeartRateMin': min, 'HeartRateMax': max, 'HeartRateMean': statistics.mean, 'HeartRateMedian': statistics.median,
'HeartRateStdev': statistics.stdev}
            aggregation_minimum_sizes = {'HeartRateMin': 1, 'HeartRateMax': 1, 'HeartRateMean': 1, 'HeartRateMedian': 1, 'HeartRateStdev': 2}
            aggregation_defaults = {'HeartRateMin': np.nan, 'HeartRateMax': np.nan, 'HeartRateMean': np.nan, 'HeartRateMedian': np.nan, 'HeartRateStdev':
np.nan}
            quantiles = []#0.0, 0.01, 0.025, 0.05, 0.1, 0.25, 0.33, 0.5, 0.67, 0.75, 0.9, 0.95, 0.975, 1.0]
            aggregate_interval = variable_properties.data_frequency_minutes

            for quantile in quantiles:
                key = 'HeartRateQuantile' + str(quantile)
                aggregation_functions[key] = data_preprocessor.__get_quantile_function(quantile)
                aggregation_minimum_sizes[key] = 1
                aggregation_defaults[key] = np.nan

            heart_rate_par = heart_rate_parser(aggregation_functions, aggregation_minimum_sizes, aggregation_defaults, reporting_progress_count = 100000)
            df_heart_rate_aggregate = heart_rate_par.parse(heart_rate_file_source, self.id_column, self.group_duration_column, aggregate_interval)
            df_heart_rate_aggregate.to_csv(heart_rate_file_destination)

    def preprocess_user(self, user_id, minutely_expanded_selected_file, df_steps_observations_all, df_activity_observations_all,
df_sleep_observations_all, df_heart_rate_aggregate_all):
        variable_der = variable_derivator(self.date_numeric_column)
        print('- Preprocessing for user: {}'.format(user_id))

        if not path.exists(minutely_expanded_selected_file):
```

```
        days_conv = days_converter(self.group_duration_column)

        scale_from_days = data_preprocessor.minutes_in_hour * data_preprocessor.hours_in_day
        df_sleep_observations_user = df_sleep_observations_all[df_sleep_observations_all[self.id_column] == user_id]
        df_steps_observations_user = df_steps_observations_all[df_steps_observations_all[self.id_column] == user_id]
        df_activity_observations_user = df_activity_observations_all[df_activity_observations_all[self.id_column] == user_id]
        df_heart_rate_aggregate_user = df_heart_rate_aggregate_all[df_heart_rate_aggregate_all[self.id_column] == user_id]

        # Sleep.
        print('-> Processing sleep')
        df_sleep_observations = days_conv.populate_with_date(df_sleep_observations_user, 'StepTimestamp')
        df_sleep_observations = sleep_parser.parse(df_sleep_observations, self.id_column, self.group_duration_column, 'SleepStage_Id', 'Duration')

        # Steps.
        print('-> Processing steps')
        df_steps_observations = days_conv.populate_with_date(df_steps_observations_user, 'StepTimestamp')
        df_steps_count = duration_processor.compute_duration_metric_count(df_steps_observations, self.id_column, 'StepsCount', 'Steps',
self.group_duration_column)

        # Activity.
        print('-> Processing activity')
        df_activity_observations = days_conv.populate_with_date(df_activity_observations_user, 'ActivityTimestamp')
        df_activity_level_one_hot = pd.get_dummies(df_activity_observations['ActivityLevel_Id'], prefix = 'ActivityLevel_Id_')
        df_activity_observations = df_activity_observations.drop('ActivityLevel_Id', axis = 1)
        df_activity_observations = df_activity_observations.join(df_activity_level_one_hot)

        # Heart rate.
        print('-> Processing heart rate')
        # Do nothing here, not needed.
        df_heart_rate_aggregate = df_heart_rate_aggregate_user

        print('-> Processing minutely')
        df_minutely, columns_minutely = duration_processor.compute_duration_dataframe([df_steps_observations, df_heart_rate_aggregate,
df_activity_observations, df_sleep_observations], self.id_column, self.group_duration_column)
        df_minutely = pd.merge(df_minutely, df_steps_count, on = columns_minutely, how = 'outer')
        df_minutely = pd.merge(df_minutely, df_heart_rate_aggregate, on = columns_minutely, how = 'outer')
```

```
            df_minutely = pd.merge(df_minutely, df_activity_observations, on = columns_minutely, how = 'outer')
            df_minutely = pd.merge(df_minutely, df_sleep_observations, on = columns_minutely, how = 'outer')

            if self.group_duration_column in df_minutely.columns:
                func = lambda value: value // (data_preprocessor.hours_in_day * data_preprocessor.minutes_in_hour)
                df_minutely[self.date_numeric_column] = df_minutely[self.group_duration_column].map(func)

            df_minutely.sort_values(by = [self.id_column, self.group_duration_column], inplace = True)
            print('-> Minutely done')

            df_minutely_expanded = variable_der.derive(df_minutely)
            df_minutely_expanded.to_csv(minutely_expanded_selected_file)
            print('-> Minutely expanded done')

    def get_wearable_user_ids(self, df_steps_observations_all, df_activity_observations_all, df_sleep_observations_all, df_heart_rate_aggregate_all):
        wearable_user_ids = set()
        wearable_user_ids.update(df_steps_observations_all[self.id_column].unique())
        wearable_user_ids.update(df_activity_observations_all[self.id_column].unique())
        wearable_user_ids.update(df_sleep_observations_all[self.id_column].unique())
        wearable_user_ids.update(df_heart_rate_aggregate_all[self.id_column].unique())
        return wearable_user_ids

    def get_pacemaker_user_ids(self, df_cardiac):
        pacemaker_user_ids = set()
        pacemaker_user_ids.update(df_cardiac[self.id_column].unique())
        return pacemaker_user_ids

    def get_intersection_user_ids(self, df_cardiac, df_steps_observations_all, df_activity_observations_all, df_sleep_observations_all,
df_heart_rate_aggregate_all):
        wearable_user_ids = self.get_wearable_user_ids(df_steps_observations_all, df_activity_observations_all, df_sleep_observations_all,
df_heart_rate_aggregate_all)
        pacemaker_user_ids = self.get_pacemaker_user_ids(df_cardiac)
        return pacemaker_user_ids.intersection(wearable_user_ids)

    def get_union_user_ids(self, df_cardiac, df_steps_observations_all, df_activity_observations_all, df_sleep_observations_all,
df_heart_rate_aggregate_all):
```

```
        wearable_user_ids = self.get_wearable_user_ids(df_steps_observations_all, df_activity_observations_all, df_sleep_observations_all,
df_heart_rate_aggregate_all)
        pacemaker_user_ids = self.get_pacemaker_user_ids(df_cardiac)
        return pacemaker_user_ids.union(wearable_user_ids)

    @staticmethod
    def __get_quantile_function(quantile):
        def quant(values):
            return np.quantile(values, quantile)

        return quant
```

days_converter.py

```
import numpy as np
from datetime import datetime, timedelta

class days_converter:
    start_date = datetime(2016, 1, 1)

    def __init__(self, group_duration_column):
        self.group_duration_column = group_duration_column

    def populate_with_date(self, df, datetime_column_name):
        df['DateNumeric'] = df[datetime_column_name].map(days_converter.convert_to_days)
        df[self.group_duration_column] = df[datetime_column_name].map(days_converter.convert_to_minutes)
        df['WeekNumeric'] = df[datetime_column_name].map(lambda row: days_converter.convert_to_days(row) // 7)
        return df

    @staticmethod
    def convert_to_days(date_str):
        date = np.nan

        if '+00:00' not in date_str:
            raise('Does not have +00:00')
```

```python
        try:
            date = datetime.strptime(date_str[0:10], '%Y-%m-%d')
        except:
            return np.nan

        days = (date - days_converter.start_date).days
        return int(days)

    @staticmethod
    def convert_to_minutes(date_str):
        date = np.nan

        if '+00:00' not in date_str:
            raise('Does not have +00:00')

        try:
            date = datetime.strptime(date_str[0:16], '%Y-%m-%d %H:%M')
        except:
            return np.nan

        days = days_converter.convert_to_days(date_str)
        minutes = days * 1440 + date.hour * 60 + date.minute
        return minutes

    @staticmethod
    def get_date(date_numeric):
        return days_converter.start_date + timedelta(days = date_numeric)
```

duration_processor.py

```python
import pandas as pd
import numpy as np
from datetime import datetime

class duration_processor:
```

```python
    @staticmethod
    def compute_duration_dataframe(dfs, id_column, group_duration_column):
        mapping_users_durations = dict()

        for df in dfs:
            df_grouped_users = df.groupby(id_column)

            for user_id, df_group_user in df_grouped_users:
                mapping_users_durations[user_id] = mapping_users_durations.get(user_id, set())
                df_grouped_duration = df_group_user.groupby(group_duration_column)

                for duration_numeric, df_group_duration in df_grouped_duration:
                    mapping_users_durations[user_id].add(duration_numeric)

        rows_pd = []

        for user_id, durations_numeric in mapping_users_durations.items():
            for duration_numeric in durations_numeric:
                new_row_pd = [user_id, duration_numeric]
                rows_pd.append(new_row_pd)

        columns = [id_column, group_duration_column]
        df_duration = pd.DataFrame(rows_pd, columns = columns)

        #print(df_duration)
        return (df_duration, columns)

    @staticmethod
    def compute_duration_metric_count(df, id_column, new_name_column, value_column, group_duration_column, filter_column = None, filter_value = None):
        columns = [id_column, group_duration_column, new_name_column]
        df_grouped_users = df.groupby(id_column)
        results_pd = []

        for user_id, df_group_user in df_grouped_users:
            df_grouped_duration = df_group_user.groupby(group_duration_column)
            duration_pd = []
```

```
        for duration_numeric, df_group_duration in df_grouped_duration:
            total_value = 0

            for index, row in df_group_duration.iterrows():
                if (filter_column is None) or (row[filter_column] == filter_value):
                    total_value += row[value_column]

            if total_value > 0:
                new_row = [user_id, duration_numeric, total_value]
                duration_pd.append(new_row)

                #print('Raw:')
                #print(df_group_duration)
                #print('Processed:')
                #print(new_row)

        results_pd.extend(duration_pd)

    df_result = pd.DataFrame(results_pd, columns = columns)
    return df_result

@staticmethod
def __get_seconds(duration_str):
    if '+00:00' not in duration_str:
        raise('Does not have +00:00')

    try:
        duration_time = datetime.strptime(duration_str[0:19], '%Y-%m-%d %H:%M:%S')
        duration = datetime.strptime(duration_str[0:10], '%Y-%m-%d')
    except:
        return np.nan

    seconds = (duration_time - duration).seconds
    return int(seconds)
```

duration_validator.py

```
import numpy as np

from variable_definitions import variable_definitions
from variable_properties import variable_properties
from constraints import constraints

class duration_validator:
   minutes_in_hour = 60
   hours_in_day = 24
   days_in_week = 7

   def __init__(self, date_numeric_column, drop_column_name, minimum_available_data_ratio_daily_active, minimum_available_data_ratio_weekly,
minimum_available_data_ratio_period, allow_zeros):
       self.date_numeric_column = date_numeric_column
       self.drop_column_name = drop_column_name
       self.minimum_available_data_ratio_daily_active = minimum_available_data_ratio_daily_active
       self.minimum_available_data_ratio_weekly = minimum_available_data_ratio_weekly
       self.minimum_available_data_ratio_period = minimum_available_data_ratio_period
       self.allow_zeros = allow_zeros

   @staticmethod
   def get_first_day(cardiac_date, weeks_before, leeway):
       return cardiac_date - 7 * weeks_before * (1 + leeway)

   @staticmethod
   def get_last_day(cardiac_date, weeks_before, leeway):
       return duration_validator.get_first_day(cardiac_date, weeks_before, leeway) + 6

   @staticmethod
   def get_last_day_period(cardiac_date, weeks_before, leeway):
       return duration_validator.get_first_day(cardiac_date, weeks_before, leeway) + 7 * weeks_before - 1

   @staticmethod
   def get_hour(timestamp):
       try:
```

```
            return int(timestamp[11:13])
        except:
            return -1

    def __is_event_conflict(self, first_day, cardiac_date, df_cardiac_user):
        count_cardiac_events_any_day = df_cardiac_user[(first_day <= df_cardiac_user[self.date_numeric_column]) &
(df_cardiac_user[self.date_numeric_column] < cardiac_date)].shape[0]
        return (count_cardiac_events_any_day > 0)

    def __is_valid_day_by_count(self, df_minutely_date):

        # First and second grid search:
        # df_daily_time_total_dropna = df_minutely_date.dropna(subset = [self.drop_column_name])
        # df_daily_time_total_zeros = df_daily_time_total_dropna[(df_daily_time_total_dropna[self.drop_column_name] < -constraints.eps) |
(df_daily_time_total_dropna[self.drop_column_name] > constraints.eps)]

        df_daily_time_total_daylight = df_minutely_date[df_minutely_date.apply(lambda row: 8 <= duration_validator.get_hour(row['ActivityTimestamp']) and
duration_validator.get_hour(row['ActivityTimestamp']) <= 19, axis=1)]
        df_daily_time_total_dropna = df_daily_time_total_daylight.dropna(subset = [self.drop_column_name])
        df_daily_time_total_zeros = df_daily_time_total_dropna[(df_daily_time_total_dropna[self.drop_column_name] < -constraints.eps) |
(df_daily_time_total_dropna[self.drop_column_name] > constraints.eps)]

        bar_size = df_daily_time_total_zeros.shape[0] * 1.0

        # First and second grid search:
        # expected_bar_size = 1.0 * duration_validator.minutes_in_hour * duration_validator.hours_in_day * self.minimum_available_data_ratio_daily_active
/ variable_properties.data_frequency_minutes

        # Third grid search:
        expected_bar_size = 1.0 * duration_validator.minutes_in_hour * duration_validator.hours_in_day * self.minimum_available_data_ratio_daily_active /
variable_properties.data_frequency_minutes / 2.0

        is_valid = bar_size >= expected_bar_size

        return (is_valid, bar_size, expected_bar_size)
```

```python
def get_valid_days(self, df_minutely_expanded_selected, df_cardiac_user):
    valid_days = dict()

    min_valid_bar = float('inf')
    max_valid_bar = float('-inf')

    for date_numeric, df_minutely_date in df_minutely_expanded_selected.groupby(self.date_numeric_column):
        invalid_reason = None
        (is_valid, bar_size, expected_bar_size) = self.__is_valid_day_by_count(df_minutely_date)

        min_valid_bar = min(min_valid_bar, expected_bar_size)
        max_valid_bar = max(max_valid_bar, expected_bar_size)

        if is_valid:
            if self.__is_event_conflict(date_numeric, date_numeric + 1, df_cardiac_user):
                invalid_reason = 'conflict with event'
        else:
            invalid_reason = 'not enough entries'

        if is_valid and (invalid_reason is None):
            valid_days[date_numeric] = bar_size

    # print('Min valid bar: {}'.format(min_valid_bar))
    # print('Max valid bar: {}'.format(max_valid_bar))

    return valid_days

def get_valid_weeks(self, valid_days, df_cardiac_user):
    valid_weeks = dict()
    minimum_available_data_ratios_weekly = self.minimum_available_data_ratio_weekly

    valid_user_dates = set()

    for valid_user_date in valid_days.keys():
        valid_range = range(valid_user_date - 8, valid_user_date + 8)
        valid_user_dates.update(valid_range)
```

```python
        valid_user_dates = sorted(valid_user_dates)

        for cardiac_user_date in valid_user_dates:
            is_valid = False
            invalid_reason = None
            first_day = duration_validator.get_first_day(cardiac_user_date, 1, 0)
            last_day = duration_validator.get_last_day(cardiac_user_date, 1, 0)

            count_valid_days = 0

            for any_day in range(first_day, last_day + 1):
                if any_day in valid_days:
                    count_valid_days = 1 + count_valid_days

            if not self.__is_event_conflict(first_day, cardiac_user_date, df_cardiac_user):
                if count_valid_days >= 1.0 * minimum_available_data_ratios_weekly * duration_validator.days_in_week:
                    is_valid = True
                else:
                    invalid_reason = 'not enough entries'
            else:
                invalid_reason = 'conflict with event'

            if is_valid and (invalid_reason is None):
                valid_weeks[cardiac_user_date] = count_valid_days

        return valid_weeks

    def get_valid_periods(self, valid_weeks, df_cardiac_user, max_weeks_before_after):
        valid_periods = dict()
        minimum_available_data_ratio_period = self.minimum_available_data_ratio_period

        valid_user_dates = set()

        for valid_user_date in valid_weeks.keys():
            valid_range = range(valid_user_date - 8 * max_weeks_before_after, valid_user_date + 8 * max_weeks_before_after + 1)
```

```
            valid_user_dates.update(valid_range)

        valid_user_dates = sorted(valid_user_dates)

        for cardiac_user_date in valid_user_dates:
            valid_periods[cardiac_user_date] = valid_periods.get(cardiac_user_date, dict())

            for leeway in [0]:
                valid_periods[cardiac_user_date][leeway] = valid_periods[cardiac_user_date].get(leeway, dict())
                max_weeks_limit = max_weeks_before_after // (1 + leeway)

                for week_before_after in range(1, 1 + max_weeks_limit):
                    is_valid = False
                    invalid_reason = None
                    first_day = duration_validator.get_first_day(cardiac_user_date, week_before_after, leeway)

                    valid_weeks_inside = [valid_week for valid_week in range(1, 1 + week_before_after) if (cardiac_user_date - valid_week *
duration_validator.days_in_week + duration_validator.days_in_week) in valid_weeks]
                    count_valid_weeks = len(valid_weeks_inside)

                    if not self.__is_event_conflict(first_day, cardiac_user_date, df_cardiac_user):
                        if count_valid_weeks >= 1.0 * week_before_after * minimum_available_data_ratio_period:
                            is_valid = True
                        else:
                            invalid_reason = 'not enough entries'
                    else:
                        invalid_reason = 'conflict with event'

                    if is_valid and (invalid_reason is None):
                        valid_periods[cardiac_user_date][leeway][week_before_after] = count_valid_weeks

        return valid_periods

    def __compute_means(self, valid_days, df_duration):
        target_column_daily_aggregation_functions = variable_properties.target_column_daily_aggregation_functions
        values = dict()
```

```
        for valid_day, bar_size in valid_days.items():
            df_duration_day = df_duration[df_duration[self.date_numeric_column] == valid_day]

            for variable_name, aggregates in target_column_daily_aggregation_functions.items():
                df_duration_day_var_dropna = df_duration_day.dropna(subset = [variable_name])

                df_duration_day_var_zeros = df_duration_day_var_dropna

                if not self.allow_zeros:
                    df_duration_day_var_zeros = df_duration_day_var_dropna[(df_duration_day_var_dropna[variable_name] < -constraints.eps) |
(df_duration_day_var_dropna[variable_name] > constraints.eps)]

                if df_duration_day_var_zeros.shape[0] <= 0:
                    continue

                for aggregate_name, aggregate_func in aggregates.items():
                    aggregate_value = aggregate_func(df_duration_day_var_zeros[variable_name])

                    if not np.isnan(aggregate_value):
                        values[variable_name] = values.get(variable_name, dict())
                        values[variable_name][aggregate_name] = values[variable_name].get(aggregate_name, list())
                        values[variable_name][aggregate_name].append(aggregate_value)

        values_means = dict()
        values_stds = dict()

        for variable_name, aggregations in values.items():
            values_means[variable_name] = values_means.get(variable_name, dict())
            values_stds[variable_name] = values_stds.get(variable_name, dict())

            for aggregate_name, values in aggregations.items():
                values_means[variable_name][aggregate_name] = np.mean(values)
                values_stds[variable_name][aggregate_name] = np.std(values)

        return (values_means, values_stds)
```

```python
def get_valid_daily_aggregates(self, user_id, df_minutely, valid_days):
    day_rows = []
    target_column_daily_aggregation_functions = variable_properties.target_column_daily_aggregation_functions
    behaviour_column_names = variable_definitions.behaviour_target_variable_names

    # Non-compositional.
    for valid_day, valid_day_count in valid_days.items():
        df_day = df_minutely[df_minutely[self.date_numeric_column] == valid_day]

        for column in behaviour_column_names:
            if column not in df_day:
                continue

            df_column_dropna = df_day.dropna(subset = [column])

            df_column_zeros = df_column_dropna

            if not self.allow_zeros:
                df_column_zeros = df_column_dropna[(df_column_dropna[column] < -constraints.eps) | (df_column_dropna[column] > constraints.eps)]

            df_column_col = df_column_zeros[column]
            values_size = len(df_column_col)

            if column not in target_column_daily_aggregation_functions:
                continue

            aggregate_functions = target_column_daily_aggregation_functions[column]

            for aggregate_function_name, aggregate_function in aggregate_functions.items():
                value = aggregate_function(df_column_col)
                day_row = [user_id, valid_day, column, aggregate_function_name, values_size, value]
                day_rows.append(day_row)

    return day_rows
```

```python
def get_valid_weekly_aggregates(self, user_id, df_cardiac_user, df_daily_values_user, valid_weeks):
    event_column_names = variable_definitions.event_target_variable_names
    minimum_available_data_ratios_weekly = self.minimum_available_data_ratio_weekly

    week_rows = []

    # valid_weeks[cardiac_user_date][week_before_after] = count_valid_days

    for cardiac_user_date, valid_weeks_date in valid_weeks.items():
        df_cardiac_event = df_cardiac_user[df_cardiac_user[self.date_numeric_column] == cardiac_user_date]
        has_event = False

        # Get the event information.
        event_properties_lists = dict()
        for idx, row in df_cardiac_event.iterrows():
            has_event = True

            for event_column_name in event_column_names:
                event_properties_lists[event_column_name] = event_properties_lists.get(event_column_name, list())
                event_properties_lists[event_column_name].append(row[event_column_name])

        event_properties = dict()
        event_variable_aggregates = variable_properties.event_variable_aggregates
        for event_column_name, event_column_list in event_properties_lists.items():
            aggregate =  event_variable_aggregates[event_column_name]
            event_properties[event_column_name] = aggregate(event_properties_lists[event_column_name])

        for event_column_name in event_column_names:
            if event_column_name not in event_properties:
                event_properties[event_column_name] = None

        first_day = duration_validator.get_first_day(cardiac_user_date, 1, 0)
        last_day = duration_validator.get_last_day(cardiac_user_date, 1, 0)
        df_daily_values_days = df_daily_values_user[df_daily_values_user[self.date_numeric_column].isin(range(first_day, last_day + 1))]

        for column, df_daily_values_column in df_daily_values_days.groupby('variable_name'):
```

```
            if column not in variable_properties.target_column_weekly_aggregation_functions:
                continue

            for daily_aggregate_function_name, df_daily_values_daily_aggregate in df_daily_values_column.groupby('daily_aggregate'):
                daily_values_dropna = df_daily_values_daily_aggregate.dropna(subset = ['value'])

                daily_values_zeros = daily_values_dropna

                if not self.allow_zeros:
                    daily_values_zeros = daily_values_dropna[(daily_values_dropna['value'] < -constraints.eps) | (daily_values_dropna['value'] >
constraints.eps)]

                values = daily_values_zeros['value']
                values_size = len(values)
                sum_sizes = np.sum(daily_values_zeros['count_entries_day'])

                if values_size < minimum_available_data_ratios_weekly * duration_validator.days_in_week:
                    continue

                for weekly_aggregate_function_name, weekly_aggregate_function in
variable_properties.target_column_weekly_aggregation_functions[column].items():
                    value = weekly_aggregate_function(values)
                    week_row = [user_id, first_day, last_day, cardiac_user_date] + [event_properties[event_column_name] for event_column_name in
event_column_names] + [1, column, daily_aggregate_function_name, weekly_aggregate_function_name, values_size, sum_sizes, value, has_event]
                    week_rows.append(week_row)

        return week_rows

    def get_valid_period_aggregates(self, user_id, df_cardiac_user, df_weekly_values_user, valid_periods):
        event_column_names = variable_definitions.event_target_variable_names

        period_rows = []

        for cardiac_user_date, valid_leeways_date in valid_periods.items():
            df_cardiac_event = df_cardiac_user[df_cardiac_user[self.date_numeric_column] == cardiac_user_date]
            has_event = False
```

```python
        # Get the event information.
        event_properties_lists = dict()
        event_variable_aggregates = variable_properties.event_variable_aggregates
        for idx, row in df_cardiac_event.iterrows():
            has_event = True

            for event_column_name in event_column_names:
                event_properties_lists[event_column_name] = event_properties_lists.get(event_column_name, list())
                event_properties_lists[event_column_name].append(row[event_column_name])

        event_properties = dict()
        for event_column_name, event_column_list in event_properties_lists.items():
            aggregate =  event_variable_aggregates[event_column_name]
            event_properties[event_column_name] = aggregate(event_properties_lists[event_column_name])

        for event_column_name in event_column_names:
            if event_column_name not in event_properties:
                event_properties[event_column_name] = None

        for leeway, valid_weeks_leeway_date in valid_leeways_date.items():
            if leeway != 0:
                continue

            for week_before_after, count_valid_weeks in valid_weeks_leeway_date.items():
                first_day = duration_validator.get_first_day(cardiac_user_date, week_before_after, leeway)
                last_day = duration_validator.get_last_day_period(cardiac_user_date, week_before_after, leeway)

                all_user_dates = range(cardiac_user_date, cardiac_user_date - 7 * week_before_after, -7)
                df_weekly_values_week = df_weekly_values_user[df_weekly_values_user['cardiac_user_date'].isin(all_user_dates)]

                for column, df_weekly_values_column in df_weekly_values_week.groupby('variable_name'):
                    if column not in variable_properties.target_column_period_aggregation_functions:
                        continue

                    for daily_aggregate_function_name, df_weekly_values_daily_aggregate in df_weekly_values_column.groupby('daily_aggregate'):
```

```
                           for weekly_aggregate_function_name, df_weekly_values_weekly_aggregate in 
df_weekly_values_daily_aggregate.groupby('weekly_aggregate'):

                               # Compositional case.
                               if (weekly_aggregate_function_name == 'clr') and ('clr' not in column):
                                   continue

                               # Non-compositional case.
                               if (weekly_aggregate_function_name != 'clr') and ('clr' in column):
                                   continue

                               weekly_values_dropna = df_weekly_values_weekly_aggregate.dropna(subset = ['value'])
                               #weekly_values_zeros = weekly_values_dropna[(weekly_values_dropna['value'] < -constraints.eps) | 
(weekly_values_dropna['value'] > constraints.eps)]

                               # Check again that there are enough weeks with nonzero values in the period.
                               if weekly_values_dropna.shape[0] < 1.0 * self.minimum_available_data_ratio_period * week_before_after:
                                   continue

                               values = weekly_values_dropna['value']
                               values_size = len(values)
                               sum_sizes = np.sum(weekly_values_dropna['count_entries_days'])

                               for period_aggregate_function_name, period_aggregate_function in 
variable_properties.target_column_period_aggregation_functions[column].items():
                                   try:
                                       value = period_aggregate_function(values)

                                       if not np.isnan(value):
                                           period_row = [user_id, first_day, last_day, cardiac_user_date] + [event_properties[event_column_name] for 
event_column_name in event_column_names] + [leeway, week_before_after, column, daily_aggregate_function_name, weekly_aggregate_function_name, 
period_aggregate_function_name, values_size, sum_sizes, value, has_event]
                                           period_rows.append(period_row)
                                   except:
                                       pass
```

```
        return period_rows
```

heart_rate_parser.py

```
from days_converter import days_converter
import pandas as pd
import numpy as np

class heart_rate_parser:
    def __init__(self, aggregation_functions, aggregation_minimum_sizes, aggregation_defaults, reporting_progress_count = None):
        self.aggregation_functions = aggregation_functions
        self.aggregation_minimum_sizes = aggregation_minimum_sizes
        self.aggregation_defaults = aggregation_defaults
        self.reporting_progress_count = reporting_progress_count

    def __compute_aggregate_values(self, day_values):
        aggregate_values = []

        for aggregate_function_name, aggregate_function in self.aggregation_functions.items():
            size = len(day_values)
            aggregate_value = self.aggregation_defaults[aggregate_function_name]
            minimum_size = self.aggregation_minimum_sizes[aggregate_function_name]

            if size <= 0:
                aggregate_value = np.nan
            elif size >= minimum_size:
                aggregate_value = aggregate_function(day_values)

            aggregate_values.append(aggregate_value)

        return aggregate_values

    def parse(self, file_name, id_column, group_duration_column, aggregate_interval):
        entries = []

        with open(file_name) as infile:
```

```python
minute_values = []
is_first_line = True
minutes_conv = days_converter(group_duration_column)
last_user_id = None
last_interval_start = None
count = 0

for line in infile:
    if is_first_line:
        is_first_line = False
        continue

    if (self.reporting_progress_count is not None) and (count % self.reporting_progress_count == 0):
        print('Line: ' + str(count))

    split = line.split(',')
    user_id = split[1]

    timestamp = split[2]
    minute = minutes_conv.convert_to_minutes(timestamp)
    interval_start = minute // aggregate_interval

    if last_interval_start is not None and ((user_id != last_user_id) or (interval_start != last_interval_start)):
        computed_values = self.__compute_aggregate_values(minute_values)
        minute_values = []
        entries.append([last_user_id, last_interval_start * aggregate_interval] + computed_values)

    heart_rate = int(split[3])
    minute_values.append(heart_rate)
    last_user_id = user_id
    last_interval_start = interval_start

if len(minute_values) > 0:
    computed_values = self.__compute_aggregate_values(minute_values)
    entries.append([last_user_id, last_interval_start * aggregate_interval] + computed_values)
```

```
        columns = [id_column, group_duration_column] + list(self.aggregation_functions.keys())
        df = pd.DataFrame(entries, columns = columns)
        return df
```

main.ipynb

```
from os import path
from duration_validator import duration_validator
from data_preprocessor import data_preprocessor
from constraints import constraints
from days_converter import days_converter
from variable_definitions import variable_definitions
from variable_properties import variable_properties
from IPython.display import display

import pickle
import pandas as pd

pd.set_option('display.max_columns', None)

days_in_week = 7
hours_in_day = 24
minutes_in_hour = 60
id_column = 'PseudoId'
group_duration_column = 'MinutesNumeric'
date_numeric_column = 'DateNumeric'

# First grid search:
# drop_column_name = 'ActivityTimeTotal'

# Second and third grid search:
drop_column_name = 'ActivityTimeActive'

data_preproc = data_preprocessor(id_column, group_duration_column, date_numeric_column)
heart_rate_folder = 'data/original_data/fitbit_data'
data_preproc.preprocess_heart_rate(heart_rate_folder)
```

```
heart_rate_file_destination = heart_rate_folder + '/heart_rate_aggregate.csv'
df_heart_rate_aggregate_all = pd.read_csv(heart_rate_file_destination)
print('Heart rate users: {}'.format(df_heart_rate_aggregate_all[id_column].unique()))

sleep_file = 'data/original_data/fitbit_data/sleep_observations.csv'
df_sleep_observations_all = pd.read_csv(sleep_file)
print('Sleep users: {}'.format(df_sleep_observations_all[id_column].unique()))

steps_file = 'data/original_data/fitbit_data/steps.csv'
df_steps_observations_all = pd.read_csv(steps_file)
print('Steps users: {}'.format(df_steps_observations_all[id_column].unique()))

activity_file = 'data/original_data/fitbit_data/activity_level_observations.csv'
df_activity_observations_all = pd.read_csv(activity_file)
print('Activity users: {}'.format(df_activity_observations_all[id_column].unique()))

df_cardiac_file = 'data/original_data/cardiac_device_data/all_episodes.csv'
df_cardiac = pd.read_csv(df_cardiac_file)
days_conv = days_converter(group_duration_column)
df_cardiac = days_conv.populate_with_date(df_cardiac, 'StartTime')
event_types = constraints.event_types
df_cardiac = df_cardiac[df_cardiac['EpisodeType'].isin(event_types)]
print('Episode users: {}'.format(df_cardiac[id_column].unique()))

wearable_user_ids = data_preproc.get_wearable_user_ids(df_steps_observations_all, df_activity_observations_all, df_sleep_observations_all,
df_heart_rate_aggregate_all)
print('Wearable users: {}'.format(wearable_user_ids))

pacemaker_user_ids = data_preproc.get_pacemaker_user_ids(df_cardiac)
print('Pacemaker users: {}'.format(pacemaker_user_ids))

intersection_user_ids = data_preproc.get_intersection_user_ids(df_cardiac, df_steps_observations_all, df_activity_observations_all,
df_sleep_observations_all, df_heart_rate_aggregate_all)
print('Intersection users: {}'.format(intersection_user_ids))

union_user_ids = data_preproc.get_union_user_ids(df_cardiac, df_steps_observations_all, df_activity_observations_all, df_sleep_observations_all,
```

```python
df_heart_rate_aggregate_all)
print('Union users: {}'.format(union_user_ids))
```

```python
max_weeks_before_after = constraints.maximum_weeks_before_after
behaviours_aggregates = variable_properties.behaviours_aggregates
behaviour_column_names = variable_definitions.behaviour_target_variable_names

minutely_expanded_selected_file_format = 'data/derived_data/minutely_expanded_selected_user_{}_simplified.csv'

for user_id in wearable_user_ids:
   minutely_expanded_selected_file = minutely_expanded_selected_file_format.format(user_id)
   data_preproc.preprocess_user(user_id, minutely_expanded_selected_file, df_steps_observations_all, df_activity_observations_all,
df_sleep_observations_all, df_heart_rate_aggregate_all)

print('Wearable data preprocessed')
```

```python
df_cardiac_table = pd.read_csv(df_cardiac_file)
df_cardiac_table.drop_duplicates([id_column, 'DeviceModel', 'DeviceType'], inplace = True)
df_cardiac_table_users = df_cardiac_table[df_cardiac_table[id_column].isin(pacemaker_user_ids)]
df_cardiac_table_selected = df_cardiac_table_users[[id_column, 'DeviceModel', 'DeviceType']]
df_cardiac_table_selected.sort_values(by = [id_column], inplace = True)
display(df_cardiac_table_selected)
```

```python
rows_monitoring_period = []

interest_user_ids = [] # A list of Int, removed to protect the users
date_carry = 2616

for user_id in interest_user_ids:
   minutely_expanded_selected_file = minutely_expanded_selected_file_format.format(user_id)
   df_cardiac_user = df_cardiac[df_cardiac[id_column] == user_id]

   min_date_fitbit = None
   max_date_fitbit = None
   diff_fitbit = 0

   if path.exists(minutely_expanded_selected_file):
```

```
        df_minutely_expanded_selected = pd.read_csv(minutely_expanded_selected_file)
        min_date_fitbit = min(df_minutely_expanded_selected[date_numeric_column]) + date_carry
        max_date_fitbit = max(df_minutely_expanded_selected[date_numeric_column]) + date_carry
        diff_fitbit = max_date_fitbit - min_date_fitbit + 1

    min_date_event = None
    max_date_event = None
    diff_event = 0

    if df_cardiac_user.shape[0] > 0:
        min_date_event = min(df_cardiac_user[date_numeric_column]) + date_carry
        max_date_event = max(df_cardiac_user[date_numeric_column]) + date_carry
        diff_event = max_date_event - min_date_event + 1

    if min_date_fitbit is not None:
        if min_date_event is not None:
            monitoring_period = max(max_date_fitbit, max_date_event) - min(min_date_fitbit, min_date_event) + 1
        else:
            monitoring_period = diff_fitbit
    else:
        monitoring_period = diff_event

    row_monitoring_period = [user_id, min_date_fitbit, max_date_fitbit, diff_fitbit, min_date_event, max_date_event, diff_event, monitoring_period]
    rows_monitoring_period.append(row_monitoring_period)

df_monitoring_period = pd.DataFrame(rows_monitoring_period, columns = ['Patient ID', 'Min. date Fitbit', 'Max. date Fitbit', 'Diff. Fitbit', 'Min. date ICD', 'Max. date ICD', 'Diff. ICD', 'Monitoring period'])
display(df_monitoring_period)
```

```
compute_valid = True

df_valid_days_file_format = 'data/validation_data/valid_days_user_{}_mindaily_{}_minweekly_{}_minperiod_{}_zeros_{}_paired_{}.csv'
df_valid_weeks_file_format = 'data/validation_data/valid_weeks_user_{}_mindaily_{}_minweekly_{}_minperiod_{}_zeros_{}_paired_{}.csv'
df_valid_periods_file_format = 'data/validation_data/valid_periods_user_{}_mindaily_{}_minweekly_{}_minperiod_{}_zeros_{}_paired_{}.csv'

if compute_valid:
    for minimum_available_data_ratio_daily_active in constraints.minimum_available_data_ratios_daily_active:
```

```
        for minimum_available_data_ratio_weekly in constraints.minimum_available_data_ratios_weekly:
            for minimum_available_data_ratio_period in constraints.minimum_available_data_ratios_period:
                if minimum_available_data_ratio_weekly != minimum_available_data_ratio_period:
                    continue

                for allow_zeros in constraints.allows_zeros:
                    duration_val = duration_validator(date_numeric_column, drop_column_name, minimum_available_data_ratio_daily_active,
minimum_available_data_ratio_weekly, minimum_available_data_ratio_period, allow_zeros)

                    for allow_no_event_participants in constraints.allows_no_event_participants:
                        behaviours_aggregates = variable_properties.behaviours_aggregates

                        for user_id in wearable_user_ids:
                            if user_id not in intersection_user_ids:
                                continue

                            print(f"User: {user_id}")

                            # Get the valid days.
                            df_valid_days_file = df_valid_days_file_format.format(user_id, minimum_available_data_ratio_daily_active,
minimum_available_data_ratio_weekly, minimum_available_data_ratio_period, allow_zeros, allow_no_event_participants)

                            if (not path.exists(df_valid_days_file)):
                                minutely_expanded_selected_file = minutely_expanded_selected_file_format.format(user_id)
                                df_cardiac_user = df_cardiac[df_cardiac[id_column] == user_id]
                                df_minutely_expanded_selected = pd.read_csv(minutely_expanded_selected_file)

                                df_valid_days_file = df_valid_days_file_format.format(user_id, minimum_available_data_ratio_daily_active,
minimum_available_data_ratio_weekly, minimum_available_data_ratio_period, allow_zeros, allow_no_event_participants)
                                valid_days = None
                                invalid_days = None

                                valid_days = duration_val.get_valid_days(df_minutely_expanded_selected, df_cardiac_user)

                                if not path.exists(df_valid_days_file):
                                    with open(df_valid_days_file, 'wb') as f:
```

```
                                        pickle.dump(valid_days, f)

                            # Get the valid weeks.
                            df_valid_weeks_file = df_valid_weeks_file_format.format(user_id, minimum_available_data_ratio_daily_active,
minimum_available_data_ratio_weekly, minimum_available_data_ratio_period, allow_zeros, allow_no_event_participants)

                            if (not path.exists(df_valid_weeks_file)):
                                with open(df_valid_days_file, 'rb') as f:
                                    valid_days = pickle.load(f)

                                valid_weeks = None
                                invalid_weeks = None

                                valid_weeks = duration_val.get_valid_weeks(valid_days, df_cardiac_user)

                                if not path.exists(df_valid_weeks_file):
                                    with open(df_valid_weeks_file, 'wb') as f:
                                        pickle.dump(valid_weeks, f)

                            # Get the valid periods.
                            df_valid_periods_file = df_valid_periods_file_format.format(user_id, minimum_available_data_ratio_daily_active,
minimum_available_data_ratio_weekly, minimum_available_data_ratio_period, allow_zeros, allow_no_event_participants)

                            if (not path.exists(df_valid_periods_file)):
                                with open(df_valid_weeks_file, 'rb') as f:
                                    valid_weeks = pickle.load(f)

                                valid_periods = None
                                invalid_periods = None

                                valid_periods = duration_val.get_valid_periods(valid_weeks, df_cardiac_user, max_weeks_before_after)

                                if not path.exists(df_valid_periods_file):
                                    with open(df_valid_periods_file, 'wb') as f:
                                        pickle.dump(valid_periods, f)
```

```
df_valid_days_file_format = 'data/validation_data/valid_days_user_{}_mindaily_{}_minweekly_{}_minperiod_{}_zeros_{}_paired_{}.csv'
```

```python
for minimum_available_data_ratio_daily in constraints.minimum_available_data_ratios_daily_active:
   print(f"Minimum available data ratio daily: {minimum_available_data_ratio_daily}")

   for minimum_available_data_ratio_weekly in [0.51]:
       for minimum_available_data_ratio_period in [0.51]:
           if minimum_available_data_ratio_weekly != minimum_available_data_ratio_period:
               continue

           for allow_zeros in [False]:
               duration_val = duration_validator(date_numeric_column, drop_column_name, minimum_available_data_ratio_daily,
minimum_available_data_ratio_weekly, minimum_available_data_ratio_period, allow_zeros)

               for allow_no_event_participants in [True]:
                   behaviours_aggregates = variable_properties.behaviours_aggregates

                   for user_id in sorted(wearable_user_ids):
                       valid_days = 0
                       invalid_days = 0

                       # Get the valid days.
                       df_valid_days_file = df_valid_days_file_format.format(user_id, minimum_available_data_ratio_daily,
minimum_available_data_ratio_weekly, minimum_available_data_ratio_period, allow_zeros, allow_no_event_participants)

                       with open(df_valid_days_file, 'rb') as f:
                           valid_days_map = pickle.load(f)
                           valid_days = len(valid_days_map.keys())

                       print(f'{valid_days}')
```

```python
for user_id in intersection_user_ids:
   max_valid_days = float('-inf')
   min_valid_days = float('inf')

   max_valid_weeks = float('-inf')
   min_valid_weeks = float('inf')
```

```
    max_valid_periods = float('-inf')
    min_valid_periods = float('inf')

    max_valid_days_meta = None
    min_valid_days_meta = None

    max_valid_weeks_meta = None
    min_valid_weeks_meta = None

    max_valid_periods_meta = None
    min_valid_periods_meta = None

    for allow_zeros in constraints.allows_zeros:
        # print('- Allow zeros: {}'.format(allow_zeros))

        for allow_no_event_participants in constraints.allows_no_event_participants:
            # print('-- Allow no event participants: {}'.format(allow_no_event_participants))

            for minimum_available_data_ratio_daily_active in constraints.minimum_available_data_ratios_daily_active:
                # print('--- Daily limit: {}'.format(minimum_available_data_ratio_daily))

                for minimum_available_data_ratio_weekly in constraints.minimum_available_data_ratios_weekly:
                    # print('---- Weekly limit: {}'.format(minimum_available_data_ratio_weekly))

                    for minimum_available_data_ratio_period in constraints.minimum_available_data_ratios_period:
                        if minimum_available_data_ratio_weekly != minimum_available_data_ratio_period:
                            continue

                        # print('----- Period limit: {}'.format(minimum_available_data_ratio_period))

                        valid_days_file = "data/validation_data/valid_days_user_{}_mindaily_{}_minweekly_{}_minperiod_{}_zeros_{}_paired_{}.csv"\
                            .format(user_id, minimum_available_data_ratio_daily_active, minimum_available_data_ratio_weekly,
minimum_available_data_ratio_period, allow_zeros, allow_no_event_participants)

                        with open(valid_days_file, 'rb') as f:
                            valid_days = pickle.load(f)
```

```
                            len_valid_days = len(valid_days.keys())

                            if len_valid_days > max_valid_days:
                                max_valid_days = len_valid_days
                                max_valid_days_meta = [allow_zeros, allow_no_event_participants, minimum_available_data_ratio_daily_active,
minimum_available_data_ratio_weekly, minimum_available_data_ratio_period]

                            if len_valid_days < min_valid_days:
                                min_valid_days = len_valid_days
                                min_valid_days_meta = [allow_zeros, allow_no_event_participants, minimum_available_data_ratio_daily_active,
minimum_available_data_ratio_weekly, minimum_available_data_ratio_period]

                        valid_weeks_file = "data/validation_data/valid_weeks_user_{}_mindaily_{}_minweekly_{}_minperiod_{}_zeros_{}_paired_{}.csv"\
                            .format(user_id, minimum_available_data_ratio_daily_active, minimum_available_data_ratio_weekly,
minimum_available_data_ratio_period, allow_zeros, allow_no_event_participants)

                        with open(valid_weeks_file, 'rb') as f:
                            valid_weeks = pickle.load(f)
                            len_valid_weeks = len(valid_weeks.keys())

                            if len_valid_weeks > max_valid_weeks:
                                max_valid_weeks = len_valid_weeks
                                max_valid_weeks_meta = [allow_zeros, allow_no_event_participants, minimum_available_data_ratio_daily_active,
minimum_available_data_ratio_weekly, minimum_available_data_ratio_period]

                            if len_valid_weeks < min_valid_weeks:
                                min_valid_weeks = len_valid_weeks
                                min_valid_weeks_meta = [allow_zeros, allow_no_event_participants, minimum_available_data_ratio_daily_active,
minimum_available_data_ratio_weekly, minimum_available_data_ratio_period]

                        valid_periods_file = "data/validation_data/valid_periods_user_{}_mindaily_{}_minweekly_{}_minperiod_{}_zeros_{}_paired_{}.csv"\
                            .format(user_id, minimum_available_data_ratio_daily_active, minimum_available_data_ratio_weekly,
minimum_available_data_ratio_period, allow_zeros, allow_no_event_participants)

                        with open(valid_periods_file, 'rb') as f:
                            valid_periods = pickle.load(f)
```

```
                            len_valid_periods = len(valid_periods.keys())

                            if len_valid_periods > max_valid_periods:
                                max_valid_periods = len_valid_periods
                                max_valid_periods_meta = [allow_zeros, allow_no_event_participants, minimum_available_data_ratio_daily_active, 
minimum_available_data_ratio_weekly, minimum_available_data_ratio_period]

                            if len_valid_periods < min_valid_periods:
                                min_valid_periods = len_valid_periods
                                min_valid_periods_meta = [allow_zeros, allow_no_event_participants, minimum_available_data_ratio_daily_active, 
minimum_available_data_ratio_weekly, minimum_available_data_ratio_period]


   print(f'User: {user_id}')

   print(f'Diff valid days: {max_valid_days - min_valid_days} with min {min_valid_days} and max {max_valid_days}')
   print(f'Days min meta: {min_valid_days_meta}')
   print(f'Days max meta: {max_valid_days_meta}')

   print(f'Diff valid weeks: {max_valid_weeks - min_valid_weeks} with min {min_valid_weeks} and max {max_valid_weeks}')
   print(f'Weeks min meta: {min_valid_weeks_meta}')
   print(f'Weeks max meta: {max_valid_weeks_meta}')

   print(f'Diff valid periods: {max_valid_periods - min_valid_periods} with min {min_valid_periods} and max {max_valid_periods}')
   print(f'Periods min meta: {min_valid_periods_meta}')
   print(f'Periods max meta: {max_valid_periods_meta}')
```

```
daily_values_file_name_format = 'data/validation_data/valid_daily_aggregates_user_{}_mindaily_{}_minweekly_{}_minperiod_{}_zeros_{}_paired_{}.csv'
compute_daily_values = True

if compute_daily_values:
   for minimum_available_data_ratio_daily_active in constraints.minimum_available_data_ratios_daily_active:
       for minimum_available_data_ratio_weekly in constraints.minimum_available_data_ratios_weekly:
           for minimum_available_data_ratio_period in constraints.minimum_available_data_ratios_period:
               if minimum_available_data_ratio_weekly != minimum_available_data_ratio_period:
                   continue
```

```
            for allow_zeros in constraints.allows_zeros:
                for allow_no_event_participants in constraints.allows_no_event_participants:
                    for user_id in intersection_user_ids:
                        daily_values_file_name = daily_values_file_name_format.format(user_id, minimum_available_data_ratio_daily_active,
minimum_available_data_ratio_weekly, minimum_available_data_ratio_period, allow_zeros, allow_no_event_participants)

                        if path.exists(daily_values_file_name):
                            continue

                        print('Deriving the valid daily aggregates (wearable) for user: {}'.format(user_id))

                        minutely_file = minutely_expanded_selected_file_format.format(user_id)
                        df_minutely = pd.read_csv(minutely_file)

                        # Compute daily values.
                        df_valid_days_file = df_valid_days_file_format.format(user_id, minimum_available_data_ratio_daily_active,
minimum_available_data_ratio_weekly, minimum_available_data_ratio_period, allow_zeros, allow_no_event_participants)
                        valid_days = None

                        with open(df_valid_days_file, 'rb') as f:
                            valid_days = pickle.load(f)

                        day_rows_user = duration_val.get_valid_daily_aggregates(user_id, df_minutely, valid_days)
                        df_daily_values = pd.DataFrame(day_rows_user, columns = [id_column, date_numeric_column, 'variable_name', 'daily_aggregate',
'count_entries_day', 'value'])
                        df_daily_values.to_csv(daily_values_file_name)

   print('Done aggregates for valid days')
```

```
weekly_values_file_name_format = 'data/validation_data/valid_weekly_aggregates_user_{}_mindaily_{}_minweekly_{}_minperiod_{}_zeros_{}_paired_{}.csv'
compute_weekly_aggregates = True

if compute_weekly_aggregates:
   for user_id in intersection_user_ids:
       for minimum_available_data_ratio_daily_active in constraints.minimum_available_data_ratios_daily_active:
           for minimum_available_data_ratio_weekly in constraints.minimum_available_data_ratios_weekly:
               for minimum_available_data_ratio_period in constraints.minimum_available_data_ratios_period:
```

```
                    if minimum_available_data_ratio_weekly != minimum_available_data_ratio_period:
                        continue

                    for allow_zeros in constraints.allows_zeros:
                        for allow_no_event_participants in constraints.allows_no_event_participants:
                            weekly_values_file_name = weekly_values_file_name_format.format(user_id, minimum_available_data_ratio_daily_active,
minimum_available_data_ratio_weekly, minimum_available_data_ratio_period, allow_zeros, allow_no_event_participants)

                            if path.exists(weekly_values_file_name):
                                continue

                            print('Deriving the valid weekly aggregates (wearable) for user: {}'.format(user_id))

                            daily_values_file_name = daily_values_file_name_format.format(user_id, minimum_available_data_ratio_daily_active,
minimum_available_data_ratio_weekly, minimum_available_data_ratio_period, allow_zeros, allow_no_event_participants)
                            df_daily_values = pd.read_csv(daily_values_file_name)

                            df_cardiac_user = df_cardiac[df_cardiac[id_column] == user_id]
                            df_daily_values_user = df_daily_values[df_daily_values[id_column] == user_id]
                            minutely_file = minutely_expanded_selected_file_format.format(user_id)
                            df_minutely = pd.read_csv(minutely_file)

                            # Compute weekly values.
                            df_valid_weeks_file = df_valid_weeks_file_format.format(user_id, minimum_available_data_ratio_daily_active,
minimum_available_data_ratio_weekly, minimum_available_data_ratio_period, allow_zeros, allow_no_event_participants)
                            valid_weeks = None

                            with open(df_valid_weeks_file, 'rb') as f:
                                valid_weeks = pickle.load(f)

                            week_rows_user = duration_val.get_valid_weekly_aggregates(user_id, df_cardiac_user, df_daily_values_user, valid_weeks)

                            event_column_names = variable_definitions.event_target_variable_names
                            df_weekly_values = pd.DataFrame(week_rows_user, columns = [id_column, 'first_day', 'last_day', 'cardiac_user_date'] +
event_column_names + ['week_before_after', 'variable_name', 'daily_aggregate', 'weekly_aggregate', 'count_entries_week', 'count_entries_days', 'value',
'has_event'])
```

```
                           df_weekly_values.to_csv(weekly_values_file_name)

   print('Done aggregates for valid weeks')
```

```
period_values_file_name_format = 'data/validation_data/valid_period_aggregates_user_{}_mindaily_{}_minweekly_{}_minperiod_{}_zeros_{}_paired_{}.csv'
compute_period_aggregates = True

if compute_period_aggregates:
   for user_id in intersection_user_ids:
       for minimum_available_data_ratio_daily_active in constraints.minimum_available_data_ratios_daily_active:
           for minimum_available_data_ratio_weekly in constraints.minimum_available_data_ratios_weekly:
               for minimum_available_data_ratio_period in constraints.minimum_available_data_ratios_period:
                   if minimum_available_data_ratio_weekly != minimum_available_data_ratio_period:
                       continue

                   for allow_zeros in constraints.allows_zeros:
                       for allow_no_event_participants in constraints.allows_no_event_participants:
                           period_values_file_name = period_values_file_name_format.format(user_id, minimum_available_data_ratio_daily_active,
minimum_available_data_ratio_weekly, minimum_available_data_ratio_period, allow_zeros, allow_no_event_participants)

                           if path.exists(period_values_file_name):
                               continue

                           print('Deriving the valid period aggregates (wearable) for user: {}'.format(user_id))

                           weekly_values_file_name = weekly_values_file_name_format.format(user_id, minimum_available_data_ratio_daily_active,
minimum_available_data_ratio_weekly, minimum_available_data_ratio_period, allow_zeros, allow_no_event_participants)
                           df_weekly_values = pd.read_csv(weekly_values_file_name)

                           df_cardiac_user = df_cardiac[df_cardiac[id_column] == user_id]
                           df_weekly_values_user = df_weekly_values[df_weekly_values[id_column] == user_id]

                           # Compute weekly values.
                           df_valid_periods_file = df_valid_periods_file_format.format(user_id, minimum_available_data_ratio_daily_active,
minimum_available_data_ratio_weekly, minimum_available_data_ratio_period, allow_zeros, allow_no_event_participants)
                           valid_periods = None
```

```
                            with open(df_valid_periods_file, 'rb') as f:
                                valid_periods = pickle.load(f)

                            period_rows_user = duration_val.get_valid_period_aggregates(user_id, df_cardiac_user, df_weekly_values_user, valid_periods)
                            event_column_names = variable_definitions.event_target_variable_names
                            df_period_values = pd.DataFrame(period_rows_user, columns = [id_column, 'first_day', 'last_day', 'cardiac_user_date'] +
event_column_names + ['leeway', 'week_before_after', 'variable_name', 'daily_aggregate', 'weekly_aggregate', 'period_aggregate', 'count_entries_period',
'count_entries_days', 'value', 'has_event'])
                            df_period_values.sort_values(by = ['leeway', 'week_before_after', 'variable_name', 'daily_aggregate', 'weekly_aggregate',
'period_aggregate', id_column, 'cardiac_user_date'], ignore_index = True, inplace = True)
                            df_period_values.to_csv(period_values_file_name)

   print('Done aggregates for valid periods')
```

```
import json

compute_longitudinal_sizes = True
aggregates = ['mean', 'median', 'max', 'min', 'std', 'sum']

if compute_longitudinal_sizes:
   for minimum_available_data_ratio_daily in constraints.minimum_available_data_ratios_daily_active:
       for minimum_available_data_ratio_weekly in constraints.minimum_available_data_ratios_weekly:
           for minimum_available_data_ratio_period in constraints.minimum_available_data_ratios_period:
               if minimum_available_data_ratio_weekly != minimum_available_data_ratio_period:
                   continue

               for allow_zeros in constraints.allows_zeros:
                   for allow_no_event_participants in constraints.allows_no_event_participants:

                       # Mapping of: variable, period, daily, weekly, period, outcome, user:
                       mapping = dict()

                       for user_id in intersection_user_ids:
                           valid_aggregates_user_file =
'data/validation_data/valid_period_aggregates_user_{}_mindaily_{}_minweekly_{}_minperiod_{}_zeros_{}_paired_{}.csv'.format(user_id,
minimum_available_data_ratio_daily, minimum_available_data_ratio_weekly, minimum_available_data_ratio_period, allow_zeros, allow_no_event_participants)
```

```
                            if not path.exists(valid_aggregates_user_file):
                                continue

                            df_valid_periods_user = pd.read_csv(valid_aggregates_user_file)

                            for variable_name, df_variable in df_valid_periods_user.groupby('variable_name'):
                                for period, df_period in df_variable.groupby('week_before_after'):
                                    for daily_aggregate, df_daily_aggregate in df_period.groupby('daily_aggregate'):
                                        for weekly_aggregate, df_weekly_aggregate in df_daily_aggregate.groupby('weekly_aggregate'):
                                            for period_aggregate, df_period_aggregate in df_weekly_aggregate.groupby('period_aggregate'):
                                                for has_event, df_event in df_period_aggregate.groupby('has_event'):
                                                    if df_event.shape[0] <= 0:
                                                        continue

                                                    mapping[variable_name] = mapping.get(variable_name, dict())
                                                    mapping[variable_name][period] = mapping[variable_name].get(period, dict())
                                                    mapping[variable_name][period][daily_aggregate] = mapping[variable_name][period].get(daily_aggregate,
dict())
                                                    mapping[variable_name][period][daily_aggregate][weekly_aggregate] =
mapping[variable_name][period][daily_aggregate].get(weekly_aggregate, dict())
                                                    mapping[variable_name][period][daily_aggregate][weekly_aggregate][period_aggregate] =
mapping[variable_name][period][daily_aggregate][weekly_aggregate].get(period_aggregate, dict())
                                                    mapping[variable_name][period][daily_aggregate][weekly_aggregate][period_aggregate][str(has_event)] =
mapping[variable_name][period][daily_aggregate][weekly_aggregate][period_aggregate].get(str(has_event), dict())

mapping[variable_name][period][daily_aggregate][weekly_aggregate][period_aggregate][str(has_event)][str(user_id)] = df_event.shape[0]

                        print(json.dumps(mapping, indent=4, sort_keys=True))
```

```
import math

compute_dataset = True

dataset_file_name_flavour_format =
'data/result_data/dataset_{}_period_{}_behaviour_{}_daily_{}_weekly_{}_period_{}_mindaily_{}_minweekly_{}_minperiod_{}_zeros_{}_paired_{}.csv'

if compute_dataset:
```

```
    for minimum_available_data_ratio_daily_active in constraints.minimum_available_data_ratios_daily_active:
        for minimum_available_data_ratio_weekly in constraints.minimum_available_data_ratios_weekly:
            for minimum_available_data_ratio_period in constraints.minimum_available_data_ratios_period:
                if minimum_available_data_ratio_weekly != minimum_available_data_ratio_period:
                    continue

                for allow_zeros in constraints.allows_zeros:
                    for allow_no_event_participants in constraints.allows_no_event_participants:
                        dfs = dict()

                        for user_id in intersection_user_ids:
                            valid_aggregates_user_file =
'data/validation_data/valid_period_aggregates_user_{}_mindaily_{}_minweekly_{}_minperiod_{}_zeros_{}_paired_{}.csv'.format(user_id,
minimum_available_data_ratio_daily_active, minimum_available_data_ratio_weekly, minimum_available_data_ratio_period, allow_zeros,
allow_no_event_participants)

                            if not path.exists(valid_aggregates_user_file):
                                continue

                            print('User: {}'.format(user_id))
                            df_valid_periods_user = pd.read_csv(valid_aggregates_user_file)

                            for behaviour, df_valid_periods_behaviour in df_valid_periods_user.groupby('variable_name'):
                                if behaviour not in variable_definitions.logit_behaviour_target_variable_names:
                                    continue

                                print('Behaviour: {}'.format(behaviour))
                                dfs[behaviour] = dfs.get(behaviour, dict())

                                for period_duration, df_valid_periods_after in df_valid_periods_behaviour.groupby('week_before_after'):
                                    if period_duration not in range(1, 1 + constraints.maximum_weeks_before_after):
                                        continue

                                    for daily_aggregate, df_valid_periods_day_aggregate in df_valid_periods_after.groupby('daily_aggregate'):
                                        print('Daily aggregate: {}'.format(daily_aggregate))
```

```
                                        dfs[behaviour][daily_aggregate] = dfs[behaviour].get(daily_aggregate, dict())

                                        for weekly_aggregate, df_valid_periods_week_aggregate in
df_valid_periods_day_aggregate.groupby('weekly_aggregate'):
                                            print('Weekly aggregate: {}'.format(weekly_aggregate))

                                            dfs[behaviour][daily_aggregate][weekly_aggregate] = dfs[behaviour][daily_aggregate].get(weekly_aggregate,
dict())

                                            for period_aggregate, df_valid_periods_duration in
df_valid_periods_week_aggregate.groupby('period_aggregate'):
                                                print('Period aggregate: {}'.format(period_aggregate))

                                                dfs[behaviour][daily_aggregate][weekly_aggregate][period_aggregate] =
dfs[behaviour][daily_aggregate][weekly_aggregate].get(period_aggregate, dict())

                                                if df_valid_periods_duration.shape[0] <= 0:
                                                    continue

                                                df_valid_periods_positive_user = df_valid_periods_duration[df_valid_periods_duration['has_event'] == True]
                                                df_valid_periods_negative_user = df_valid_periods_duration[df_valid_periods_duration['has_event'] ==
False]


                                                if not allow_no_event_participants:
                                                    if df_valid_periods_positive_user.shape[0] <= 0 or df_valid_periods_negative_user.shape[0] <= 0:
                                                        continue

                                                pd.set_option('mode.chained_assignment', None)
                                                print('Period duration: {}'.format(period_duration))

                                                df_valid_periods_duration['original_date'] = df_valid_periods_duration.apply(lambda row:
days_converter.get_date(row['cardiac_user_date']), axis = 1)
                                                df_valid_periods_duration['week_day'] = df_valid_periods_duration.apply(lambda row:
row['original_date'].isoweekday() , axis = 1)
                                                df_valid_periods_duration['week_day_1'] = df_valid_periods_duration.apply(lambda row: row['week_day'] ==
1, axis = 1)
```

```
                                                df_valid_periods_duration['week_day_2'] = df_valid_periods_duration.apply(lambda row: row['week_day'] ==
2, axis = 1)
                                                df_valid_periods_duration['week_day_3'] = df_valid_periods_duration.apply(lambda row: row['week_day'] ==
3, axis = 1)
                                                df_valid_periods_duration['week_day_4'] = df_valid_periods_duration.apply(lambda row: row['week_day'] ==
4, axis = 1)
                                                df_valid_periods_duration['week_day_5'] = df_valid_periods_duration.apply(lambda row: row['week_day'] ==
5, axis = 1)
                                                df_valid_periods_duration['week_day_6'] = df_valid_periods_duration.apply(lambda row: row['week_day'] ==
6, axis = 1)
                                                df_valid_periods_duration['week_day_7'] = df_valid_periods_duration.apply(lambda row: row['week_day'] ==
7, axis = 1)
                                                df_valid_periods_duration['week_day_working_day'] = df_valid_periods_duration.apply(lambda row: 1 if 1 <=
row['week_day'] and row['week_day'] <= 5 else 0, axis = 1)
                                                df_valid_periods_duration['week_day_weekend_day'] = df_valid_periods_duration.apply(lambda row: 1 if 6 <=
row['week_day'] and row['week_day'] <= 7 else 0, axis = 1)
                                                df_valid_periods_duration['leap_year_bit'] = df_valid_periods_duration.apply(lambda row: 1 if
((row['original_date'].year % 400 == 0) or (row['original_date'].year % 100 != 0) and (row['original_date'].year % 4 == 0)) else 0, axis = 1)
                                                df_valid_periods_duration['original_year'] = df_valid_periods_duration.apply(lambda row:
row['original_date'].year, axis = 1)
                                                df_valid_periods_duration['original_month'] = df_valid_periods_duration.apply(lambda row:
row['original_date'].month, axis = 1)
                                                df_valid_periods_duration['month_1'] = df_valid_periods_duration.apply(lambda row: 1 if
(row['original_month'] == 1) else 0, axis = 1)
                                                df_valid_periods_duration['month_2'] = df_valid_periods_duration.apply(lambda row: 1 if
(row['original_month'] == 2) else 0, axis = 1)
                                                df_valid_periods_duration['month_3'] = df_valid_periods_duration.apply(lambda row: 1 if
(row['original_month'] == 3) else 0, axis = 1)
                                                df_valid_periods_duration['month_4'] = df_valid_periods_duration.apply(lambda row: 1 if
(row['original_month'] == 4) else 0, axis = 1)
                                                df_valid_periods_duration['month_5'] = df_valid_periods_duration.apply(lambda row: 1 if
(row['original_month'] == 5) else 0, axis = 1)
                                                df_valid_periods_duration['month_6'] = df_valid_periods_duration.apply(lambda row: 1 if
(row['original_month'] == 6) else 0, axis = 1)
                                                df_valid_periods_duration['month_7'] = df_valid_periods_duration.apply(lambda row: 1 if
(row['original_month'] == 7) else 0, axis = 1)
```

```
                                              df_valid_periods_duration['month_8'] = df_valid_periods_duration.apply(lambda row: 1 if
(row['original_month'] == 8) else 0, axis = 1)
                                              df_valid_periods_duration['month_9'] = df_valid_periods_duration.apply(lambda row: 1 if
(row['original_month'] == 9) else 0, axis = 1)
                                              df_valid_periods_duration['month_10'] = df_valid_periods_duration.apply(lambda row: 1 if
(row['original_month'] == 10) else 0, axis = 1)
                                              df_valid_periods_duration['month_11'] = df_valid_periods_duration.apply(lambda row: 1 if
(row['original_month'] == 11) else 0, axis = 1)
                                              df_valid_periods_duration['month_12'] = df_valid_periods_duration.apply(lambda row: 1 if
(row['original_month'] == 12) else 0, axis = 1)

                                              for diff_year in range(2018, 2021):
                                                  for diff_month in range(1, 13):
                                                      def month_diff(this_year, this_month, reference_year, reference_month):
                                                          return (this_year - reference_year) * 12 + this_month - reference_month

                                                      index = (diff_year - 2018) * 12 + diff_month
                                                      index_str = 'month_{}_normal'.format(index)
                                                      df_valid_periods_duration[index_str] = df_valid_periods_duration.apply(lambda row: math.pow(0.5,
abs(month_diff(row['original_year'], row['original_month'], diff_year, diff_month)) * 1.0), axis = 1)

                                              df_valid_periods_duration['season_winter'] = df_valid_periods_duration.apply(lambda row: 1 if (12 <=
row['original_month'] or row['original_month'] < 3) else 0, axis = 1)
                                              df_valid_periods_duration['season_spring'] = df_valid_periods_duration.apply(lambda row: 1 if (3 <=
row['original_month'] and row['original_month'] < 6) else 0, axis = 1)
                                              df_valid_periods_duration['season_summer'] = df_valid_periods_duration.apply(lambda row: 1 if (6 <=
row['original_month'] and row['original_month'] < 9) else 0, axis = 1)
                                              df_valid_periods_duration['season_fall'] = df_valid_periods_duration.apply(lambda row: 1 if (9 <=
row['original_month'] and row['original_month'] < 12) else 0, axis = 1)
                                              df_valid_periods_duration['season'] = df_valid_periods_duration.apply(lambda row: row['season_winter'] + 2
* row['season_spring'] + 3 * row['season_summer'] + 4 * row['season_fall'], axis = 1)
                                              df_valid_periods_duration['year_2018'] = df_valid_periods_duration.apply(lambda row: 1 if
(row['original_year'] == 2018) else 0, axis = 1)
                                              df_valid_periods_duration['year_2019'] = df_valid_periods_duration.apply(lambda row: 1 if
(row['original_year'] == 2019) else 0, axis = 1)
                                              df_valid_periods_duration['year_2020'] = df_valid_periods_duration.apply(lambda row: 1 if
```

```
(row['original_year'] == 2020) else 0, axis = 1)
                                              df_valid_periods_duration['day_of_year'] = df_valid_periods_duration.apply(lambda row:
row['original_date'].timetuple().tm_yday, axis = 1)
                                              df_valid_periods_duration['week_of_year'] = df_valid_periods_duration.apply(lambda row: row['day_of_year']
// 7, axis = 1)
                                              df_valid_periods_duration['month_of_year'] = df_valid_periods_duration.apply(lambda row:
row['original_date'].month, axis = 1)


                                              df_valid_periods_duration['value_{}'.format(behaviour)] = df_valid_periods_duration['value']

                                              df_final = dfs[behaviour][daily_aggregate][weekly_aggregate][period_aggregate].get(period_duration, None)

                                              if df_final is None:
                                                  df_final = df_valid_periods_duration
                                              else:
                                                  df_final = pd.concat([df_final, df_valid_periods_duration])

                                              dfs[behaviour][daily_aggregate][weekly_aggregate][period_aggregate][period_duration] = df_final

                      for behaviour in variable_definitions.logit_behaviour_target_variable_names:
                          if behaviour not in dfs:
                              continue

                          print('Behaviour: {}'.format(behaviour))

                          for daily_aggregate in aggregates:
                              if daily_aggregate not in dfs[behaviour]:
                                  continue

                              print('Daily aggregate: {}'.format(daily_aggregate))

                              for weekly_aggregate in aggregates:
                                  if weekly_aggregate not in dfs[behaviour][daily_aggregate]:
                                      continue

                                  print('Weekly aggregate: {}'.format(weekly_aggregate))
```

```
for period_aggregate in aggregates:
    if period_aggregate not in dfs[behaviour][daily_aggregate][weekly_aggregate]:
        continue

    print('Period aggregate: {}'.format(period_aggregate))

    df_combined = dict()

    for period_duration in range(1, 1 + constraints.maximum_weeks_before_after):
        if period_duration not in dfs[behaviour][daily_aggregate][weekly_aggregate][period_aggregate]:
            continue

        print('Period duration: {}'.format(period_duration))
        df = dfs[behaviour][daily_aggregate][weekly_aggregate][period_aggregate][period_duration]

        for other_period_duration in range(period_duration, 1 + constraints.maximum_weeks_before_after):
            df_combined_other = df_combined.get(other_period_duration, None)

            if df_combined_other is None:
                df_combined_other = df
            else:
                df_combined_other = pd.concat([df_combined_other, df])

            df_combined[other_period_duration] = df_combined_other

        dataset_file_name = dataset_file_name_flavour_format.format('separate', period_duration, behaviour,
daily_aggregate, weekly_aggregate, period_aggregate, minimum_available_data_ratio_daily_active, minimum_available_data_ratio_weekly,
minimum_available_data_ratio_period, allow_zeros, allow_no_event_participants)

        if not path.exists(dataset_file_name):
            df.to_csv(dataset_file_name)

    for other_period_duration in range(1, 1 + constraints.maximum_weeks_before_after):
        if other_period_duration not in df_combined:
            continue
```

```
                                           dataset_combined_file_name = dataset_file_name_flavour_format.format('cumulative', other_period_duration,
behaviour, daily_aggregate, weekly_aggregate, period_aggregate, minimum_available_data_ratio_daily_active, minimum_available_data_ratio_weekly,
minimum_available_data_ratio_period, allow_zeros, allow_no_event_participants)

                                           if not path.exists(dataset_combined_file_name):
                                               df_combined[other_period_duration].to_csv(dataset_combined_file_name)
```

sleep_parser.py

```
import pandas as pd

class sleep_parser:
   seconds_in_minute = 60
   other_periodicity = 15

   @staticmethod
   def parse(df, id_column, minutes_column, sleep_stage_column, duration_column):

       # User ID, minute, sleep stage (true)
       mapping = dict()
       user_groups = df.groupby(id_column)

       for user_id, user_group in user_groups:
           mapping[user_id] = mapping.get(user_id, dict())
           minutes_groups = user_group.groupby(minutes_column)

           for minutes_start, minutes_group in minutes_groups:
               for idx, row in minutes_group.iterrows():
                   duration = row[duration_column]
                   sleep_stage_id = row[sleep_stage_column]
                   minutes_end = minutes_start + (duration // sleep_parser.seconds_in_minute)

                   for minutes_numeric in range(minutes_start, minutes_end):
                       minutes_numeric_div = (minutes_numeric // sleep_parser.other_periodicity) * sleep_parser.other_periodicity
                       mapping[user_id][minutes_numeric_div] = mapping[user_id].get(minutes_numeric_div, dict())
```

```
                mapping[user_id][minutes_numeric_div][sleep_stage_id] = mapping[user_id][minutes_numeric_div].get(sleep_stage_id, 0)
                mapping[user_id][minutes_numeric_div][sleep_stage_id] = 1 + mapping[user_id][minutes_numeric_div][sleep_stage_id]

                # Check to not have more of the stage filled than available minutes.
                assert mapping[user_id][minutes_numeric_div][sleep_stage_id] <= sleep_parser.other_periodicity

    sleep_stage_ids = df[sleep_stage_column].unique()
    entries = []

    for user_id, user_group in mapping.items():
        for minute_numeric, sleep_stage_ids_group in user_group.items():
            entry = [user_id, minute_numeric]
            sum = 0

            for sleep_stage_id in sleep_stage_ids:
                if sleep_stage_id in sleep_stage_ids_group:
                    value = sleep_stage_ids_group[sleep_stage_id]
                    entry.append(value)
                    sum += value
                else:
                    entry.append(0)

            # Check to not have more stages filled than available minutes.
            assert sum <= sleep_parser.other_periodicity

            entries.append(entry)

    sleep_stage_columns = ['{}__{}'.format(sleep_stage_column, sleep_stage_id) for sleep_stage_id in sleep_stage_ids]
    df_parsed = pd.DataFrame(entries, columns = [id_column, minutes_column] + sleep_stage_columns)
    return df_parsed
```

variable_definitions.py

```
import numpy as np

class variable_definitions:
```

```
behaviour_target_variable_names = [
    'StepsCount',

    'ActivityTimeSedentary',
    'ActivityTimeLight',
    'ActivityTimeFair',
    'ActivityTimeVigorous',
    'ActivityTimeSedentaryLight',
    'ActivityTimeLightFair',
    'ActivityTimeFairVigorous',
    'ActivityTimeActive',
    'ActivityTimeTotal',

    'SleepTimeAsleep',
    'SleepTimeAwake',
    'SleepTimeRestless',
    'SleepTimeAsleepAwake',
    'SleepTimeAwakeRestless',
    'SleepTimeAsleepRestless',
    'SleepTimeTotal',
    'SleepTimeTotalKnown',

    'ActivityTimeSedentaryAwake',
    'ActivityTimeSedentaryLightAwake',

    'HeartRateMeanSedentary',
    'HeartRateMedianSedentary',
    'HeartRateMinSedentary',
    'HeartRateMaxSedentary',
    'HeartRateStdevSedentary',

    'HeartRateMeanSedentaryAwake',
    'HeartRateMedianSedentaryAwake',
    'HeartRateMinSedentaryAwake',
    'HeartRateMaxSedentaryAwake',
    'HeartRateStdevSedentaryAwake',
```

```
    'HeartRateMeanLight',
    'HeartRateMedianLight',
    'HeartRateMinLight',
    'HeartRateMaxLight',
    'HeartRateStdevLight',

    'HeartRateMeanFair',
    'HeartRateMedianFair',
    'HeartRateMinFair',
    'HeartRateMaxFair',
    'HeartRateStdevFair',

    'HeartRateMeanVigorous',
    'HeartRateMedianVigorous',
    'HeartRateMinVigorous',
    'HeartRateMaxVigorous',
    'HeartRateStdevVigorous',

    'HeartRateMeanSedentaryLight',
    'HeartRateMedianSedentaryLight',
    'HeartRateMinSedentaryLight',
    'HeartRateMaxSedentaryLight',
    'HeartRateStdevSedentaryLight',

    'HeartRateMeanSedentaryLightAwake',
    'HeartRateMedianSedentaryLightAwake',
    'HeartRateMinSedentaryLightAwake',
    'HeartRateMaxSedentaryLightAwake',
    'HeartRateStdevSedentaryLightAwake',

    'HeartRateMeanLightFair',
    'HeartRateMedianLightFair',
    'HeartRateMinLightFair',
    'HeartRateMaxLightFair',
    'HeartRateStdevLightFair',
```

```
    'HeartRateMeanFairVigorous',
    'HeartRateMedianFairVigorous',
    'HeartRateMinFairVigorous',
    'HeartRateMaxFairVigorous',
    'HeartRateStdevFairVigorous',

    'HeartRateMeanActive',
    'HeartRateMedianActive',
    'HeartRateMinActive',
    'HeartRateMaxActive',
    'HeartRateStdevActive',

    'HeartRateMeanAsleep',
    'HeartRateMedianAsleep',
    'HeartRateMinAsleep',
    'HeartRateMaxAsleep',
    'HeartRateStdevAsleep',

    'HeartRateMeanAwake',
    'HeartRateMedianAwake',
    'HeartRateMinAwake',
    'HeartRateMaxAwake',
    'HeartRateStdevAwake',

    'HeartRateMeanRestless',
    'HeartRateMedianRestless',
    'HeartRateMinRestless',
    'HeartRateMaxRestless',
    'HeartRateStdevRestless',

    'HeartRateMeanAsleepAwake',
    'HeartRateMedianAsleepAwake',
    'HeartRateMinAsleepAwake',
    'HeartRateMaxAsleepAwake',
    'HeartRateStdevAsleepAwake',
```

```
    'HeartRateMeanAwakeRestless',
    'HeartRateMedianAwakeRestless',
    'HeartRateMinAwakeRestless',
    'HeartRateMaxAwakeRestless',
    'HeartRateStdevAwakeRestless',

    'HeartRateMeanAsleepRestless',
    'HeartRateMedianAsleepRestless',
    'HeartRateMinAsleepRestless',
    'HeartRateMaxAsleepRestless',
    'HeartRateStdevAsleepRestless',

    'HeartRateMeanSleeping',
    'HeartRateMedianSleeping',
    'HeartRateMinSleeping',
    'HeartRateMaxSleeping',
    'HeartRateStdevSleeping',

    'HeartRateMean',
    'HeartRateMedian',
    'HeartRateMin',
    'HeartRateMax',
    'HeartRateStdev',

    '''
    'SleepTimeAsleep',
    'SleepTimeAwake',
    'SleepTimeRestless',
    'SleepTimeAsleepAwake',
    'SleepTimeAwakeRestless',
    'SleepTimeAsleepRestless',
    'SleepTimeTotal',
    'SleepTimeTotalKnown',
    '''
]
```

```
    event_target_variable_names = [
        'AverageVentricularRate',
        'MaximumVentricularRate',
        'Duration'
    ]

    logit_behaviour_target_variable_names = [
        #'StepsCount',
        #'ActivityTimeSedentaryAwake',
        #'ActivityTimeSedentaryLightAwake',
        #'ActivityTimeLight',
        #'ActivityTimeLightFair',
        #'ActivityTimeFair',
        #'ActivityTimeFairVigorous',
        #'ActivityTimeVigorous',
        'SleepTimeAsleep',
        'SleepTimeAwake',
        'SleepTimeRestless',
        'SleepTimeAsleepAwake',
        'SleepTimeAwakeRestless',
        'SleepTimeAsleepRestless',
        #'SleepTimeTotal',
        #'HeartRateMean',
        #'HeartRateMedian',
        #'HeartRateMin',
        #'HeartRateMax',
        #'HeartRateStdev'
    ]

    definitions = [
        {
            'ActivityTimeTotal': lambda row, data_frequency_minutes: variable_definitions.calculate_union_frequency(row, ['ActivityLevel_Id__1',
'ActivityLevel_Id__2', 'ActivityLevel_Id__3', 'ActivityLevel_Id__4'], data_frequency_minutes),

            'ActivityTimeSedentary': lambda row, data_frequency_minutes: variable_definitions.calculate_union_frequency(row, ['ActivityLevel_Id__1'],
```

```
data_frequency_minutes),

            'ActivityTimeLight': lambda row, data_frequency_minutes: variable_definitions.calculate_union_frequency(row, ['ActivityLevel_Id__2'],
data_frequency_minutes),

            'ActivityTimeFair': lambda row, data_frequency_minutes: variable_definitions.calculate_union_frequency(row, ['ActivityLevel_Id__3'],
data_frequency_minutes),

            'ActivityTimeVigorous': lambda row, data_frequency_minutes: variable_definitions.calculate_union_frequency(row, ['ActivityLevel_Id__4'],
data_frequency_minutes),

            'ActivityTimeSedentaryLight': lambda row, data_frequency_minutes: variable_definitions.calculate_union_frequency(row, ['ActivityLevel_Id__1',
'ActivityLevel_Id__2'], data_frequency_minutes),

            'ActivityTimeLightFair': lambda row, data_frequency_minutes: variable_definitions.calculate_union_frequency(row, ['ActivityLevel_Id__2',
'ActivityLevel_Id__3'], data_frequency_minutes),

            'ActivityTimeFairVigorous': lambda row, data_frequency_minutes: variable_definitions.calculate_union_frequency(row, ['ActivityLevel_Id__3',
'ActivityLevel_Id__4'], data_frequency_minutes),

            'ActivityTimeActive': lambda row, data_frequency_minutes: variable_definitions.calculate_union_frequency(row, ['ActivityLevel_Id__2',
'ActivityLevel_Id__3', 'ActivityLevel_Id__4'], data_frequency_minutes),

            'SleepTimeAsleep': lambda row, data_frequency_minutes: variable_definitions.calculate_union(row, ['SleepStage_Id__0']),

            'SleepTimeAwake': lambda row, data_frequency_minutes: variable_definitions.calculate_union(row, ['SleepStage_Id__1']),

            'SleepTimeRestless': lambda row, data_frequency_minutes: variable_definitions.calculate_union(row, ['SleepStage_Id__2']),

            'SleepTimeAsleepAwake': lambda row, data_frequency_minutes: variable_definitions.calculate_union(row, ['SleepStage_Id__0',
'SleepStage_Id__1']),

            'SleepTimeAwakeRestless': lambda row, data_frequency_minutes: variable_definitions.calculate_union(row, ['SleepStage_Id__1',
'SleepStage_Id__2']),

            'SleepTimeAsleepRestless': lambda row, data_frequency_minutes: variable_definitions.calculate_union(row, ['SleepStage_Id__0',
```

```
'SleepStage_Id__2']),

            'SleepTimeTotalKnown': lambda row, data_frequency_minutes: variable_definitions.calculate_union(row, ['SleepStage_Id__0', 'SleepStage_Id__1', 
'SleepStage_Id__2']),

            'SleepTimeTotal': lambda row, data_frequency_minutes: variable_definitions.calculate_union(row, ['SleepStage_Id__0', 'SleepStage_Id__1', 
'SleepStage_Id__2', 'SleepStage_Id__8'])
        },
        {
            'DailyTimeTotal': lambda row, data_frequency_minutes: variable_definitions.calculate_union(row, ['SleepTimeTotal', 'ActivityTimeTotal']),

            'ActivityTimeSedentaryAwake': lambda row, data_frequency_minutes: max(0, row['ActivityTimeSedentary'] - row['SleepTimeTotal']),

            'ActivityTimeSedentaryLightAwake': lambda row, data_frequency_minutes: max(0, row['ActivityTimeSedentaryLight'] - row['SleepTimeTotal']),
        },
        {
            'HeartRateMeanSedentary': lambda row, data_frequency_minutes: variable_definitions.calculate_union_conditional(row, ['HeartRateMean'], 
'ActivityTimeSedentary'),
            'HeartRateMedianSedentary': lambda row, data_frequency_minutes: variable_definitions.calculate_union_conditional(row, ['HeartRateMedian'], 
'ActivityTimeSedentary'),
            'HeartRateMinSedentary': lambda row, data_frequency_minutes: variable_definitions.calculate_union_conditional(row, ['HeartRateMin'], 
'ActivityTimeSedentary'),
            'HeartRateMaxSedentary': lambda row, data_frequency_minutes: variable_definitions.calculate_union_conditional(row, ['HeartRateMax'], 
'ActivityTimeSedentary'),
            'HeartRateStdevSedentary': lambda row, data_frequency_minutes: variable_definitions.calculate_union_conditional(row, ['HeartRateStdev'], 
'ActivityTimeSedentary'),

            'HeartRateMeanSedentaryAwake': lambda row, data_frequency_minutes: variable_definitions.calculate_union_conditional(row, ['HeartRateMean'], 
'ActivityTimeSedentaryAwake'),
            'HeartRateMedianSedentaryAwake': lambda row, data_frequency_minutes: variable_definitions.calculate_union_conditional(row, 
['HeartRateMedian'], 'ActivityTimeSedentaryAwake'),
            'HeartRateMinSedentaryAwake': lambda row, data_frequency_minutes: variable_definitions.calculate_union_conditional(row, ['HeartRateMin'], 
'ActivityTimeSedentaryAwake'),
            'HeartRateMaxSedentaryAwake': lambda row, data_frequency_minutes: variable_definitions.calculate_union_conditional(row, ['HeartRateMax'], 
'ActivityTimeSedentaryAwake'),
            'HeartRateStdevSedentaryAwake': lambda row, data_frequency_minutes: variable_definitions.calculate_union_conditional(row, ['HeartRateStdev'], 
```

```
'ActivityTimeSedentaryAwake'),

           'HeartRateMeanLight': lambda row, data_frequency_minutes: variable_definitions.calculate_union_conditional(row, ['HeartRateMean'],
'ActivityTimeLight'),
           'HeartRateMedianLight': lambda row, data_frequency_minutes: variable_definitions.calculate_union_conditional(row, ['HeartRateMedian'],
'ActivityTimeLight'),
           'HeartRateMinLight': lambda row, data_frequency_minutes: variable_definitions.calculate_union_conditional(row, ['HeartRateMin'],
'ActivityTimeLight'),
           'HeartRateMaxLight': lambda row, data_frequency_minutes: variable_definitions.calculate_union_conditional(row, ['HeartRateMax'],
'ActivityTimeLight'),
           'HeartRateStdevLight': lambda row, data_frequency_minutes: variable_definitions.calculate_union_conditional(row, ['HeartRateStdev'],
'ActivityTimeLight'),

           'HeartRateMeanFair': lambda row, data_frequency_minutes: variable_definitions.calculate_union_conditional(row, ['HeartRateMean'],
'ActivityTimeFair'),
           'HeartRateMedianFair': lambda row, data_frequency_minutes: variable_definitions.calculate_union_conditional(row, ['HeartRateMedian'],
'ActivityTimeFair'),
           'HeartRateMinFair': lambda row, data_frequency_minutes: variable_definitions.calculate_union_conditional(row, ['HeartRateMin'],
'ActivityTimeFair'),
           'HeartRateMaxFair': lambda row, data_frequency_minutes: variable_definitions.calculate_union_conditional(row, ['HeartRateMax'],
'ActivityTimeFair'),
           'HeartRateStdevFair': lambda row, data_frequency_minutes: variable_definitions.calculate_union_conditional(row, ['HeartRateStdev'],
'ActivityTimeFair'),

           'HeartRateMeanVigorous': lambda row, data_frequency_minutes: variable_definitions.calculate_union_conditional(row, ['HeartRateMean'],
'ActivityTimeVigorous'),
           'HeartRateMedianVigorous': lambda row, data_frequency_minutes: variable_definitions.calculate_union_conditional(row, ['HeartRateMedian'],
'ActivityTimeVigorous'),
           'HeartRateMinVigorous': lambda row, data_frequency_minutes: variable_definitions.calculate_union_conditional(row, ['HeartRateMin'],
'ActivityTimeVigorous'),
           'HeartRateMaxVigorous': lambda row, data_frequency_minutes: variable_definitions.calculate_union_conditional(row, ['HeartRateMax'],
'ActivityTimeVigorous'),
           'HeartRateStdevVigorous': lambda row, data_frequency_minutes: variable_definitions.calculate_union_conditional(row, ['HeartRateStdev'],
'ActivityTimeVigorous'),

           'HeartRateMeanSedentaryLight': lambda row, data_frequency_minutes: variable_definitions.calculate_union_conditional(row, ['HeartRateMean'],
```

```
'ActivityTimeSedentaryLight'),
            'HeartRateMedianSedentaryLight': lambda row, data_frequency_minutes: variable_definitions.calculate_union_conditional(row,
['HeartRateMedian'], 'ActivityTimeSedentaryLight'),
            'HeartRateMinSedentaryLight': lambda row, data_frequency_minutes: variable_definitions.calculate_union_conditional(row, ['HeartRateMin'],
'ActivityTimeSedentaryLight'),
            'HeartRateMaxSedentaryLight': lambda row, data_frequency_minutes: variable_definitions.calculate_union_conditional(row, ['HeartRateMax'],
'ActivityTimeSedentaryLight'),
            'HeartRateStdevSedentaryLight': lambda row, data_frequency_minutes: variable_definitions.calculate_union_conditional(row, ['HeartRateStdev'],
'ActivityTimeSedentaryLight'),

            'HeartRateMeanSedentaryLightAwake': lambda row, data_frequency_minutes: variable_definitions.calculate_union_conditional(row,
['HeartRateMean'], 'ActivityTimeSedentaryLightAwake'),
            'HeartRateMedianSedentaryLightAwake': lambda row, data_frequency_minutes: variable_definitions.calculate_union_conditional(row,
['HeartRateMedian'], 'ActivityTimeSedentaryLightAwake'),
            'HeartRateMinSedentaryLightAwake': lambda row, data_frequency_minutes: variable_definitions.calculate_union_conditional(row, ['HeartRateMin'],
'ActivityTimeSedentaryLightAwake'),
            'HeartRateMaxSedentaryLightAwake': lambda row, data_frequency_minutes: variable_definitions.calculate_union_conditional(row, ['HeartRateMax'],
'ActivityTimeSedentaryLightAwake'),
            'HeartRateStdevSedentaryLightAwake': lambda row, data_frequency_minutes: variable_definitions.calculate_union_conditional(row,
['HeartRateStdev'], 'ActivityTimeSedentaryLightAwake'),

            'HeartRateMeanLightFair': lambda row, data_frequency_minutes: variable_definitions.calculate_union_conditional(row, ['HeartRateMean'],
'ActivityTimeLightFair'),
            'HeartRateMedianLightFair': lambda row, data_frequency_minutes: variable_definitions.calculate_union_conditional(row, ['HeartRateMedian'],
'ActivityTimeLightFair'),
            'HeartRateMinLightFair': lambda row, data_frequency_minutes: variable_definitions.calculate_union_conditional(row, ['HeartRateMin'],
'ActivityTimeLightFair'),
            'HeartRateMaxLightFair': lambda row, data_frequency_minutes: variable_definitions.calculate_union_conditional(row, ['HeartRateMax'],
'ActivityTimeLightFair'),
            'HeartRateStdevLightFair': lambda row, data_frequency_minutes: variable_definitions.calculate_union_conditional(row, ['HeartRateStdev'],
'ActivityTimeLightFair'),

            'HeartRateMeanFairVigorous': lambda row, data_frequency_minutes: variable_definitions.calculate_union_conditional(row, ['HeartRateMean'],
'ActivityTimeFairVigorous'),
            'HeartRateMedianFairVigorous': lambda row, data_frequency_minutes: variable_definitions.calculate_union_conditional(row, ['HeartRateMedian'],
'ActivityTimeFairVigorous'),
```

```
            'HeartRateMinFairVigorous': lambda row, data_frequency_minutes: variable_definitions.calculate_union_conditional(row, ['HeartRateMin'],
'ActivityTimeFairVigorous'),
            'HeartRateMaxFairVigorous': lambda row, data_frequency_minutes: variable_definitions.calculate_union_conditional(row, ['HeartRateMax'],
'ActivityTimeFairVigorous'),
            'HeartRateStdevFairVigorous': lambda row, data_frequency_minutes: variable_definitions.calculate_union_conditional(row, ['HeartRateStdev'],
'ActivityTimeFairVigorous'),

            'HeartRateMeanActive': lambda row, data_frequency_minutes: variable_definitions.calculate_union_conditional(row, ['HeartRateMean'],
'ActivityTimeActive'),
            'HeartRateMedianActive': lambda row, data_frequency_minutes: variable_definitions.calculate_union_conditional(row, ['HeartRateMedian'],
'ActivityTimeActive'),
            'HeartRateMinActive': lambda row, data_frequency_minutes: variable_definitions.calculate_union_conditional(row, ['HeartRateMin'],
'ActivityTimeActive'),
            'HeartRateMaxActive': lambda row, data_frequency_minutes: variable_definitions.calculate_union_conditional(row, ['HeartRateMax'],
'ActivityTimeActive'),
            'HeartRateStdevActive': lambda row, data_frequency_minutes: variable_definitions.calculate_union_conditional(row, ['HeartRateStdev'],
'ActivityTimeActive'),

            'HeartRateMeanAsleep': lambda row, data_frequency_minutes: variable_definitions.calculate_union_conditional(row, ['HeartRateMean'],
'SleepTimeAsleep'),
            'HeartRateMedianAsleep': lambda row, data_frequency_minutes: variable_definitions.calculate_union_conditional(row, ['HeartRateMedian'],
'SleepTimeAsleep'),
            'HeartRateMinAsleep': lambda row, data_frequency_minutes: variable_definitions.calculate_union_conditional(row, ['HeartRateMin'],
'SleepTimeAsleep'),
            'HeartRateMaxAsleep': lambda row, data_frequency_minutes: variable_definitions.calculate_union_conditional(row, ['HeartRateMax'],
'SleepTimeAsleep'),
            'HeartRateStdevAsleep': lambda row, data_frequency_minutes: variable_definitions.calculate_union_conditional(row, ['HeartRateStdev'],
'SleepTimeAsleep'),

            'HeartRateMeanAwake': lambda row, data_frequency_minutes: variable_definitions.calculate_union_conditional(row, ['HeartRateMean'],
'SleepTimeAwake'),
            'HeartRateMedianAwake': lambda row, data_frequency_minutes: variable_definitions.calculate_union_conditional(row, ['HeartRateMedian'],
'SleepTimeAwake'),
            'HeartRateMinAwake': lambda row, data_frequency_minutes: variable_definitions.calculate_union_conditional(row, ['HeartRateMin'],
'SleepTimeAwake'),
            'HeartRateMaxAwake': lambda row, data_frequency_minutes: variable_definitions.calculate_union_conditional(row, ['HeartRateMax'],
```

```
'SleepTimeAwake'),
            'HeartRateStdevAwake': lambda row, data_frequency_minutes: variable_definitions.calculate_union_conditional(row, ['HeartRateStdev'],
'SleepTimeAwake'),

            'HeartRateMeanRestless': lambda row, data_frequency_minutes: variable_definitions.calculate_union_conditional(row, ['HeartRateMean'],
'SleepTimeRestless'),
            'HeartRateMedianRestless': lambda row, data_frequency_minutes: variable_definitions.calculate_union_conditional(row, ['HeartRateMedian'],
'SleepTimeRestless'),
            'HeartRateMinRestless': lambda row, data_frequency_minutes: variable_definitions.calculate_union_conditional(row, ['HeartRateMin'],
'SleepTimeRestless'),
            'HeartRateMaxRestless': lambda row, data_frequency_minutes: variable_definitions.calculate_union_conditional(row, ['HeartRateMax'],
'SleepTimeRestless'),
            'HeartRateStdevRestless': lambda row, data_frequency_minutes: variable_definitions.calculate_union_conditional(row, ['HeartRateStdev'],
'SleepTimeRestless'),

            'HeartRateMeanAsleepAwake': lambda row, data_frequency_minutes: variable_definitions.calculate_union_conditional(row, ['HeartRateMean'],
'SleepTimeAsleepAwake'),
            'HeartRateMedianAsleepAwake': lambda row, data_frequency_minutes: variable_definitions.calculate_union_conditional(row, ['HeartRateMedian'],
'SleepTimeAsleepAwake'),
            'HeartRateMinAsleepAwake': lambda row, data_frequency_minutes: variable_definitions.calculate_union_conditional(row, ['HeartRateMin'],
'SleepTimeAsleepAwake'),
            'HeartRateMaxAsleepAwake': lambda row, data_frequency_minutes: variable_definitions.calculate_union_conditional(row, ['HeartRateMax'],
'SleepTimeAsleepAwake'),
            'HeartRateStdevAsleepAwake': lambda row, data_frequency_minutes: variable_definitions.calculate_union_conditional(row, ['HeartRateStdev'],
'SleepTimeAsleepAwake'),

            'HeartRateMeanAwakeRestless': lambda row, data_frequency_minutes: variable_definitions.calculate_union_conditional(row, ['HeartRateMean'],
'SleepTimeAwakeRestless'),
            'HeartRateMedianAwakeRestless': lambda row, data_frequency_minutes: variable_definitions.calculate_union_conditional(row, ['HeartRateMedian'],
'SleepTimeAwakeRestless'),
            'HeartRateMinAwakeRestless': lambda row, data_frequency_minutes: variable_definitions.calculate_union_conditional(row, ['HeartRateMin'],
'SleepTimeAwakeRestless'),
            'HeartRateMaxAwakeRestless': lambda row, data_frequency_minutes: variable_definitions.calculate_union_conditional(row, ['HeartRateMax'],
'SleepTimeAwakeRestless'),
            'HeartRateStdevAwakeRestless': lambda row, data_frequency_minutes: variable_definitions.calculate_union_conditional(row, ['HeartRateStdev'],
'SleepTimeAwakeRestless'),
```

```
            'HeartRateMeanAsleepRestless': lambda row, data_frequency_minutes: variable_definitions.calculate_union_conditional(row, ['HeartRateMean'],
'SleepTimeAsleepRestless'),
            'HeartRateMedianAsleepRestless': lambda row, data_frequency_minutes: variable_definitions.calculate_union_conditional(row,
['HeartRateMedian'], 'SleepTimeAsleepRestless'),
            'HeartRateMinAsleepRestless': lambda row, data_frequency_minutes: variable_definitions.calculate_union_conditional(row, ['HeartRateMin'],
'SleepTimeAsleepRestless'),
            'HeartRateMaxAsleepRestless': lambda row, data_frequency_minutes: variable_definitions.calculate_union_conditional(row, ['HeartRateMax'],
'SleepTimeAsleepRestless'),
            'HeartRateStdevAsleepRestless': lambda row, data_frequency_minutes: variable_definitions.calculate_union_conditional(row, ['HeartRateStdev'],
'SleepTimeAsleepRestless'),

            'HeartRateMeanSleeping': lambda row, data_frequency_minutes: variable_definitions.calculate_union_conditional(row, ['HeartRateMean'],
'SleepTimeTotal'),
            'HeartRateMedianSleeping': lambda row, data_frequency_minutes: variable_definitions.calculate_union_conditional(row, ['HeartRateMedian'],
'SleepTimeTotal'),
            'HeartRateMinSleeping': lambda row, data_frequency_minutes: variable_definitions.calculate_union_conditional(row, ['HeartRateMin'],
'SleepTimeTotal'),
            'HeartRateMaxSleeping': lambda row, data_frequency_minutes: variable_definitions.calculate_union_conditional(row, ['HeartRateMax'],
'SleepTimeTotal'),
            'HeartRateStdevSleeping': lambda row, data_frequency_minutes: variable_definitions.calculate_union_conditional(row, ['HeartRateStdev'],
'SleepTimeTotal'),

            'HeartRateMean': lambda row, data_frequency_minutes: variable_definitions.calculate_union_conditional(row, ['HeartRateMean'],
'ActivityTimeTotal'),
            'HeartRateMedian': lambda row, data_frequency_minutes: variable_definitions.calculate_union_conditional(row, ['HeartRateMedian'],
'ActivityTimeTotal'),
            'HeartRateMin': lambda row, data_frequency_minutes: variable_definitions.calculate_union_conditional(row, ['HeartRateMin'],
'ActivityTimeTotal'),
            'HeartRateMax': lambda row, data_frequency_minutes: variable_definitions.calculate_union_conditional(row, ['HeartRateMax'],
'ActivityTimeTotal'),
            'HeartRateStdev': lambda row, data_frequency_minutes: variable_definitions.calculate_union_conditional(row, ['HeartRateStdev'],
'ActivityTimeTotal'),
        }
    ]
```

```
    @staticmethod
    def calculate_union(row, columns):
        found = False
        summation = 0.0

        for column in columns:
            if column in row:
                if (row[column] is not None) and (not np.isnan(row[column])):
                    summation = summation + row[column]
                    found = True

        if found:
            return summation

        return np.nan

    @staticmethod
    def calculate_union_frequency(row, columns, frequency):
        result = variable_definitions.calculate_union(row, columns)

        if result is None:
            return np.nan

        return result * frequency

    @staticmethod
    def calculate_union_conditional(row, columns, column_condition):
        if (row[column_condition] is None) or np.isnan(row[column_condition]):
            return np.nan

        return variable_definitions.calculate_union(row, columns)
```

variable_derivator.py

```
from variable_definitions import variable_definitions
from variable_properties import variable_properties
```

```python
import numpy as np

class variable_derivator:
   def __init__(self, date_numeric_column):
       self.date_numeric_column = date_numeric_column

   def derive(self, df_duration):
       print('derive')
       data_frequency_minutes = variable_properties.data_frequency_minutes

       for variable_definition_stage in variable_definitions.definitions:
           for variable_name, definition in variable_definition_stage.items():
               df_duration[variable_name] = df_duration.apply(lambda row: definition(row, data_frequency_minutes), axis = 1)
               print('Derived {}'.format(variable_name))

       print('derive finished')
       return df_duration

   @staticmethod
   def calculate_union(row, columns):
       summation = 0

       for column in columns:
           if column in row:
               if not np.isnan(row[column]):
                   summation = summation + row[column]

       return summation

   @staticmethod
   def calculate_union_conditional(row, columns, column_condition):
       summation = 0

       if np.isnan(row[column_condition]):
           return summation
```

```
        for column in columns:
            if column in row:
                if not np.isnan(row[column]):
                    summation = summation + row[column]

        return summation
```

variable_properties.py

```
import numpy as np

class variable_properties:
    behaviours_aggregates = {
        'steps': {
            'StepsCount': np.sum
        },
        'activity': {
            'ActivityTimeSedentaryAwake': np.sum,
            'ActivityTimeSedentaryLightAwake': np.sum,
            'ActivityTimeLight': np.sum,
            'ActivityTimeLightFair': np.sum,
            'ActivityTimeFair': np.sum,
            'ActivityTimeFairVigorous': np.sum,
            'ActivityTimeVigorous': np.sum,
            'ActivityTimeActive': np.sum,
            'SleepTimeTotal': np.sum
        },
        'sleep': {
            'SleepTimeAsleep': np.sum,
            'SleepTimeAwake': np.sum,
            'SleepTimeRestless': np.sum,
            'SleepTimeAsleepAwake': np.sum,
            'SleepTimeAwakeRestless': np.sum,
            'SleepTimeAsleepRestless': np.sum,
            'SleepTimeTotal': np.sum
```

```
    },
    'heart': {
        'HeartRateMin': np.mean,
        'HeartRateMean': np.mean,
        'HeartRateMedian': np.mean,
        'HeartRateMax': np.mean,
        'HeartRateStdev': np.mean
    }
}

data_frequency_minutes = 15

event_variable_aggregates = {
    'AverageVentricularRate': np.mean,
    'MaximumVentricularRate': np.max,
    'Duration': np.max
}

target_column_period_aggregation_functions = {
    'StepsCount': {'mean': np.mean},
    'ActivityTimeSedentaryAwake': {'mean': np.mean},
    'ActivityTimeSedentaryLightAwake': {'mean': np.mean},
    'ActivityTimeLight': {'mean': np.mean},
    'ActivityTimeLightFair': {'mean': np.mean},
    'ActivityTimeFair': {'mean': np.mean},
    'ActivityTimeFairVigorous': {'mean': np.mean},
    'ActivityTimeVigorous': {'mean': np.mean},
    'SleepTimeAsleep': {'mean': np.mean},
    'SleepTimeAwake': {'mean': np.mean},
    'SleepTimeRestless': {'mean': np.mean},
    'SleepTimeAsleepAwake': {'mean': np.mean},
    'SleepTimeAwakeRestless': {'mean': np.mean},
    'SleepTimeAsleepRestless': {'mean': np.mean},
    'SleepTimeTotal': {'mean': np.mean},
    'HeartRateMean': {'mean': np.mean},
    'HeartRateMedian': {'median': np.median},
```

```
    'HeartRateMin': {'min': np.min},
    'HeartRateMax': {'max': np.max},
    'HeartRateStdev': {'std': np.std},
}

target_column_weekly_aggregation_functions = {
    'StepsCount': {'mean': np.mean},
    'ActivityTimeSedentaryAwake': {'mean': np.mean},
    'ActivityTimeSedentaryLightAwake': {'mean': np.mean},
    'ActivityTimeLight': {'mean': np.mean},
    'ActivityTimeLightFair': {'mean': np.mean},
    'ActivityTimeFair': {'mean': np.mean},
    'ActivityTimeFairVigorous': {'mean': np.mean},
    'ActivityTimeVigorous': {'mean': np.mean},
    'SleepTimeAsleep': {'mean': np.mean},
    'SleepTimeAwake': {'mean': np.mean},
    'SleepTimeRestless': {'mean': np.mean},
    'SleepTimeAsleepAwake': {'mean': np.mean},
    'SleepTimeAwakeRestless': {'mean': np.mean},
    'SleepTimeAsleepRestless': {'mean': np.mean},
    'SleepTimeTotal': {'mean': np.mean},
    'HeartRateMean': {'mean': np.mean},
    'HeartRateMedian': {'median': np.median},
    'HeartRateMin': {'min': np.min},
    'HeartRateMax': {'max': np.max},
    'HeartRateStdev': {'std': np.std},
}

target_column_daily_aggregation_functions = {
    'StepsCount': {'sum': np.sum},
    'ActivityTimeSedentaryAwake': {'sum': np.sum},
    'ActivityTimeSedentaryLightAwake': {'sum': np.sum},
    'ActivityTimeLight': {'sum': np.sum},
    'ActivityTimeLightFair': {'sum': np.sum},
    'ActivityTimeFair': {'sum': np.sum},
    'ActivityTimeFairVigorous': {'sum': np.sum},
```

```
        'ActivityTimeVigorous': {'sum': np.sum},
        'SleepTimeAsleep': {'mean': np.mean},
        'SleepTimeAwake': {'mean': np.mean},
        'SleepTimeRestless': {'mean': np.mean},
        'SleepTimeAsleepAwake': {'mean': np.mean},
        'SleepTimeAwakeRestless': {'mean': np.mean},
        'SleepTimeAsleepRestless': {'mean': np.mean},
        'SleepTimeTotal': {'sum': np.sum},
        'HeartRateMean': {'mean': np.mean},
        'HeartRateMedian': {'median': np.median},
        'HeartRateMin': {'min': np.min},
        'HeartRateMax': {'max': np.max},
        'HeartRateStdev': {'std': np.std},
    }
```

R

main.r

```
library(survival)

behaviours <- c(
 #'StepsCount',
 #'ActivityTimeLight',
 #'ActivityTimeLightFair',
 #'ActivityTimeFair',
 #'ActivityTimeFairVigorous',
 #'ActivityTimeVigorous',
 #'SleepTimeTotal',
 #'HeartRateMean',
 #'HeartRateMedian',
 #'HeartRateMin',
 #'HeartRateMax',
 #'HeartRateStdev',
 #'SleepTimeAsleep',
 #'SleepTimeAwake',
```

```
 #'SleepTimeRestless',
 #'SleepTimeAsleepAwake',
 #'SleepTimeAwakeRestless',
 #'SleepTimeAsleepRestless',
 #'SleepTimeTotal',
 #'SleepTimeTotalKnown'
)

period_durations <- c(1, 2, 3, 4, 5, 6, 7, 8)
aggregates <- c("sum", "max", "min", "mean", "median", "stdev")
flavours <- c("separate", "cumulative")

# First grid search.
# minimum_available_data_ratios_daily_sedentary <- c("0.9583333333333334", "0.75", "0.875")

# Second grid search.
# minimum_available_data_ratios_daily_sedentary <- c("0.041666666666666664", "0.020833333333333332", "0.010416666666666666")

# Third grid search.
minimum_available_data_ratios_daily_sedentary <- c("0.08333333333333333", "0.16666666666666666", "0.3333333333333333", "0.6666666666666666")

minimum_available_data_ratios_weekly <- c("0.51", "0.76", "0.99")
minimum_available_data_ratios_period <- c("0.51", "0.76", "0.99")

allows_zeros <- c("False")
allows_no_event_participants <- c("True")

regression_eps <- 0.000001
significance_threshold <- 0.05
min_or <- 0.001
max_or <- 1000

sink(file <- "data/conditional_logit_periods.html")

print(noquote('<html><body><table border=1 valign=top>'))
print(noquote('<tr><th colspan=2>Daily</th>'))
```

```
for (minimum_available_data_ratio_daily_active in minimum_available_data_ratios_daily_sedentary) {
 print(noquote(sprintf('<th colspan=%d>%s</th>', length(minimum_available_data_ratios_weekly), minimum_available_data_ratio_daily_active)))
}

print(noquote('</tr>'))

print(noquote('<tr><th colspan=2>Weekly</th>'))

for (minimum_available_data_ratio_daily_active in minimum_available_data_ratios_daily_sedentary) {
 for (minimum_available_data_ratio_weekly in minimum_available_data_ratios_weekly) {
   print(noquote(sprintf('<th>%s</th>', minimum_available_data_ratio_weekly)))
 }
}

print(noquote('</tr>'))

print(noquote('<tr><th colspan=2>Period</th>'))

for (minimum_available_data_ratio_daily_active in minimum_available_data_ratios_daily_sedentary) {
 for (minimum_available_data_ratio_weekly in minimum_available_data_ratios_weekly) {
   for (minimum_available_data_ratio_period in minimum_available_data_ratios_period) {
     if (minimum_available_data_ratio_weekly == minimum_available_data_ratio_period) {
       print(noquote(sprintf('<th>%s</th>', minimum_available_data_ratio_period)))
     }
   }
 }
}

print(noquote('</tr>'))

for (allow_zeros in allows_zeros) {
 for (allow_no_event_participants in allows_no_event_participants) {
   print(noquote(sprintf('<tr><th>%s</th><th>%s</th>', allow_zeros, allow_no_event_participants)))

   for (minimum_available_data_ratio_daily_active in minimum_available_data_ratios_daily_sedentary) {
```

```
    for (minimum_available_data_ratio_weekly in minimum_available_data_ratios_weekly) {
      for (minimum_available_data_ratio_period in minimum_available_data_ratios_period) {
        if (minimum_available_data_ratio_weekly == minimum_available_data_ratio_period) {
          print(noquote('<td>'))
           for (flavour in flavours) {
            mentioned_flavour <- 0
             for (behaviour in behaviours) {
              has_behaviour <- 0
              p_value <- 1000000.0
              odds_ratio <- 0.0
              all_period_found <- c(FALSE, FALSE, FALSE, FALSE, FALSE, FALSE, FALSE, FALSE)
              all_period_min_effects <- c(1000000.0, 1000000.0, 1000000.0, 1000000.0, 1000000.0, 1000000.0, 1000000.0, 1000000.0)
              all_period_max_effects <- c(-1000000.0, -1000000.0, -1000000.0, -1000000.0, -1000000.0, -1000000.0, -1000000.0, -1000000.0)
              all_period_effects_min_pvalues <- c(0.0, 0.0, 0.0, 0.0, 0.0, 0.0, 0.0, 0.0)
              all_period_models_min_pvalues <- c("-", "-", "-", "-", "-", "-", "-", "-")
              all_period_min_pvalues <- c(1000000.0, 1000000.0, 1000000.0, 1000000.0, 1000000.0, 1000000.0, 1000000.0, 1000000.0)
              min_effect <- 1000000.0
              max_effect <- -1000000.0
               for (daily_aggregate in aggregates) {
                if (min_effect < -regression_eps && max_effect > regression_eps) {
                  break
                }

                for (weekly_aggregate in aggregates) {
                  if (min_effect < -regression_eps && max_effect > regression_eps) {
                    break
                  }

                  for (period_aggregate in aggregates) {
                    if (min_effect < -regression_eps && max_effect > regression_eps) {
                      break
                    }

                    for (period_duration in period_durations) {
                      if (min_effect < -regression_eps && max_effect > regression_eps) {
                        break
```

```
                        }

                        dataset_file_name_flavour_format <-
paste('data/result_data/dataset_%s_period_%i_behaviour_%s_daily_%s_weekly_%s_period_%s_mindaily_%s_minweekly_%s_minperiod_%s_zeros_%s_paired_%s.csv')
                        dataset_file_name <- sprintf(dataset_file_name_flavour_format, flavour, period_duration, behaviour,
                                                     daily_aggregate, weekly_aggregate, period_aggregate,
                                                     minimum_available_data_ratio_daily_active, minimum_available_data_ratio_weekly,
minimum_available_data_ratio_period,
                                                     allow_zeros, allow_no_event_participants)

                        if (file.exists(dataset_file_name)) {
                          data <- read.csv(file <- dataset_file_name)
                          data <- transform(data, has_event_numeric=ifelse(has_event=="True", 1, 0))

                          model <- clogit(has_event_numeric ~ value + factor(season) + factor(week_day_weekend_day), data=data, method="approximate")

                          summary <- summary(model)
                          pvalue <- summary$coefficients[1, 5]
                          param <- summary$coefficients[1, 1]

                          if (!is.na(pvalue) && !is.na(param)) {
                            if (pvalue < 0) {
                              print(noquote(summary))
                              stop("P-value should be positive")
                            }

                            if (pvalue < significance_threshold) {
                              has_behaviour <- 1

                              min_effect <- min(min_effect, param)
                              max_effect <- max(max_effect, param)
                              all_period_found[period_duration] <- TRUE
                              all_period_min_effects[period_duration] <- min(all_period_min_effects[period_duration], param)
                              all_period_max_effects[period_duration] <- max(all_period_max_effects[period_duration], param)

                              p_value <- min(p_value, pvalue)
```

```
    if (pvalue < all_period_min_pvalues[period_duration]) {
      all_period_min_pvalues[period_duration] <- pvalue
      all_period_effects_min_pvalues[period_duration] <- param
      model_name <- "S+WD/WE"
      all_period_models_min_pvalues[period_duration] <- model_name
    }
  }
}

model <- clogit(has_event_numeric ~ value + factor(season) + factor(week_day), data=data, method="approximate")

summary <- summary(model)
pvalue <- summary$coefficients[1, 5]
param <- summary$coefficients[1, 1]

if (!is.na(pvalue) && !is.na(param)) {
  if (pvalue < 0) {
    print(noquote(summary))
    stop("P-value should be positive")
  }

  if (pvalue < significance_threshold) {
    has_behaviour <- 1

    min_effect <- min(min_effect, param)
    max_effect <- max(max_effect, param)
    all_period_found[period_duration] <- TRUE
    all_period_min_effects[period_duration] <- min(all_period_min_effects[period_duration], param)
    all_period_max_effects[period_duration] <- max(all_period_max_effects[period_duration], param)

    p_value <- min(p_value, pvalue)

    if (pvalue < all_period_min_pvalues[period_duration]) {
      all_period_min_pvalues[period_duration] <- pvalue
      all_period_effects_min_pvalues[period_duration] <- param
```

```
      model_name <- "S+WD"
      all_period_models_min_pvalues[period_duration] <- model_name
    }
  }
}

model <- clogit(has_event_numeric ~ value + factor(season), data=data, method="approximate")

summary <- summary(model)
pvalue <- summary$coefficients[1, 5]
param <- summary$coefficients[1, 1]

if (!is.na(pvalue) && !is.na(param)) {
  if (pvalue < 0) {
    print(noquote(summary))
    stop("P-value should be positive")
  }

  if (pvalue < significance_threshold) {
    has_behaviour <- 1

    min_effect <- min(min_effect, param)
    max_effect <- max(max_effect, param)
    all_period_found[period_duration] <- TRUE
    all_period_min_effects[period_duration] <- min(all_period_min_effects[period_duration], param)
    all_period_max_effects[period_duration] <- max(all_period_max_effects[period_duration], param)

    p_value <-min(p_value, pvalue)

    if (pvalue < all_period_min_pvalues[period_duration]) {
      all_period_min_pvalues[period_duration] <- pvalue
      all_period_effects_min_pvalues[period_duration] <- param
      model_name <- "S"
      all_period_models_min_pvalues[period_duration] <- model_name
    }
  }
```

```
                }
              }
            }
          }
        }
      }

      if (has_behaviour == 1) {
        strike <- 0

        if (min_effect < -regression_eps && max_effect > regression_eps) {
          strike <- 1
        }

        sign <- "?"
        odds_ratio <- 1.0
        odds_ratio_min_pvalue <- 1.0

        if (min_effect < -regression_eps) {
          sign <- "-"
          odds_ratio <- exp(min_effect)
        }

        if (max_effect > regression_eps) {
          sign <- "+"
          odds_ratio <- exp(max_effect)
        }

        if (odds_ratio > min_or && odds_ratio < max_or) {
          if (mentioned_flavour == 0) {
            print(noquote(sprintf('<b>%s</b><br />', flavour)))
            mentioned_flavour <- 1
          }

          print(noquote(sprintf('%s: %s<br />', behaviour, sign)))
```

```
                    for(i in period_durations) {
                      if (isTRUE(all_period_found[i])) {
                        extreme_or_pvalue <- exp(all_period_effects_min_pvalues[i])
                        extreme_pvalue <- all_period_min_pvalues[i]
                        extreme_model_pvalue <- all_period_models_min_pvalues[i]

                        extreme_or <- 1.0

                        if (min_effect < -regression_eps) {
                          extreme_or <- exp(all_period_min_effects[i])
                        }

                        if (max_effect > regression_eps) {
                          extreme_or <- exp(all_period_max_effects[i])
                        }

                        if (strike == 1) {
                          print(noquote(sprintf('<del><b>%s weeks</b>: or=%.3f (or.p=%.3f, p=%.3f, m=%s)</del><br />', i, extreme_or, extreme_or_pvalue,
extreme_pvalue, extreme_model_pvalue)))
                        } else {
                          print(noquote(sprintf('<b>%s</b>: or=%.3f (or.p=%.3f, p=%.3f, m=%s)<br />', i, extreme_or, extreme_or_pvalue, extreme_pvalue,
extreme_model_pvalue)))
                        }
                      }
                    }
                  }
                }
              }
            }

           print(noquote('</td>'))
          }
        }
      }
    }
```

```
   print(noquote('</tr>'))
 }
}

print(noquote('</table></body></html>'))

sink(file <- NULL)

print("Done")
```
